# Supplementary material for: Legionella pneumophila regulates host cell motility by targeting Phldb2 with a 14-3-3ζ-dependent protease effector
Source: eLife. 2022 Feb 17;11:e73220. doi: 10.7554/eLife.73220 (PMC8871388; doi:10.7554/eLife.73220)
Supplement: Supplementary file 1. [file elife-73220-supp1.docx]

Table S1 Potential Lem8 interacting proteins identified by yeast two-hybrid screenings

| Gene name | protein | Hit times | Verification by IP |
| --- | --- | --- | --- |
| 14-3-3ζ | 14-3-3ζ | 50 | Yes |
| BAD97321.1 | DnaJ homolog subfamily B member 6 | 13 | ND |
| ACI04420.1 | NADH dehydrohenase subunit 1 | 5 | ND |
| NP_005944.1 | Metallothiolein-2 | 5 | ND |
| BAA00525.1 | Glutathione peroxidase | 3 | ND |
| BAK23994.1 | Cytochrome b | 3 | ND |
| NP_001186913.1 | NADH-ubiquinone oxireductase 75 kda | 2 | ND |
| NP_001139660.1 | TOX high motility group box family member 3 | 2 | No |
| NP_001136153.1 | Chitinase domain-containing protein 1 | 2 | ND |
| NP_001121164.1 | Propinyl-coA carboxylase a-chain | 2 | ND |
| NP_115982.1 | Histidine triad nucleotide binding protein | 2 | ND |
| NP_072047.4 | Vacuolar protein sorting 52 homolog | 2 | No |
| AAP97148.1 | AF086918_1 E3-13 | 1 | ND |
| NP_001004067.1 | Nodal modulator 3 precursor | 1 | ND |

Note: Shown are 93 clones that harbored identifiable proteins from 96 sequenced candidates.

ND: not determined
