# Supplementary material for: Legionella pneumophila regulates host cell motility by targeting Phldb2 with a 14-3-3ζ-dependent protease effector
Source: eLife. 2022 Feb 17;11:e73220. doi: 10.7554/eLife.73220 (PMC8871388; doi:10.7554/eLife.73220)
Supplement: Supplementary file 2. [file elife-73220-supp2.docx]

Table S2 Bacterial strains, plasmids and primers used in the study

| **Bacterial Strains** | **Source** | **Identifier** |
| --- | --- | --- |
| *L. pneumophila* (Philadelphia-1) LP02 | (Berger and Isberg, 1993) | N/A |
| *L. pneumophila* LP03 | (Berger and Isberg, 1993) | N/A |
| LP02∆*lem8* | This study | N/A |
| LP02∆*lem8*(pZL507) | This study | N/A |
| LP02∆*lem8*(pLem8) | This study | N/A |
| LP02∆*lem8*(pLem8_C280S_) | This study | N/A |
| LP02∆*lem8*(pLem8_L58G/E59G_) | This study | N/A |
| *E.coli* BL21(DE3) | Transgen | CAT# CD601 |
| *E.coli* XL1-Blue | Transgen | CAT# CD401 |

| **Plasmids** | **Source** | **Identifier** |
| --- | --- | --- |
| pZL507 | (Qiu et al., 2016) | N/A |
| pZL507::*lem8* | This study | N/A |
| pZL507::*lem8_C280S_* | This study | N/A |
| pZL507::*lem8_L58G/E59G_* | This study | N/A |
| pXDC61m::*lem8* | This study | N/A |
| pAPH | (Song et al., 2021) | N/A |
| pAPH::*GFP* | This study | N/A |
| pAPH::*lem8* | This study | N/A |
| pAPH::*lem8_C280S_* | This study | N/A |
| pAPH::*lem8_H391A_* | This study | N/A |
| pAPH::*lem8_D412A_* | This study | N/A |
| pAPH::*lem8_L58G/E59G_* | This study | N/A |
| pAPH::*lem8*∆*N25* | This study | N/A |
| pAPH::*lem8*∆*N50* | This study | N/A |
| pAPH::*lem8*∆*C50* | This study | N/A |
| pAPH::*lem8*∆*C52* | This study | N/A |
| pAPH::*lem8*∆*C100* | This study | N/A |
| pAPH::*PHLDB2-Flag* | This study | N/A |
| pAPH::*PHLDB2_R1101/Q1102A_-Flag* | This study | N/A |
| pAPH::*PHLDB2_Q1102/R1103A_-Flag* | This study | N/A |
| pAPH::*lem8*-*GFP* | This study | N/A |
| pAPH::*lem8_C280S_*-*GFP* | This study | N/A |
| pAPH::*lem8*∆*C52*-*GFP* | This study | N/A |
| pAPH::*lem8_L58G/E59G_*-*GFP* | This study | N/A |
| pAPH::*ankrd13B* | This study | N/A |
| pAPH::*gnal* | This study | N/A |
| pcDNA3.1+N-Flag | GenScript | N/A |
| pcDNA3.1+N-Flag-*pak6* | This study | N/A |
| pcDNA3.1+N-Flag-*chkB* | This study | N/A |
| pcDNA3.1+N-Flag-*exoc8* | This study | N/A |
| pcDNA3.1+N-Flag-*kiaa1033* | This study | N/A |
| pcDNA3.1+N-Flag-*ppp6r1* | This study | N/A |
| pcDNA3.1+N-Flag-*rasgap2* | This study | N/A |
| pcDNA3.1+N-Flag-*gpr61* | This study | N/A |
| pEGFP-C1 | Clontech | CAT#6084-1 |
| GFP::*lem8* | This study | N/A |
| GFP::*PHLBD2* | This study | N/A |
| GFP::*PHLBD2*∆*N50* | This study | N/A |
| GFP::*PHLBD2*∆*N100* | This study | N/A |
| GFP::*PHLBD2*∆*N200* | This study | N/A |
| pEGFP-N1 | Clontech | CAT#6085-1 |
| *PHLBD2*∆*C100*::GFP | This study | N/A |
| *PHLBD2*∆*C153*::GFP | This study | N/A |
| pmCherry-C1 | Clontech | CAT#632524 |
| mCherry-C1::*lem8* | This study | N/A |
| mCherry-C1::*lem8_C280S_* | This study | N/A |
| mCherry-C1::*lem8_H391A_* | This study | N/A |
| mCherry-C1::*lem8_D412A_* | This study | N/A |
| mCherry-C1::*lem8*∆*N25* | This study | N/A |
| mCherry-C1::*lem8*∆*C52* | This study | N/A |
| mCherry-C1::*lem8*∆*C100* | This study | N/A |
| pQE30 | Qiagen | CAT#32915 |
| pQE30::*mmu-YWHAZ* | This study | N/A |
| pQE30::*lem8* | This study | N/A |
| pQE30::*lem8*_C280S_ | This study | N/A |
| pQE30::*lem8*_H391A_ | This study | N/A |
| pQE30::*lem8*_D412A_ | This study | N/A |
| pQE30::*lem8*_P473A/Q474A/R475A/Q476A_ | This study | N/A |
| pQE30::*lem8*∆*C52* | This study | N/A |
| pGEX6p-1 | Cytiva | CAT#28-9546-48 |
| pGEX6p-1::*mmu-YWHAZ* | This study | N/A |
| pGEX6p-1::*hsa-YWHAZ* | This study | N/A |
| pGEX6p-1::*ddi-fttB* | This study | N/A |
| pCDH-CMV-MCS-EF1-Puro | System Biosciences | CAT#CD510B-1 |
| pCDH-CMV-MCS-EF1-Puro::*GFP* | This study | N/A |
| pCDH-CMV-MCS-EF1-Puro::*GFP-Lem8* | This study | N/A |
| pCDH-CMV-MCS-EF1-Puro::*GFP-Lem8_C280S_* | This study | N/A |
| pYES2CT | Invitrogen | CAT#V825120 |
| pYES2CT::*lem8* | This study | N/A |
| pYES2CT::*lem8_C280S_* | This study | N/A |
| pYES2CT::*lem8_H391A_* | This study | N/A |
| pYES2CT::*lem8*_D412A_ | This study | N/A |
| pYES2CT::*lem8_L58G/E59G_* | This study | N/A |
| pGADGH | Clontech | CAT#638853 |
| pGADGH::*mmu-YWHAZ* | This study | N/A |
| pGBKT7 | Clontech | CAT#630489 |
| pGBKT7::*lem8_C280S_* | This study | N/A |
| pMD2.G | Addgene | Cat#12259 |
| psPAX2 | Addgene | Cat#12260 |

| **Primers** | **Sequence (Restriction enzyme sites are underlined)** | **Note** |
| --- | --- | --- |
| pSL1001 | cgcggatccatgcctcaaatcctaaatg | *lem8* 5F BamHI |
| pSL1002 | acgcgtcgacttaaataggaaatcctcttgtatttc | *lem8* 3R SalI |
| pSL1003 | ctggagctcaataaattattttttcgactcaaggaagg | *lem8* up 5F SacI  knockout |
| pSL1004 | cattctcagtgtcagcttcaatgacagcc | *lem8* up 3R  knockout |
| pSL1005 | tgaagctgacactgagaatgagcaaagcag | *lem8* down 5F knockout |
| pSL1006 | acgcgtcgacgataggctggctccatgg | *lem8* down 3R SalI knockout |
| pSL1007 | cctagctaagccgtgactcataccactctttaaac | *lem8_C280S_*-1 |
| pSL1008 | gtttaaagagtggtatgagtcacggcttagctagg | *lem8_C280S_*-2 |
| pSL1009 | gtttgtggatagcaacaacagctccagaactgctattagcatagc | *lem8_H391A_*-1 |
| pSL1010 | gctatgctaatagcagttctggagctgttgttgctatccacaaac | *lem8_H391A_*-2 |
| pSL1011 | gcatccaacctgcattagcagcaaaatagtcgatgatgaaa | *lem8_D412A_*-1 |
| pSL1012 | tttcatcatcgactattttgctgctaatgcaggttggatgc | *lem8_D412A_*-2 |
| pSL1013 | ttttcaatgaagtagataattgtccgccttgctctttcatggtttttactgctaactc | *lem8_L58G/E59G_*-1 |
| pSL1014 | gagttagcagtaaaaaccatgaaagagcaaggcggacaattatctacttcattgaaaa | *lem8_L58G/E59G_*-2 |
| pSL1015 | gtttctgctgacaagctctgtgctcttgctgccgcagcagtaggttgtggtgctttttcacagag | *lem8*_P473A/Q474A/R475A/Q476A_-1 |
| pSL1016 | ctctgtgaaaaagcaccacaacctactgctgcggcagcaagagcacagagcttgtcagcagaaac | *lem8*_P473A/Q474A/R475A/Q476A_-2 |
| pSL1017 | cgcggatccattgttgataaatactctgaca | *lem8∆N25* 5F BamHI |
| pSL1018 | cgcggatccgtaaaaaccatgaaagagca | *lem8∆N50* 5F BamHI |
| pSL1019 | acgcgtcgacttatgctctttgtctctgagg | *lem8∆C50* 3R SalI |
| pSL1020 | acgcgtcgacttattgtctctgaggagtagg | *lem8∆C52* 3R SalI |
| pSL1021 | acgcgtcgacttactcttgaaaactcttatcgt | *lem8∆C100* 3R SalI |
| pSL1022 | acgcgtcgacaataggaaatcctcttgtatttc | *lem8-GFP* 3R SalI |
| pSL1023 | acgcgtcgacttgtctctgaggagtagg | *lem8∆C52-GFP* 3R SalI |
| pSL1024 | cgcggatccatggataaaaatgagctggt | *mmu-YWHAZ* 5F BclI |
| pSL1025 | acgcgtcgacttaattttcccctccttctc | *mmu-YWHAZ* 3R SalI |
| pSL1026 | cgcggatccatggataaaaatgagctggt | *hsa-YWHAZ* 5F BamHI |
| pSL1027 | acgcgtcgacttaattttcccctccttctc | *hsa-YWHAZ* 3R XhoI |
| pSL1028 | cgcggatccatgaccagagaagaaaatgt | *ddi-fttB* 5F BamHI |
| pSL1029 | acgcgtcgacttacattcctggttcattttg | *ddi-fttB* 3R SalI |
| pSL1030 | cgcggatccatggaagagcatagctaca | *hsa-phldb2* 5F BamHI |
| pSL1031 | acgcgtcgacctacaacaagaagtgagtgt | *hsa-phldb2* 3R SalI |
| pSL1032 | cgcggatccatggccaatggagactattctg | *phldb2∆N50* 5F BamHI |
| pSL1033 | cgcggatccatgaaaaatattcctatgaaacctccaa | *phldb2∆N100* 5F BamHI |
| pSL1034 | cgcggatccatgccttcaagccca | *phldb2∆N200* 5F BamHI |
| pSL1035 | acgcgtcgaccaacaagaagtgagtgtaac | *phldb2-Flag* 3R SalI |
| pSL1036 | aaggacgagcctgtgcccttgctgcctcccttatttttacttccttttct | *phldb2_R1101/Q1102A_*-1 |
| pSL1037 | agaaaaggaagtaaaaataagggaggcagcaagggcacaggctcgtcctt | *phldb2_R1101/Q1102A_*-2 |
| pSL1038 | aggacgagcctgtgccgctgctctctcccttatttttacttccttttctatt | *phldb2_Q1102/R1103A_*-1 |
| pSL1039 | aatagaaaaggaagtaaaaataagggagagagcagcggcacaggctcgtcct | *phldb2_Q1102/R1103A_*-2 |
| pSL1040 | acgcgtcgacatggtgagcaagggcga | *GFP* 5F SalI |
| pSL1041 | ataagaatgcggccgccttgtacagctcgtccatgc | *GFP* 3R NotI |
| pSL1042 | acgcgtcgactgcattttgatgaggaatcc | *phldb2∆C100* 3R SalI |
| pSL1043 | acgcgtcgactgctcccttatttttacttccttttc | *phldb2∆C153* 3R SalI |
| pSL1044 | ccggaattcatgatccccgccaac | *hsa-ankrd13B* 5F EcoRI |
| pSL1045 | acgcgtcgacctactgctcggtcagtg | *hsa-ankrd13B* 3R SalI |
| pSL1046 | ggaagatctatgggtctgtgctacagt | *hsa-gnal* 5F BglII |
| pSL1047 | acgcgtcgactcacaagagctcatactgc | *hsa-gnal* 3R SalI |
| pSL1048 | cggggtaccatgttccgcaagaaaaagaagaaac | *hsa-pak6* 5F KpnI |
| pSL1049 | acgcgtcgactcagcaggtggaggtctg | *hsa-pak6* 3R SalI |
| pSL1050 | cgcggatccatggcggccgaggc | *hsa-chkB* 5F BamHI |
| pSL1051 | acgcgtcgactcaggatgaggagtggacactg | *hsa-chkB* 3R SalI |
| pSL1052 | cggggtaccatggcgatggcgatgtc | *hsa-exoc8* 5F KpnI |
| pSL1053 | acgcgtcgacttagaccactgatgttgtactttca | *hsa-exoc8* 3R SalI |
| pSL1054 | cgcggatccatggcggtggagactctg | *hsa-kiaa1033* 5F BamHI |
| pSL1055 | acgcgtcgactcatttcacaacaggatcagcag | *hsa-kiaa1033* 3R SalI |
| pSL1056 | ggaagatctatgttttggaagtttgacctgca | *hsa-ppp6r1* 5F BglII |
| pSL1057 | acgcgtcgacctattgggagcctgggga | *hsa-ppp6r1* 3R SalI |
| pSL1058 | cgcggatccatggcaggcaccctgga | *hsa-rasgap2* 5F BamHI |
| pSL1059 | acgcgtcgacttacaagtggatgtcaaacacccca | *hsa-rasgap2* 3R SalI |
| pSL1060 | cgcggatccatggagtcctcacccatc | *hsa-gpr61* 5F BamHI |
| pSL1061 | acgcgtcgactcatgactccagccgg | *hsa-gpr61* 3R SalI |

**Reference**

Berger, K.H., and Isberg, R.R. (1993). Two distinct defects in intracellular growth complemented by a single genetic locus in Legionella pneumophila. Mol Microbiol *7*, 7-19. 10.1111/j.1365-2958.1993.tb01092.x.

Qiu, J., Sheedlo, M.J., Yu, K., Tan, Y., Nakayasu, E.S., Das, C., Liu, X., and Luo, Z.Q. (2016). Ubiquitination independent of E1 and E2 enzymes by bacterial effectors. Nature *533*, 120-124. 10.1038/nature17657.

Song, L., Xie, Y., Li, C., Wang, L., He, C., Zhang, Y., Yuan, J., Luo, J., Liu, X., Xiu, Y., et al. (2021). The Legionella Effector SdjA Is a Bifunctional Enzyme That Distinctly Regulates Phosphoribosyl Ubiquitination. mBio, e0231621. 10.1128/mBio.02316-21.
