# Supplementary material for: Legionella pneumophila regulates host cell motility by targeting Phldb2 with a 14-3-3ζ-dependent protease effector
Source: eLife. 2022 Feb 17;11:e73220. doi: 10.7554/eLife.73220 (PMC8871388; doi:10.7554/eLife.73220)

Figure 1-source data 1

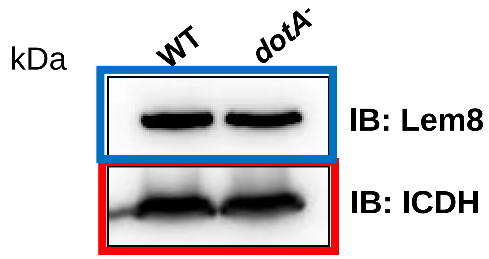

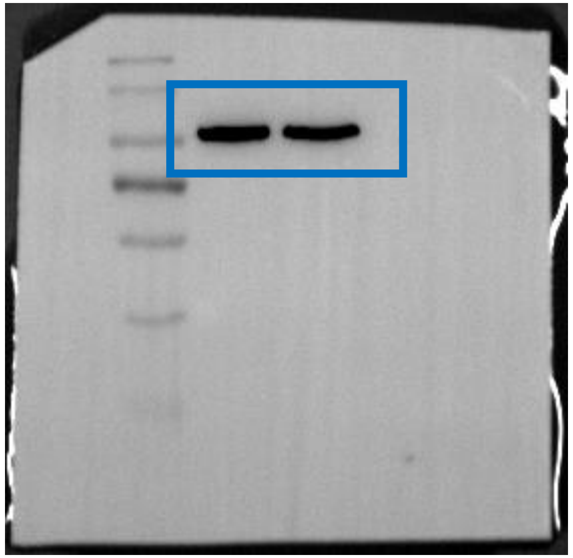

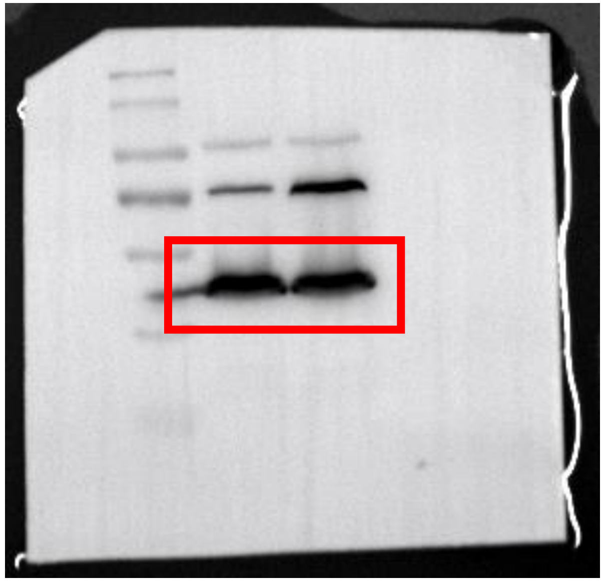

Figure 1-source data 2

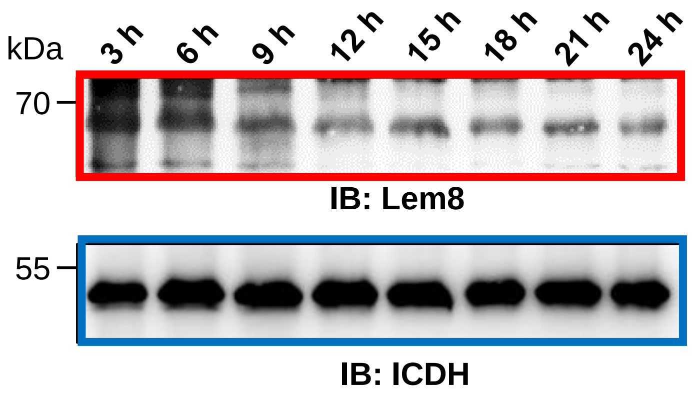

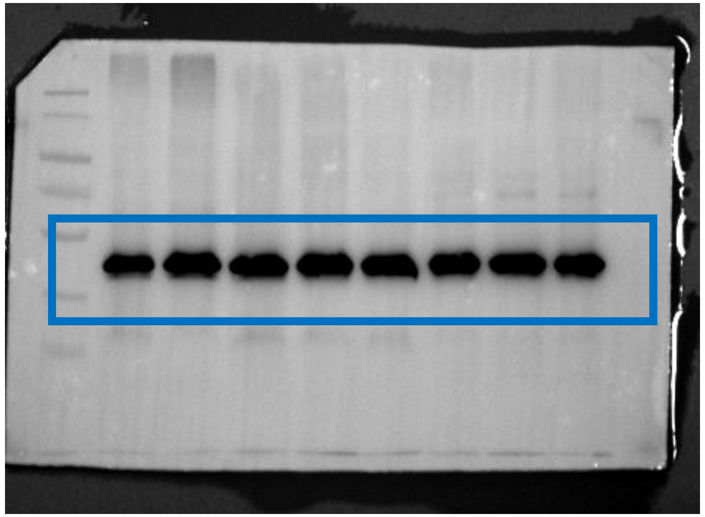

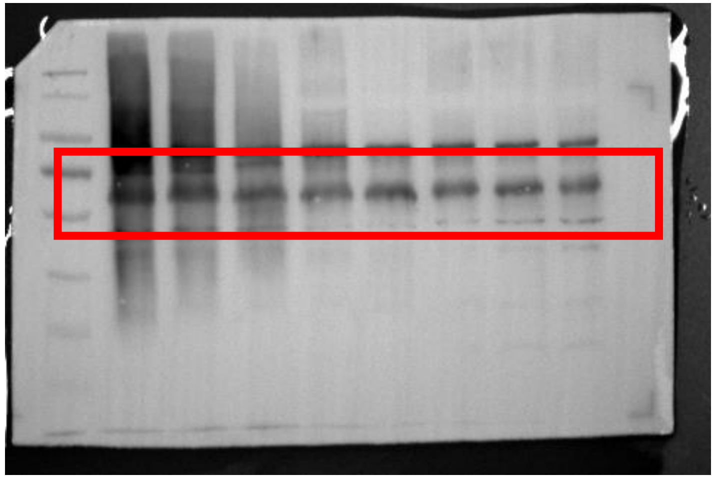

Figure 1-source data 3

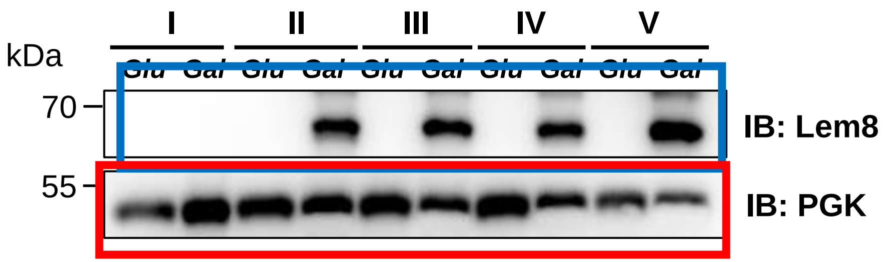

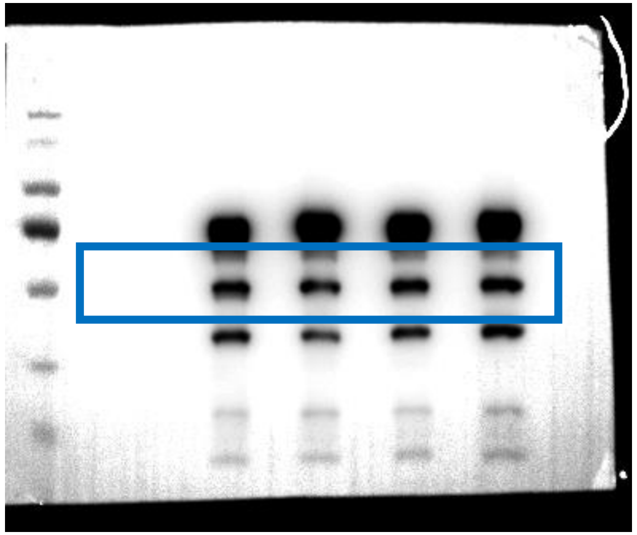

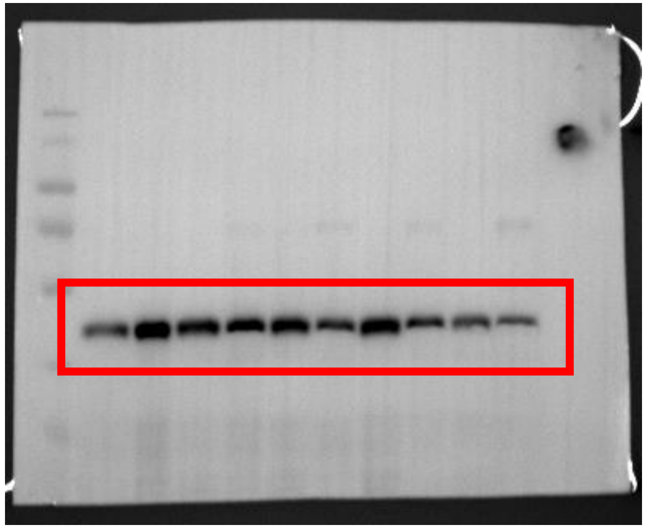

Figure 1-figure supplement 2-source data 1

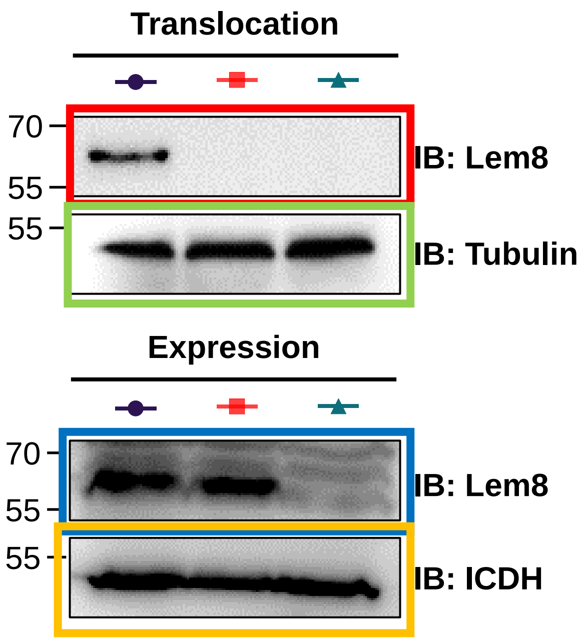

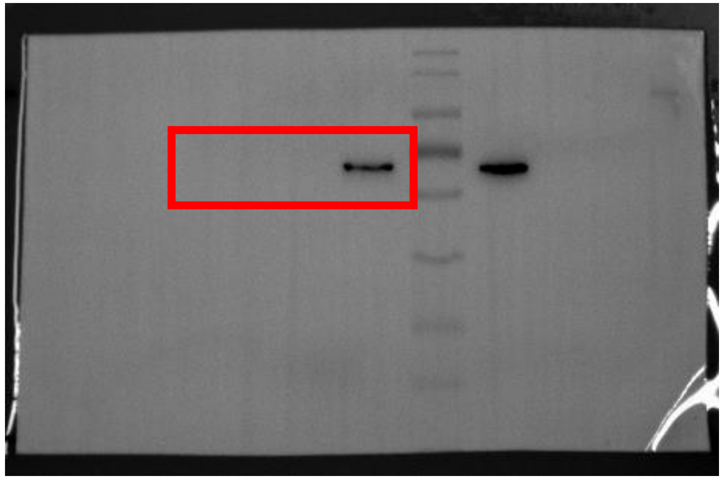

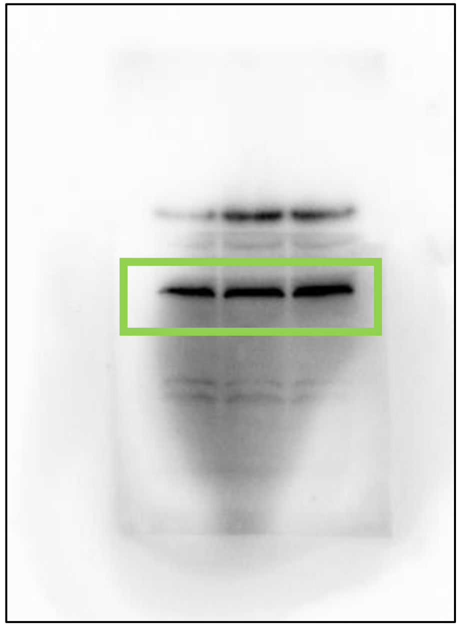

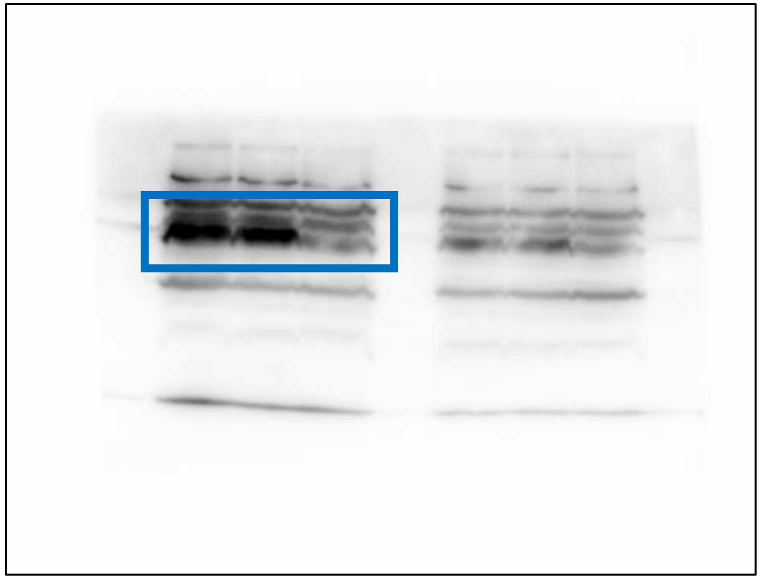

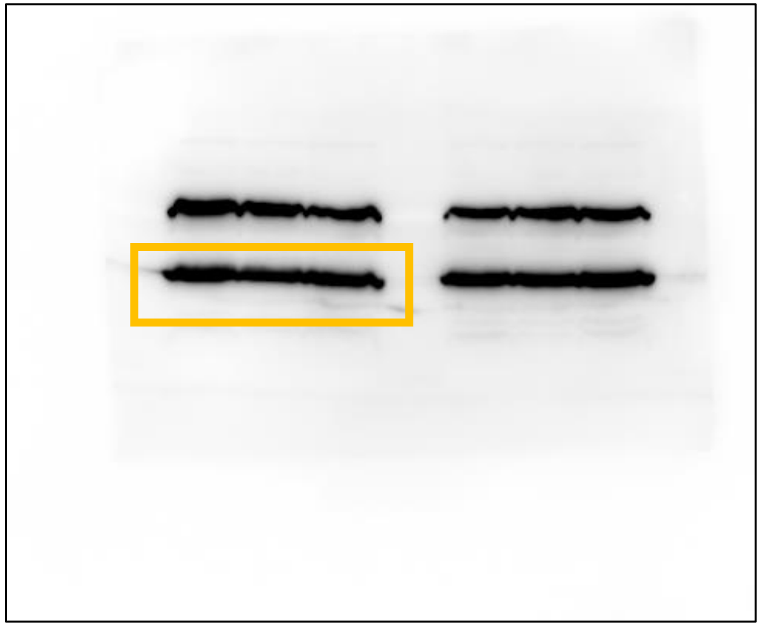

Figure 2-source data 1

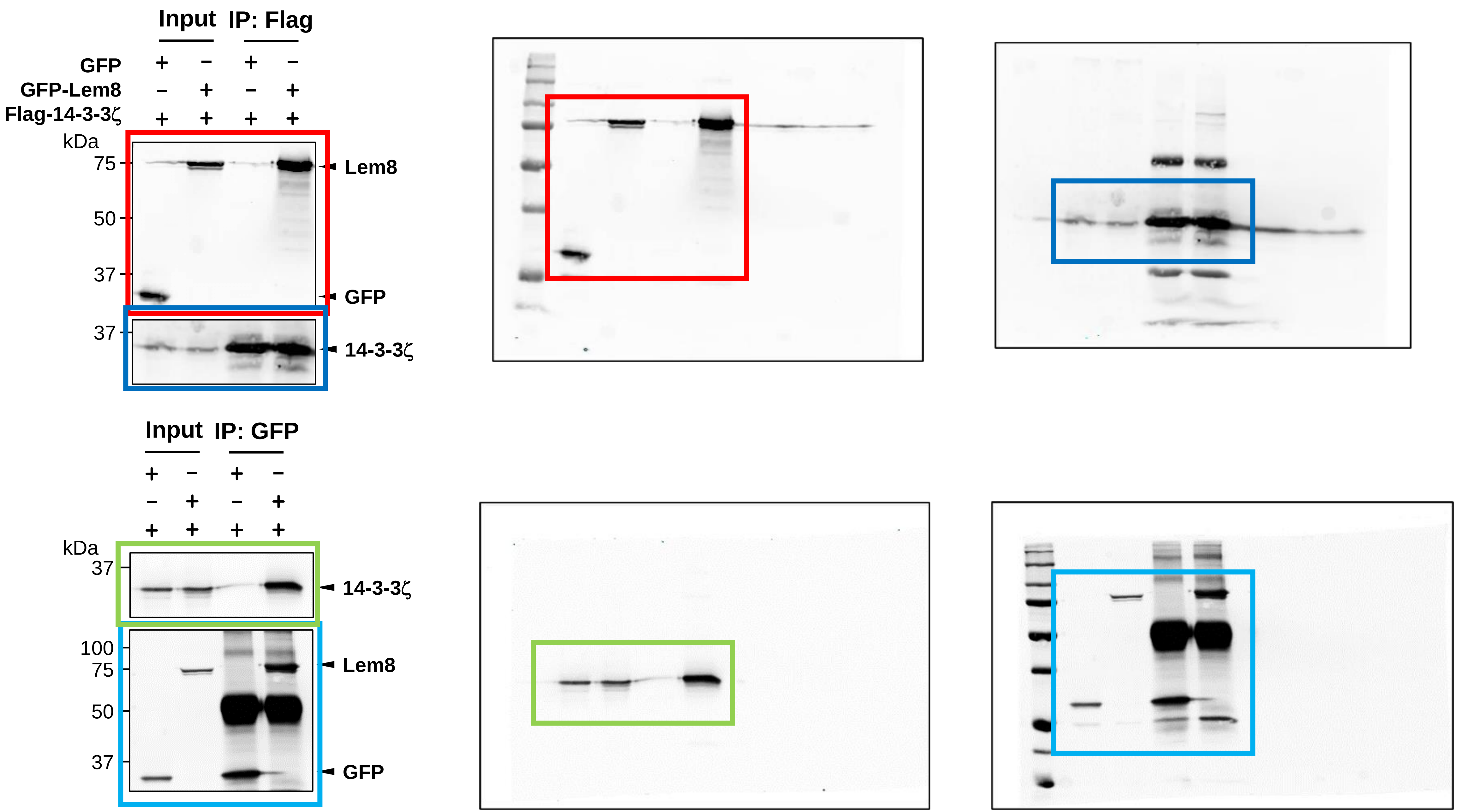

Figure 2-source data 2

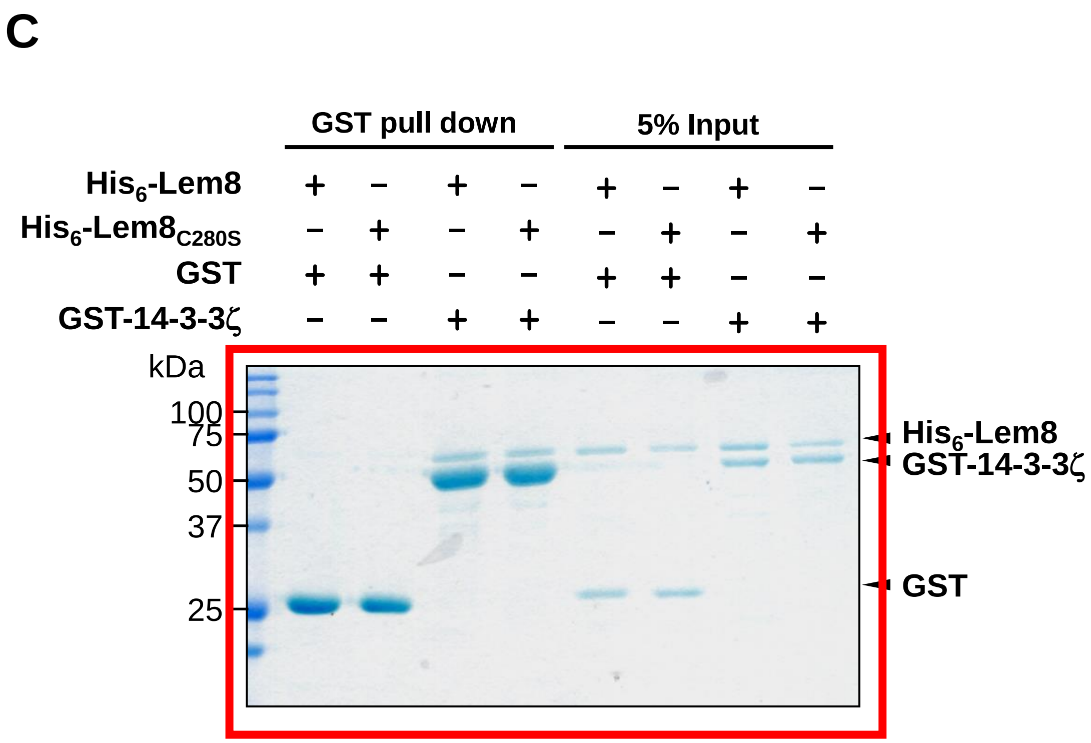

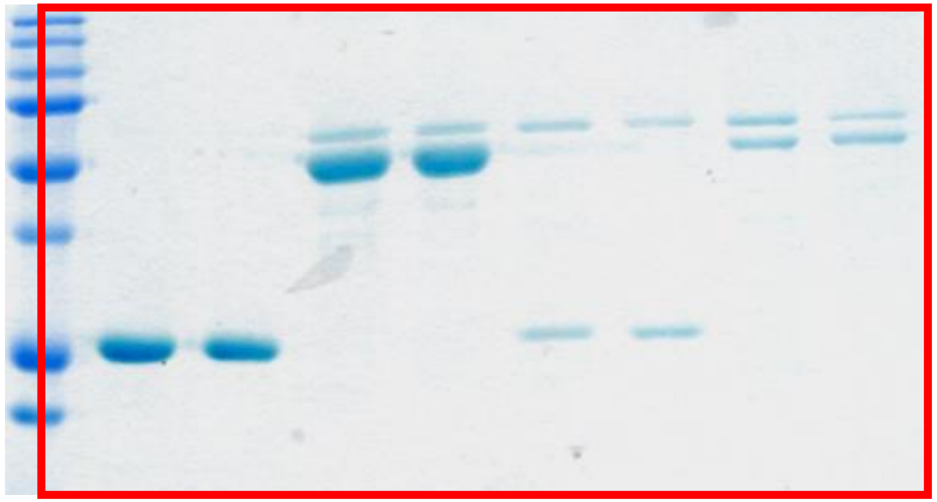

Figure 2-source data 3

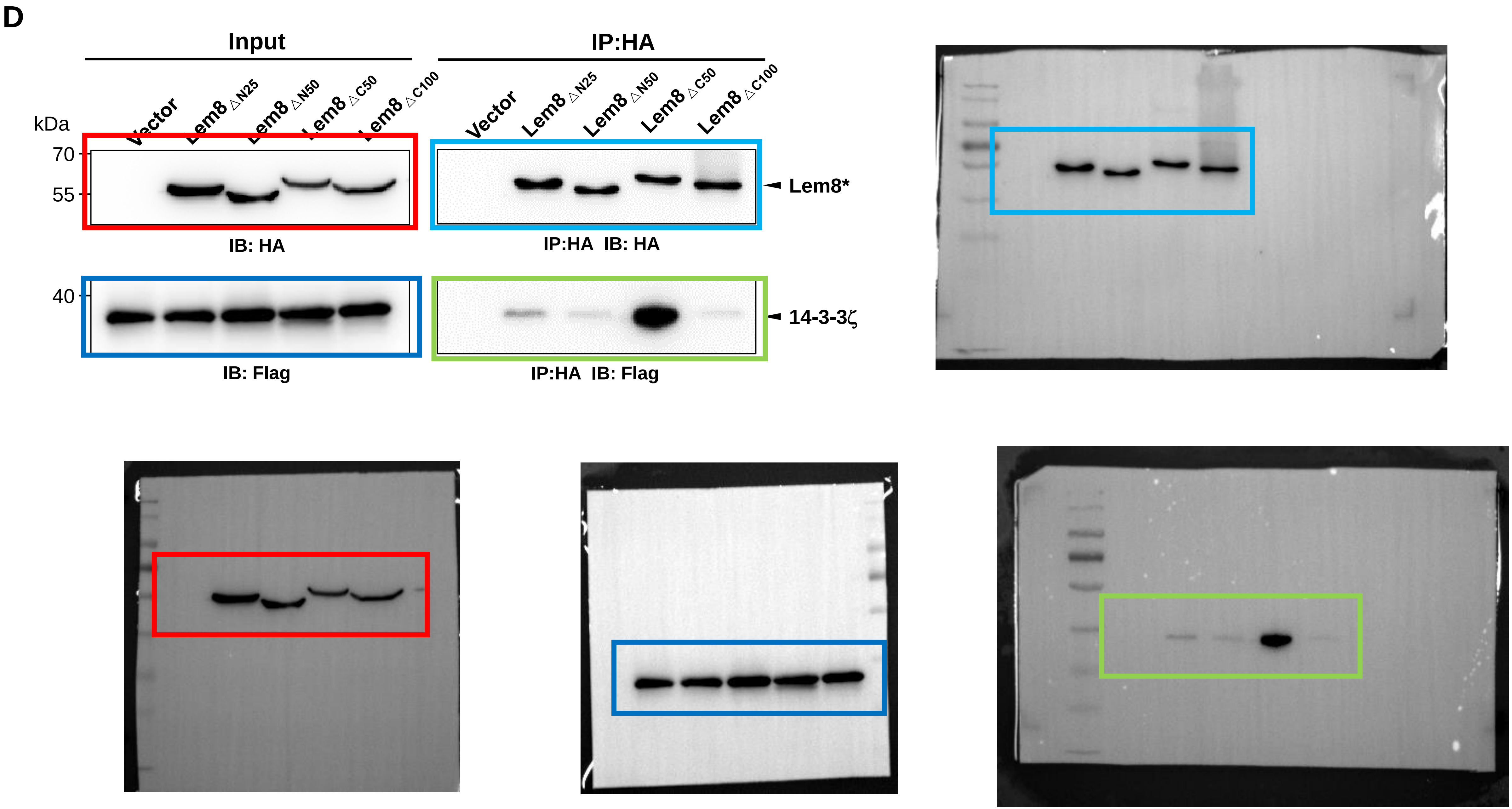

Figure 2-figure supplement 1-source data 1

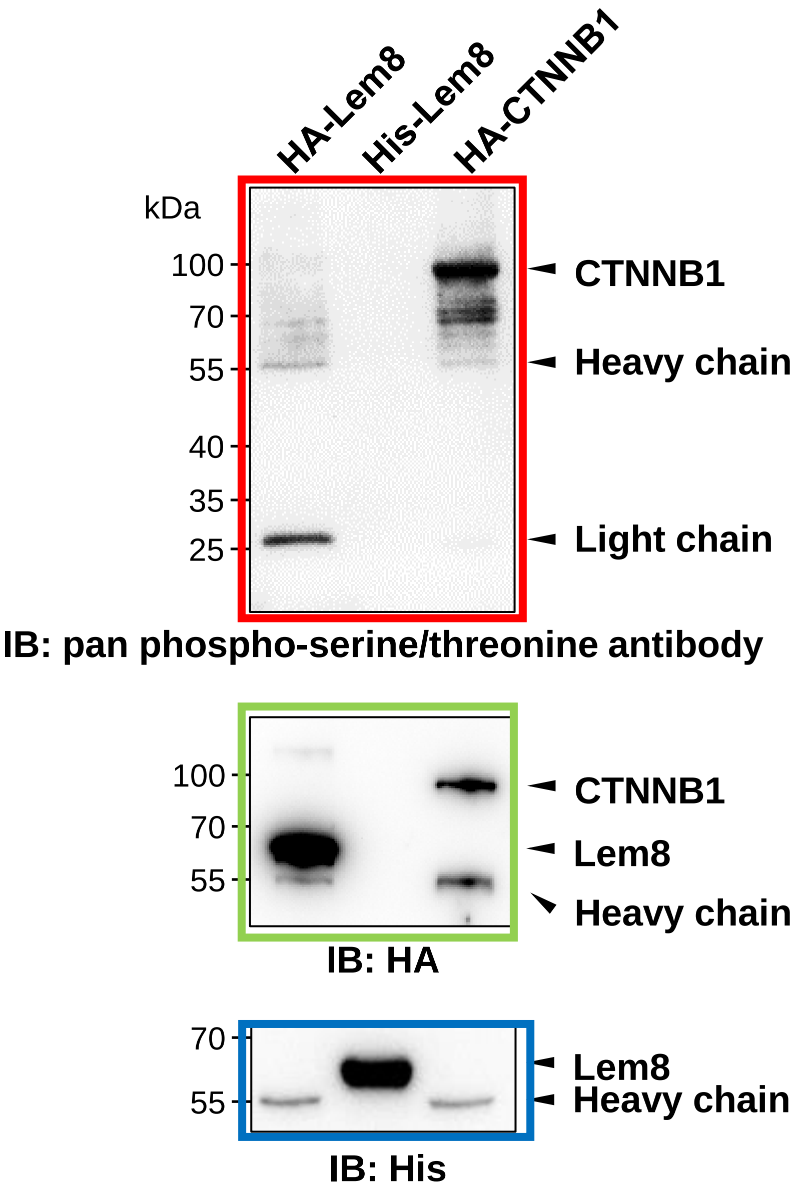

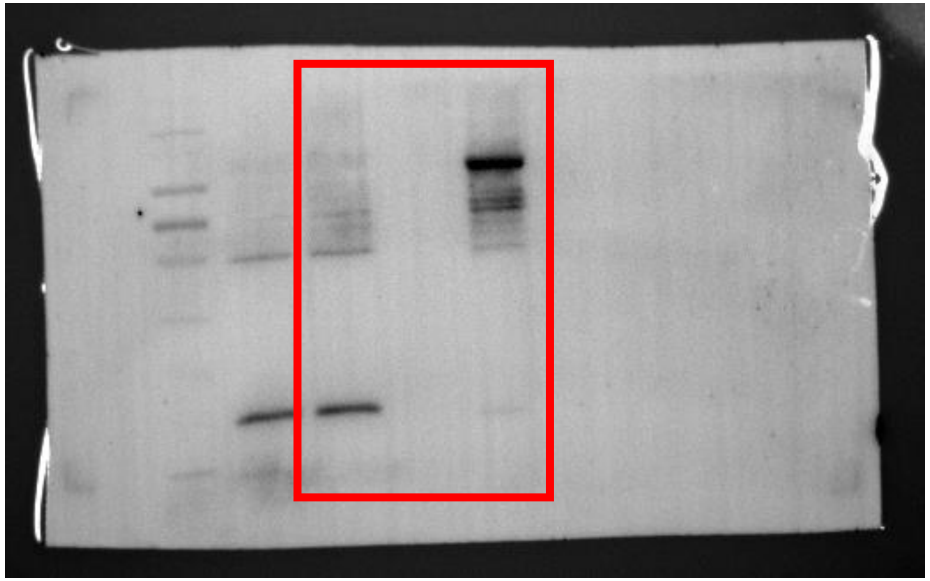

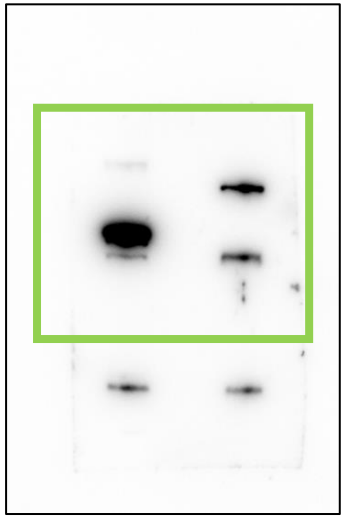

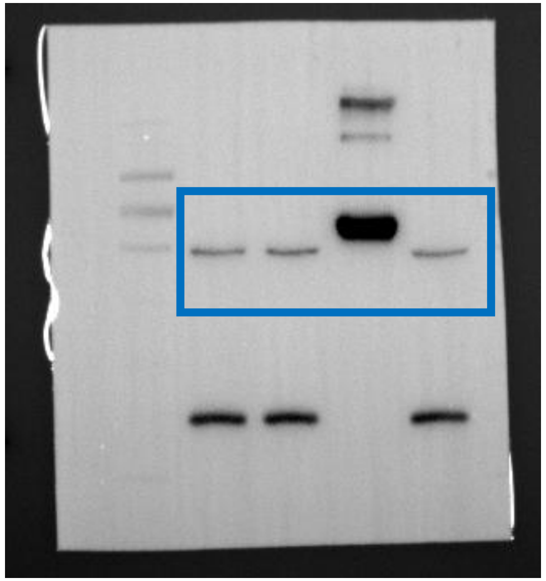

Figure 3-source data 1

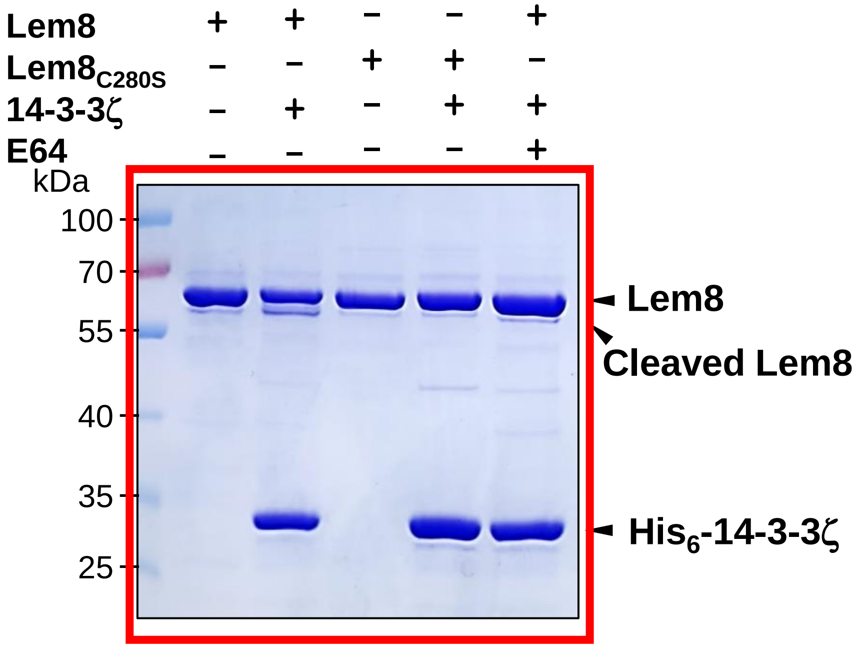

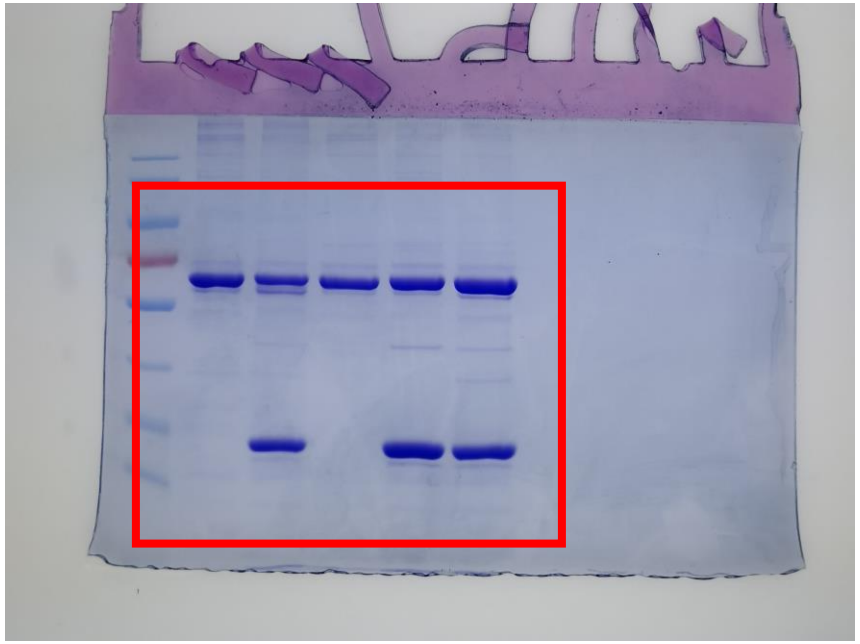

Figure 3-source data 2

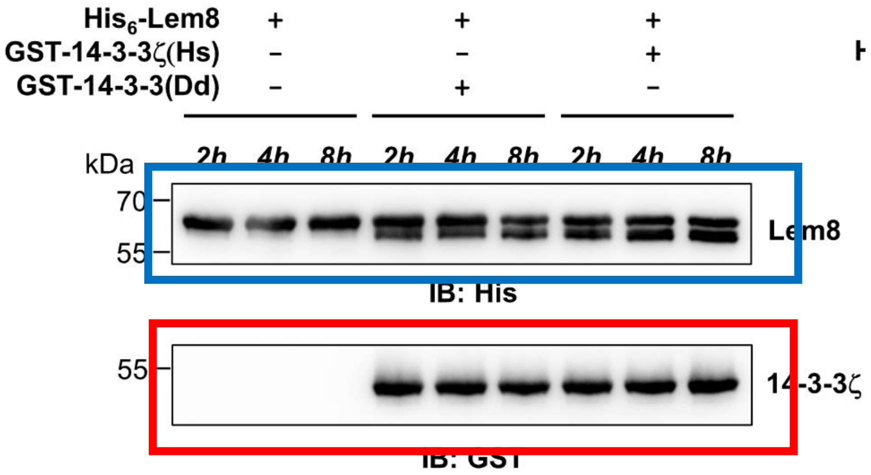

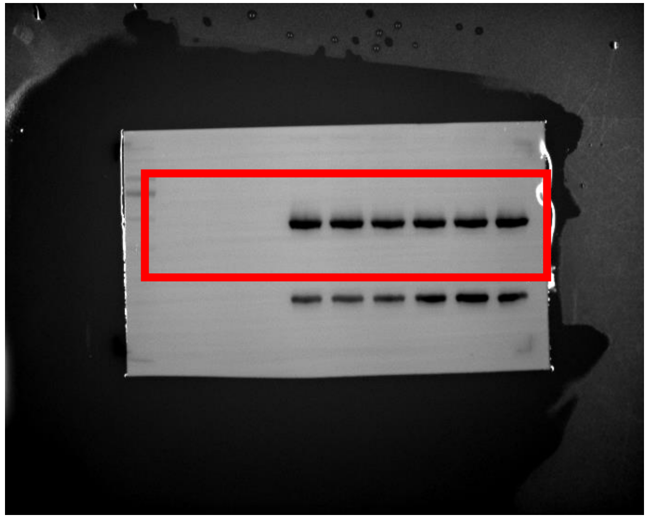

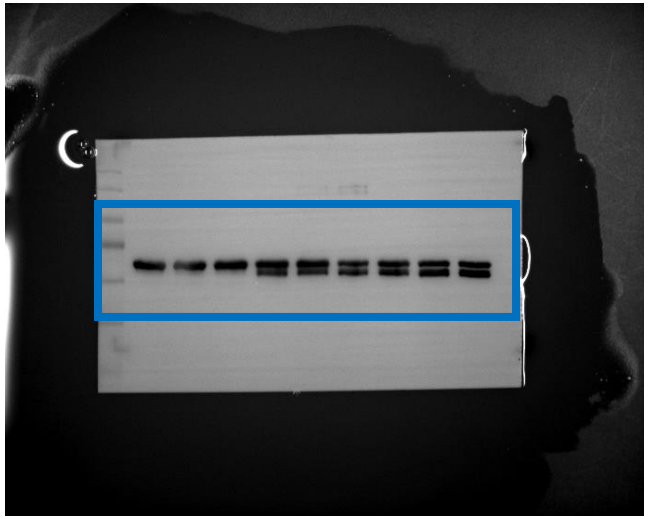

Figure 3-source data 3

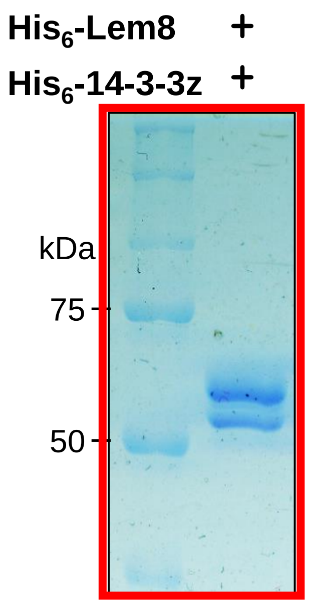

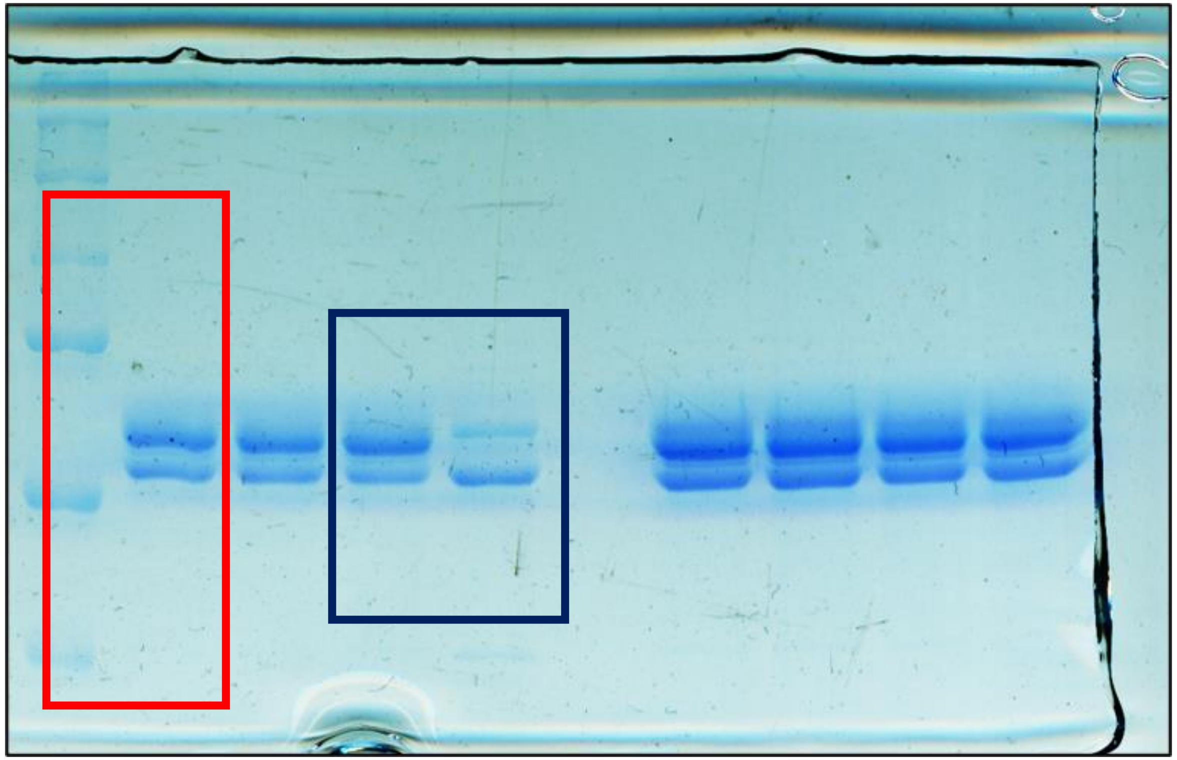

Figure 3-source data 4

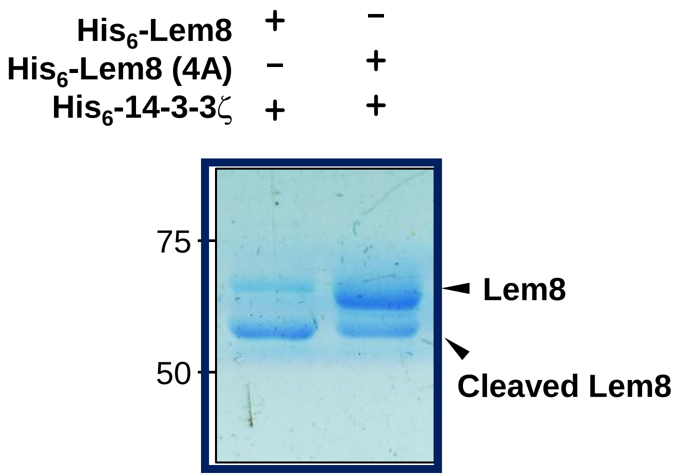

Figure 3-figure supplement 1-source data 1

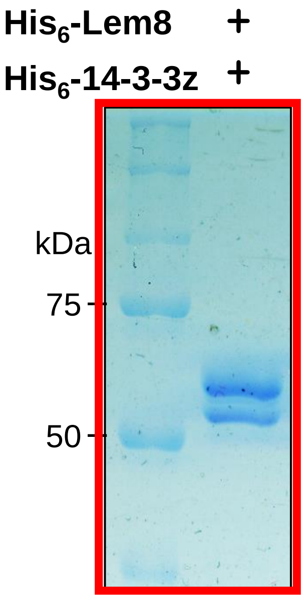

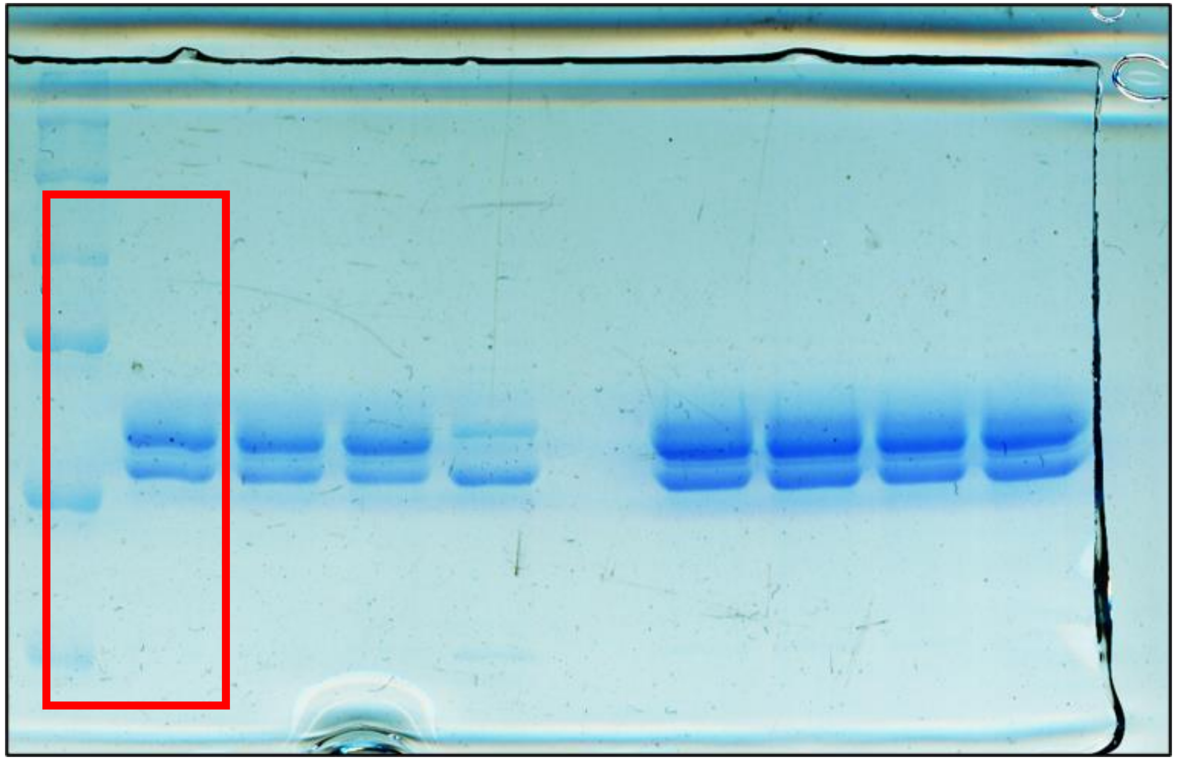

Figure 3-figure supplement 1-source data 2

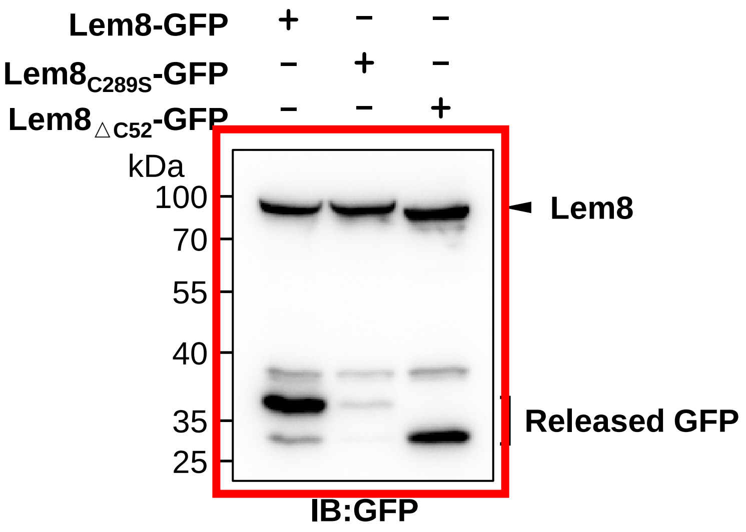

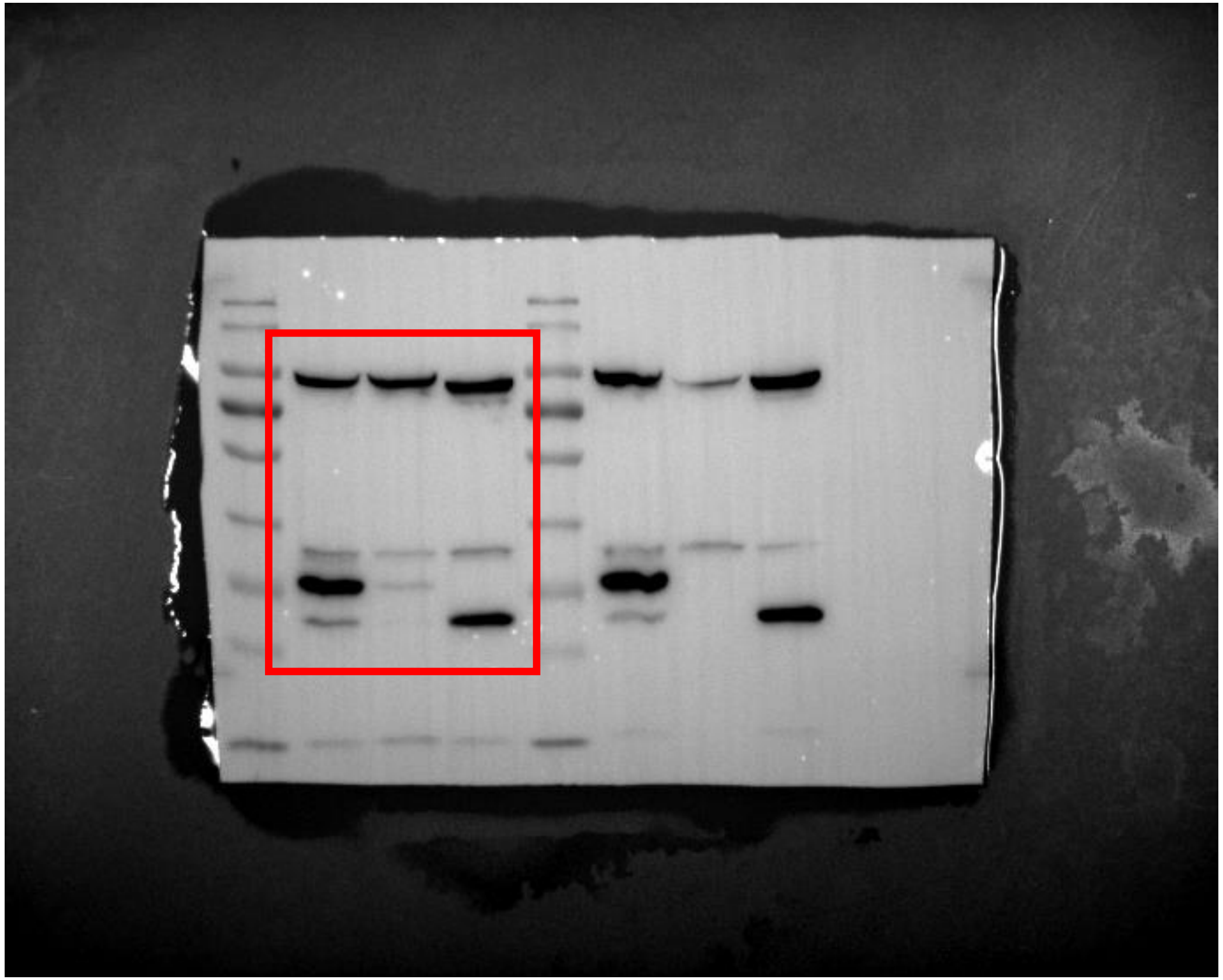

Figure 3-figure supplement 1-source data 3

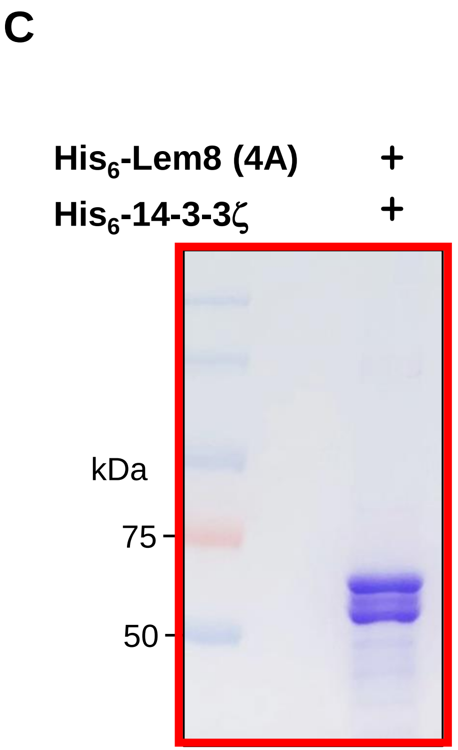

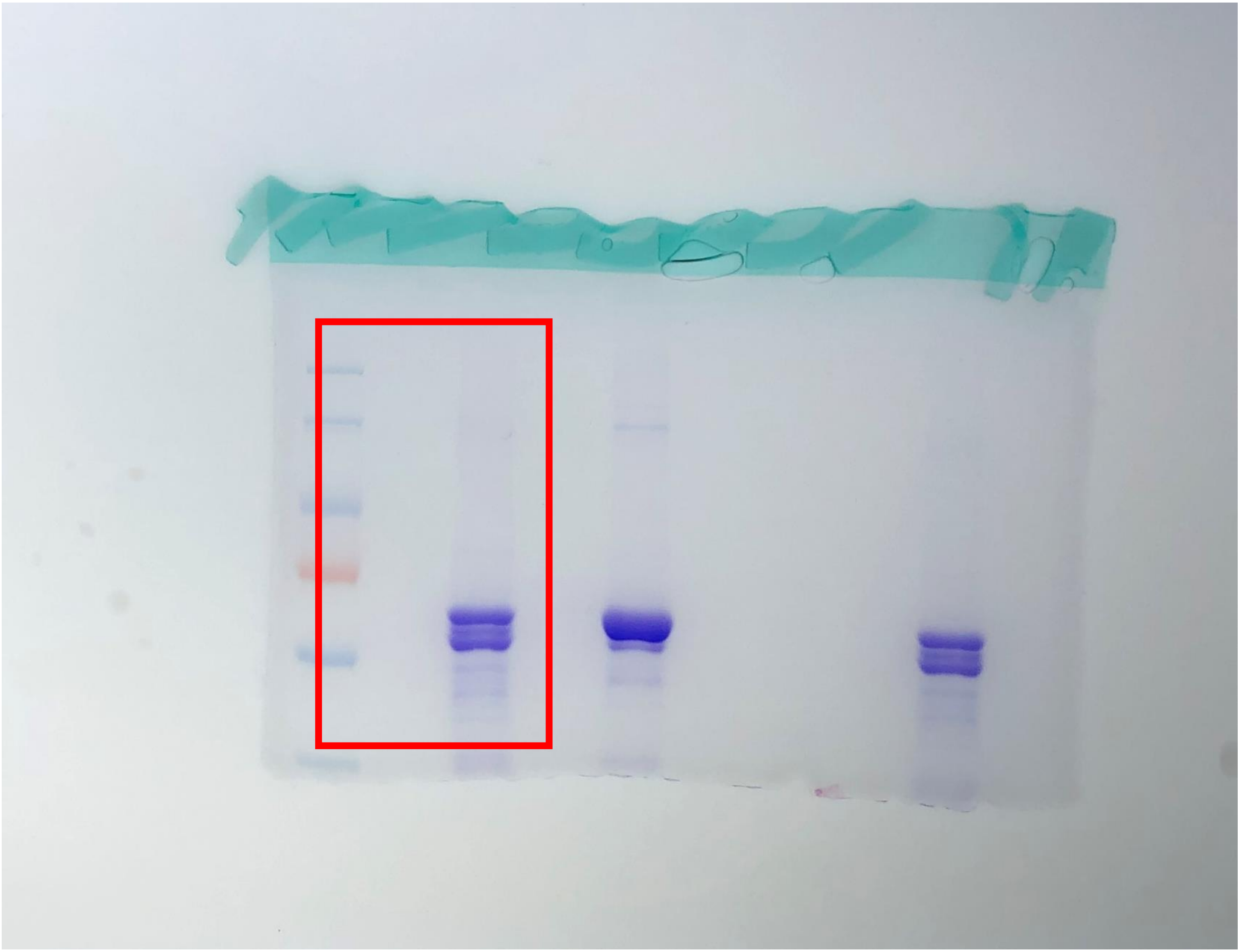

Figure 4-source data 1

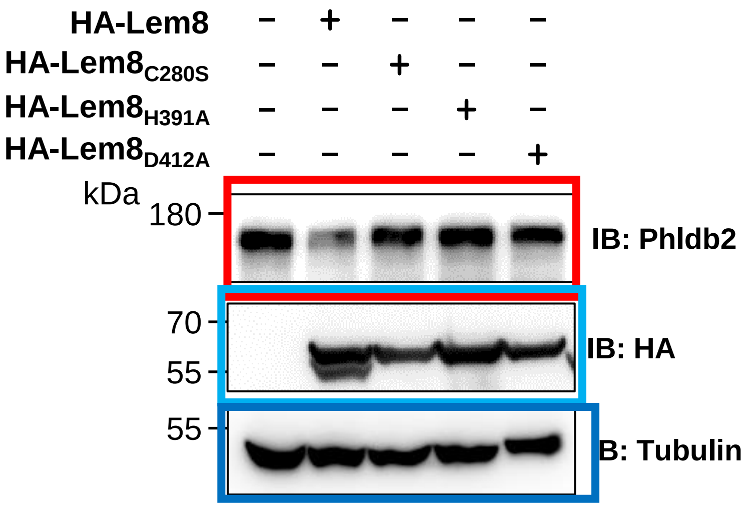

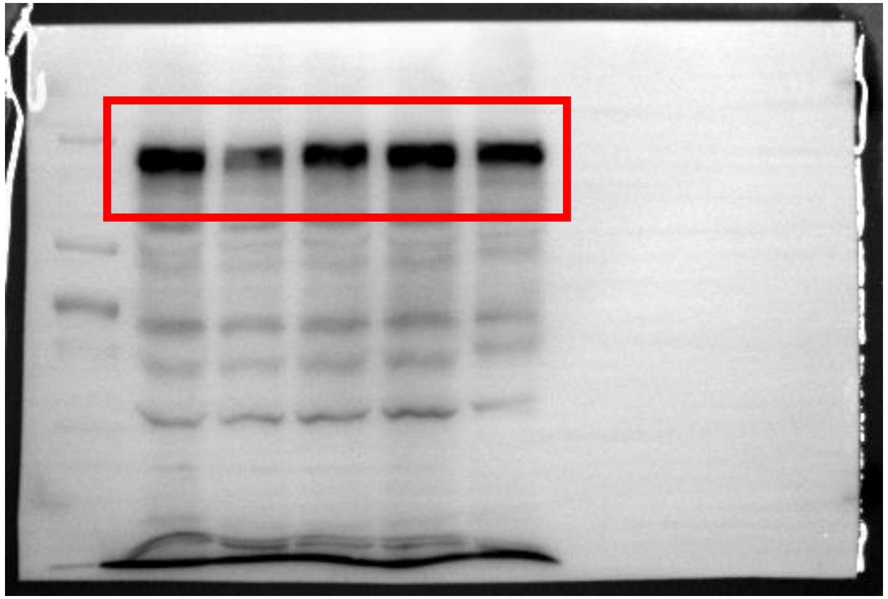

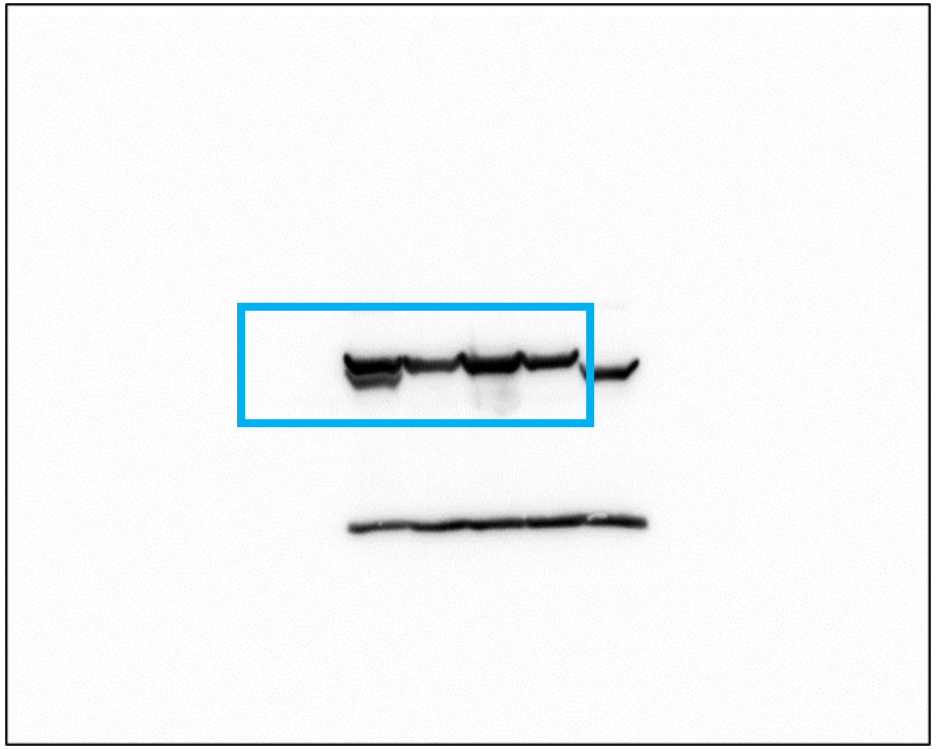

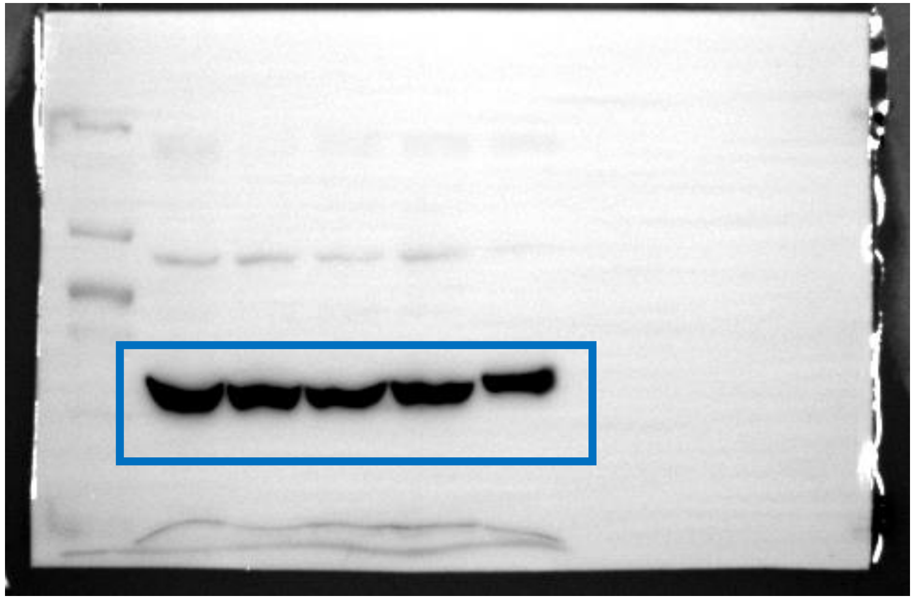

Figure 4-source data 2

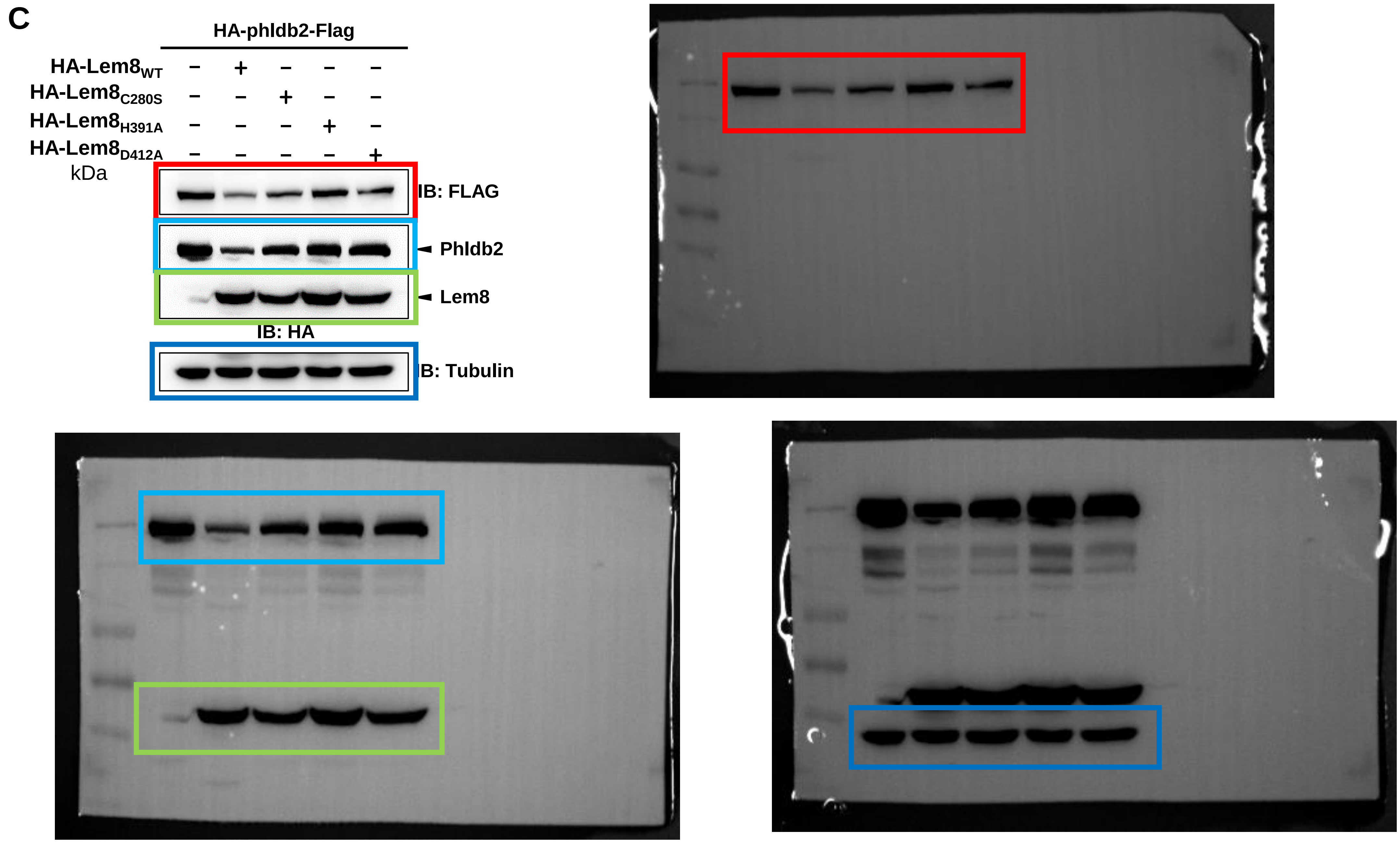

Figure 4-source data 4

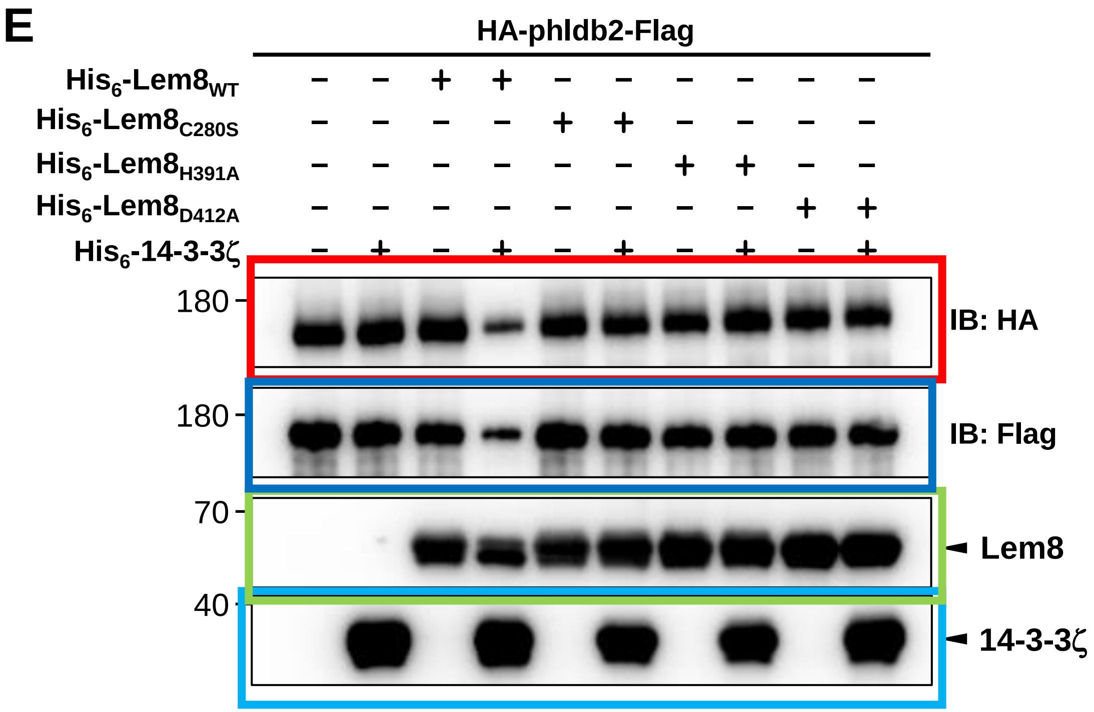

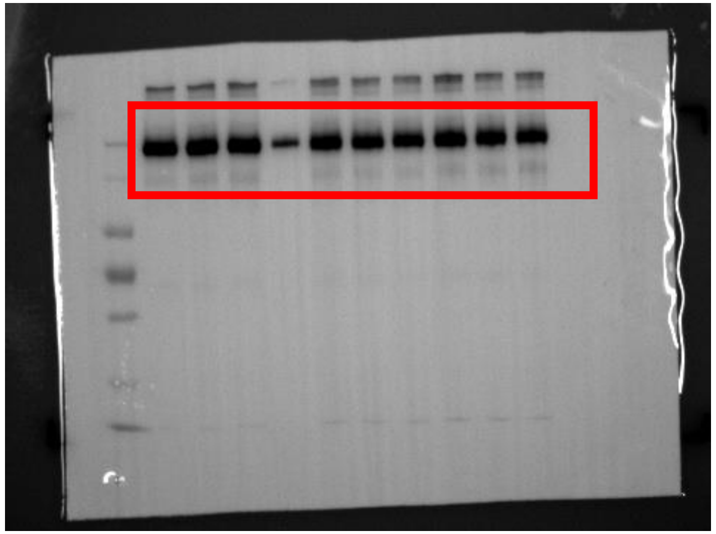

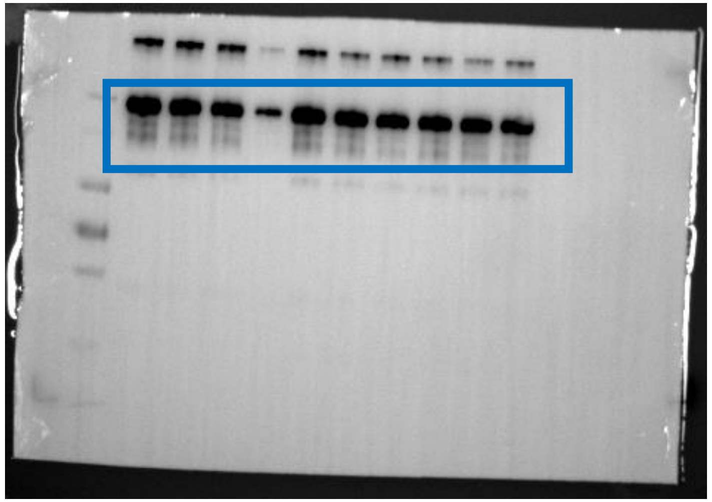

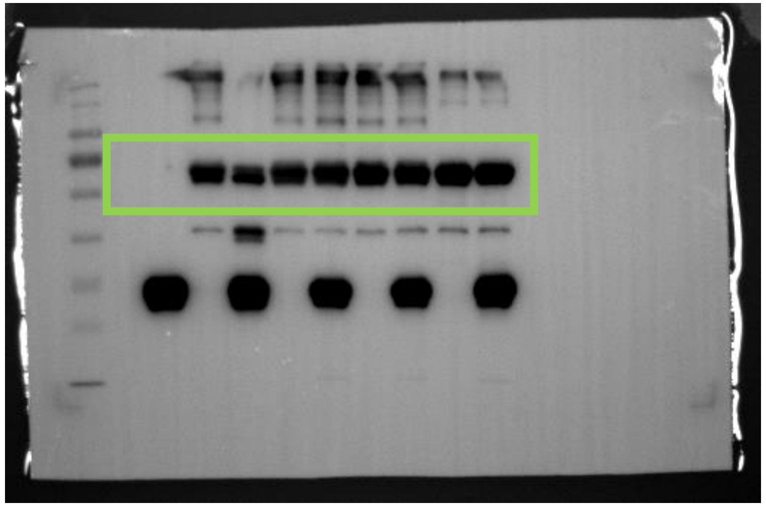

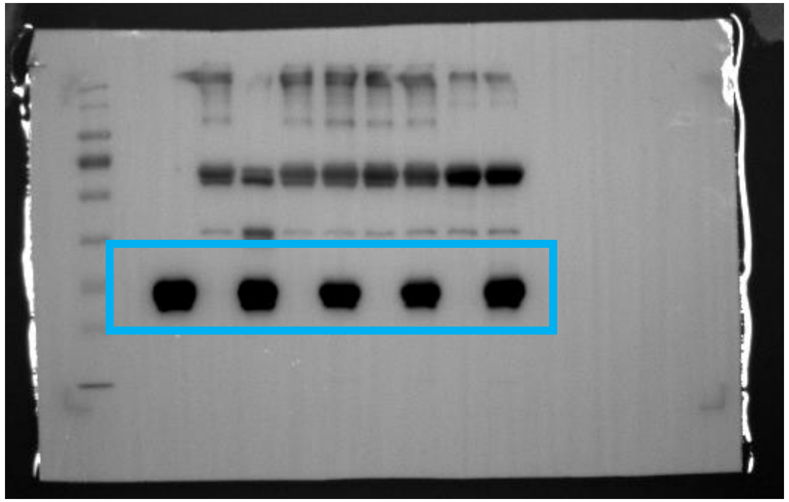

Figure 4-figure supplement 1-source data 1

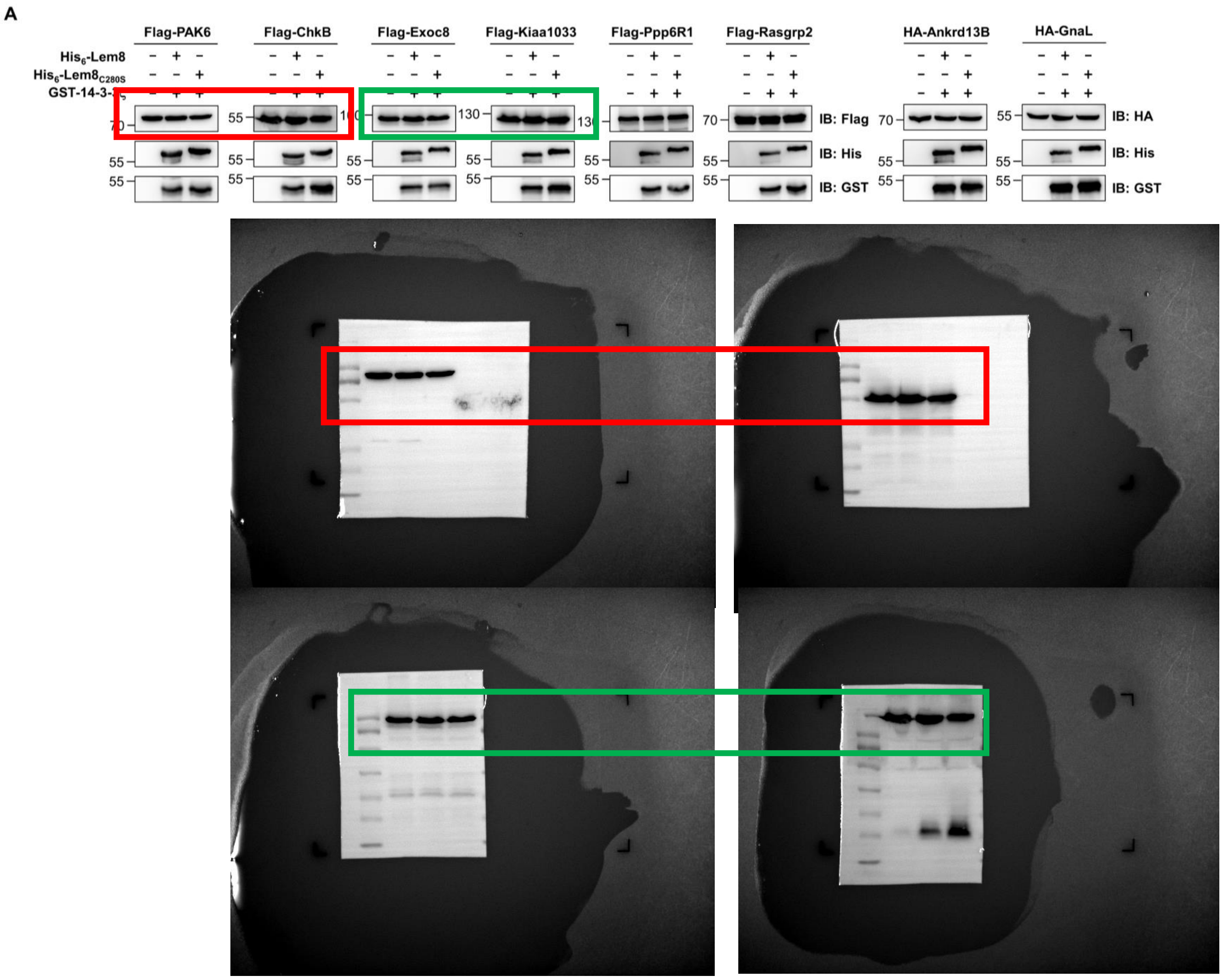

Figure 4-figure supplement 1-source data 1

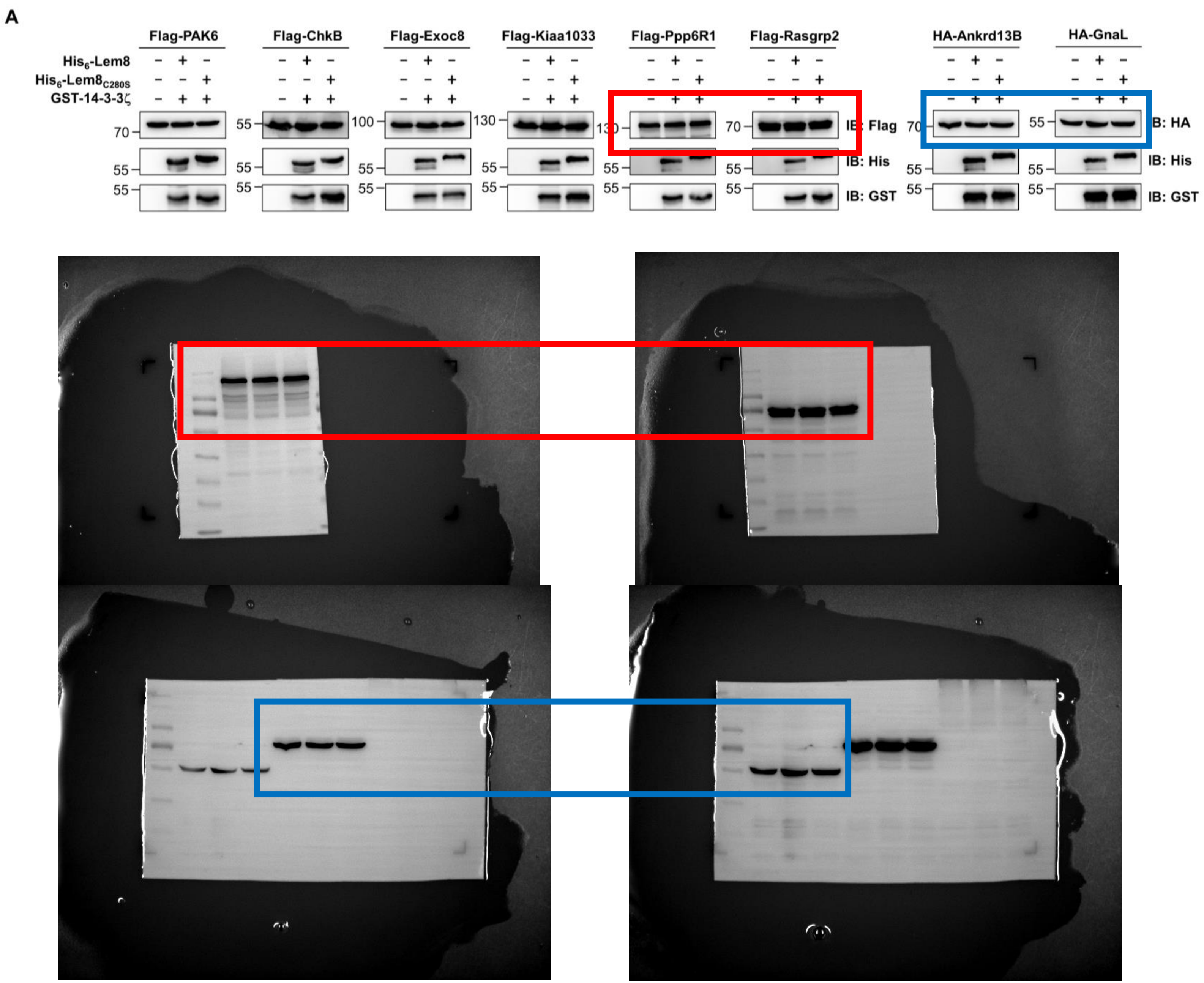

Figure 4-figure supplement 1-source data 1

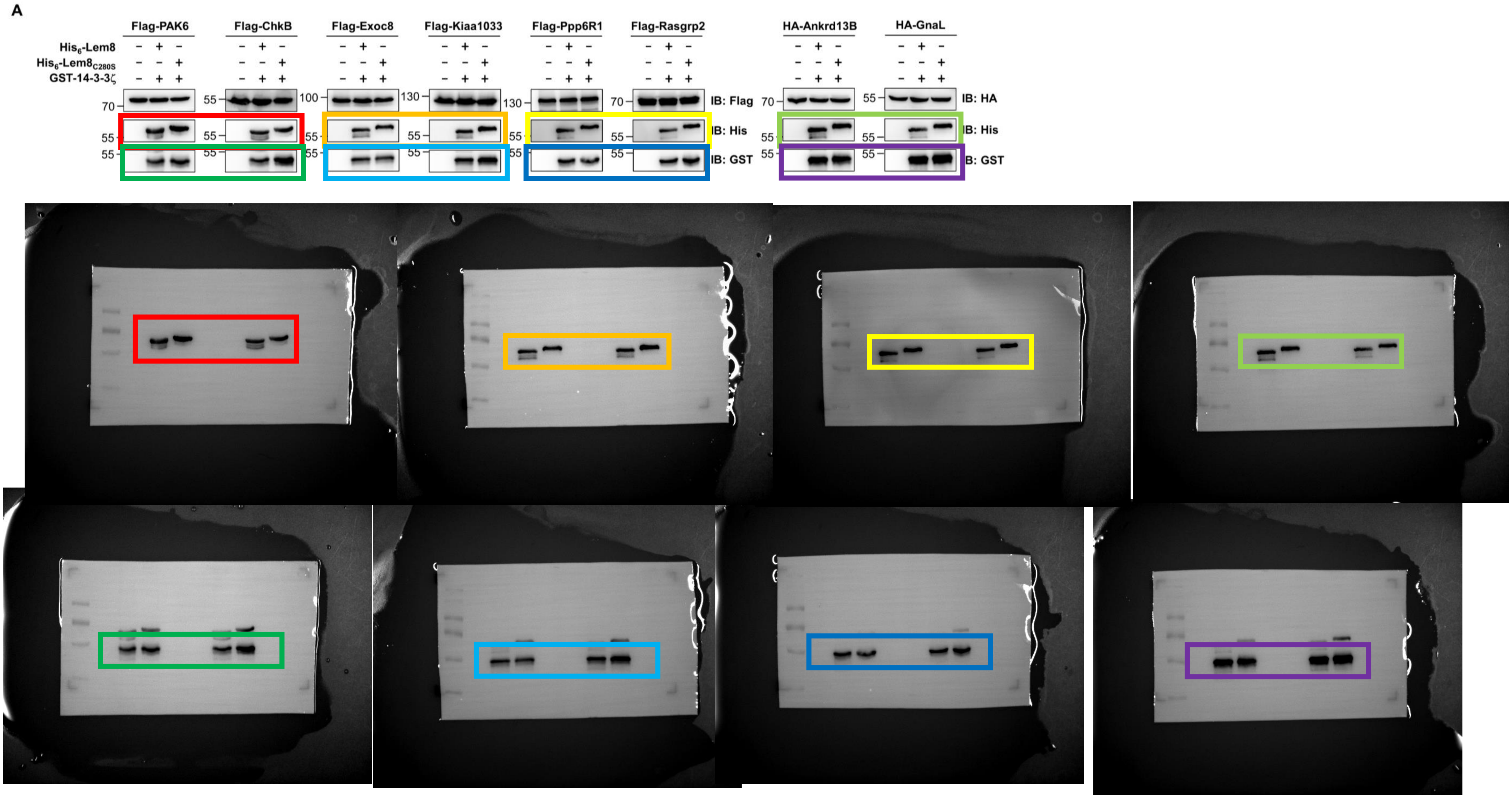

Figure 4-figure supplement 1-source data 3

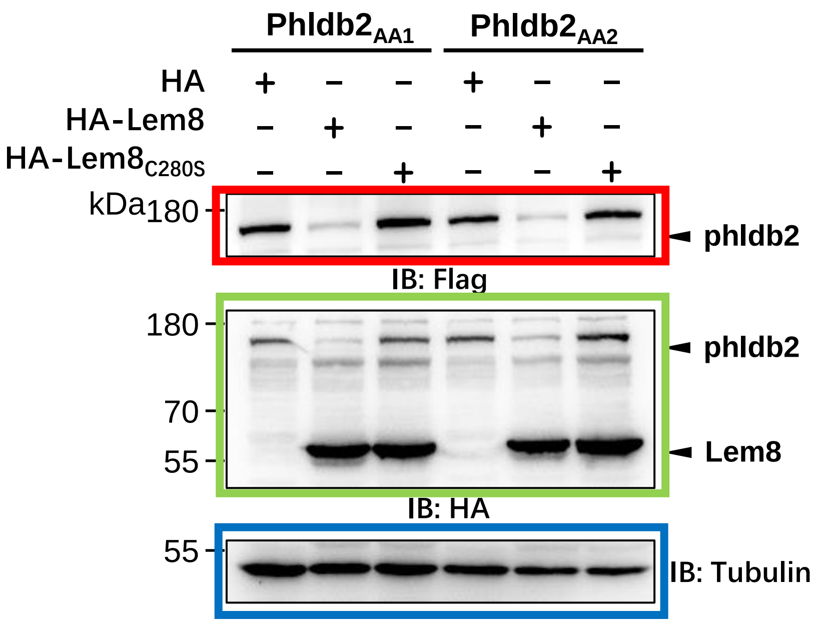

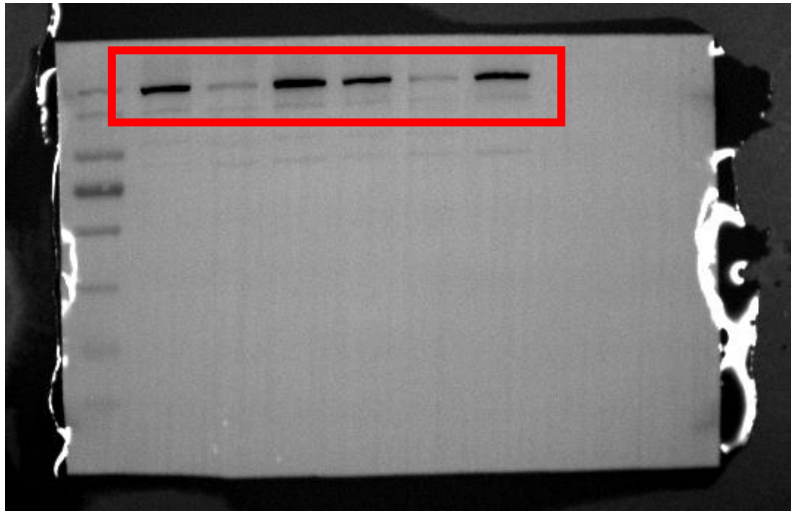

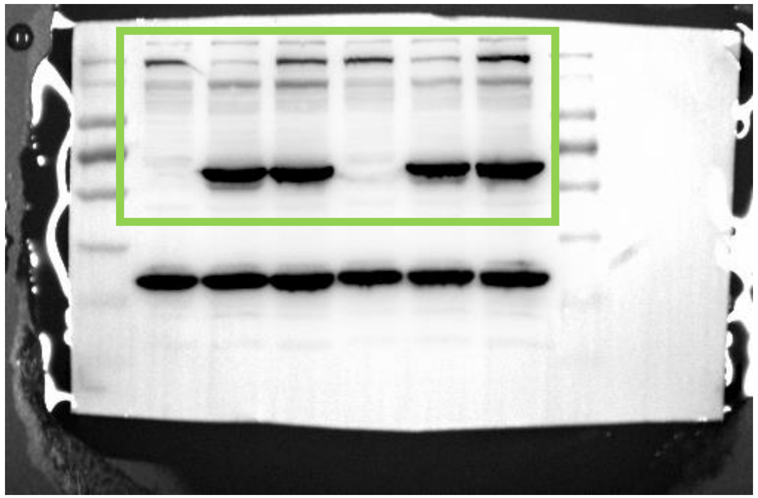

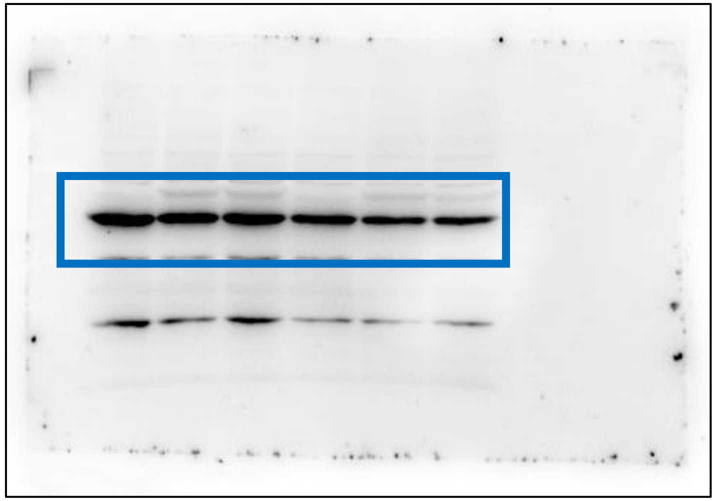

Figure 4-figure supplement 1-source data 4

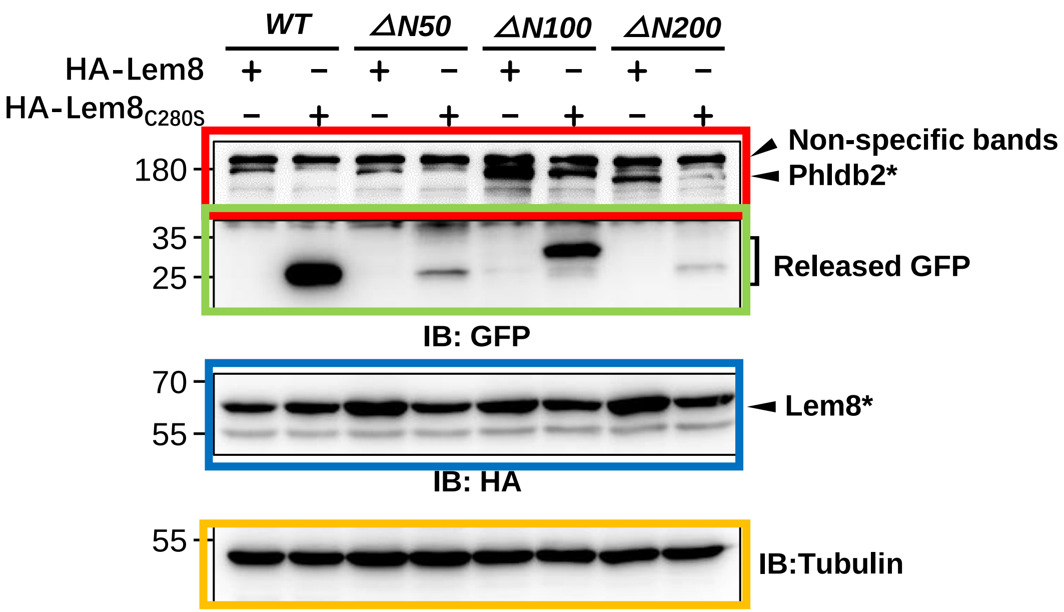

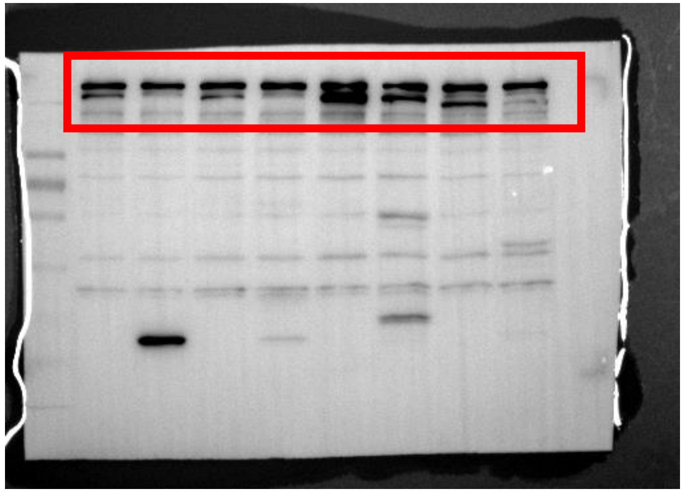

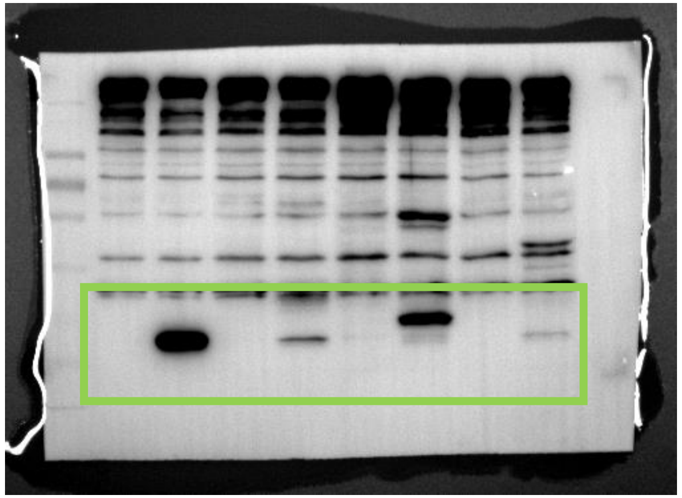

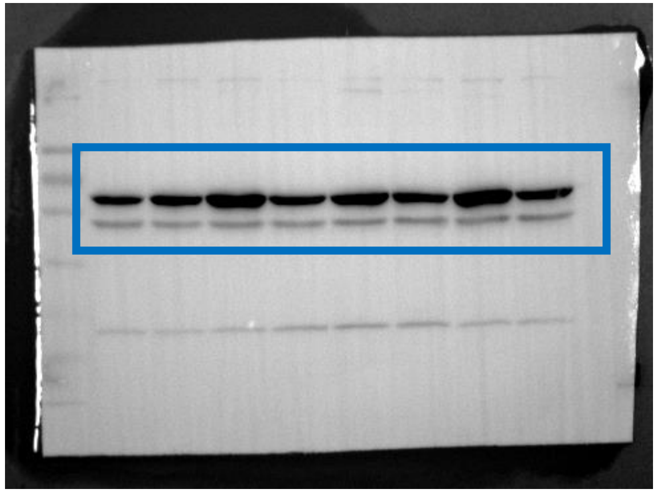

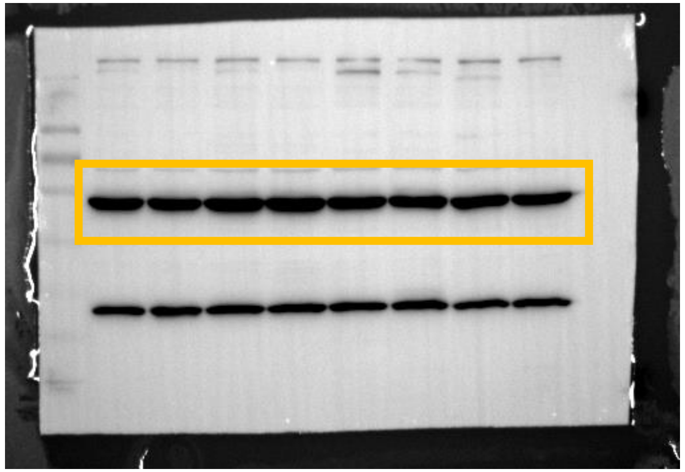

Figure 4-figure supplement 1-source data 4

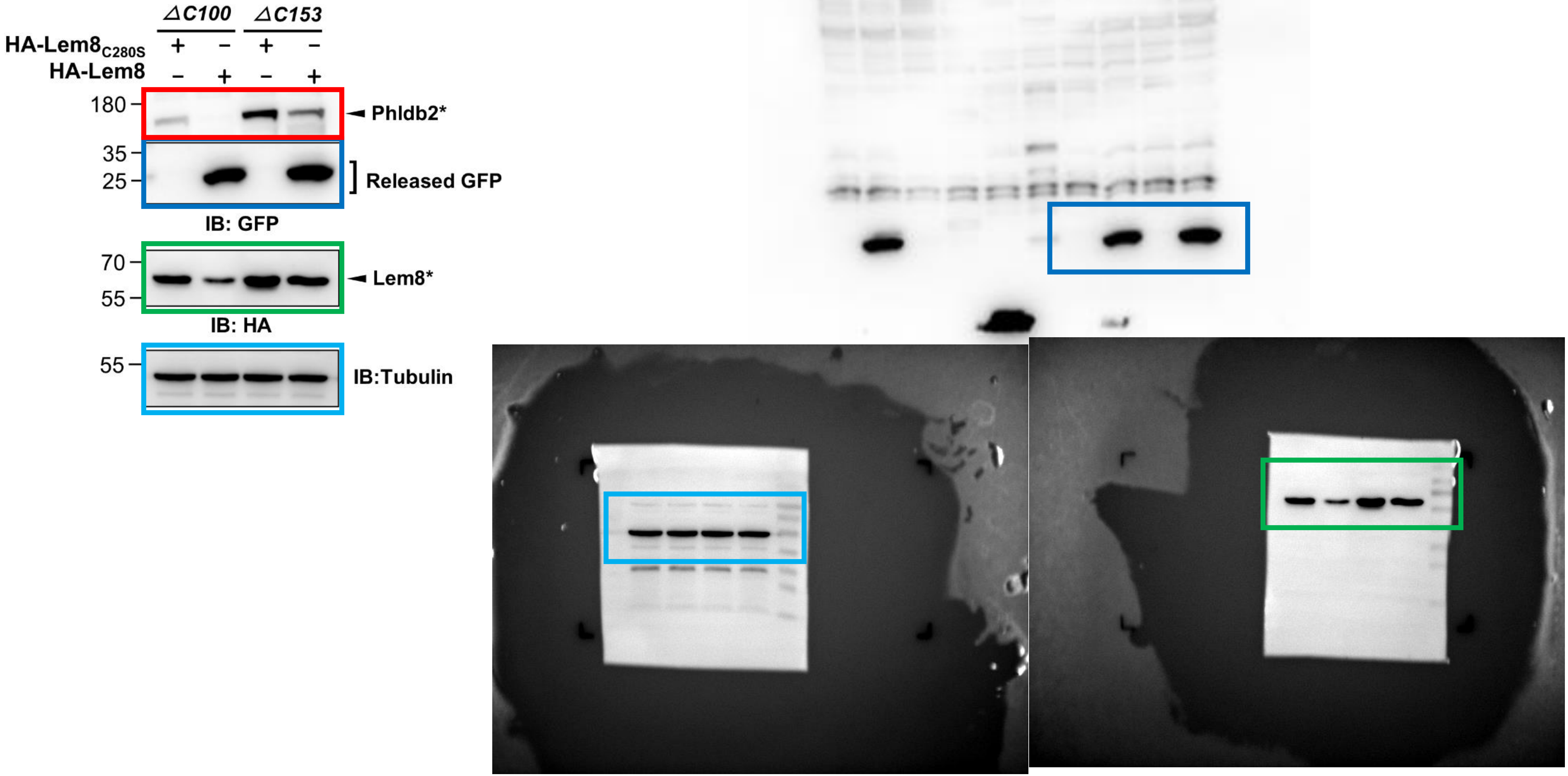

Figure 4-figure supplement 1-source data 5

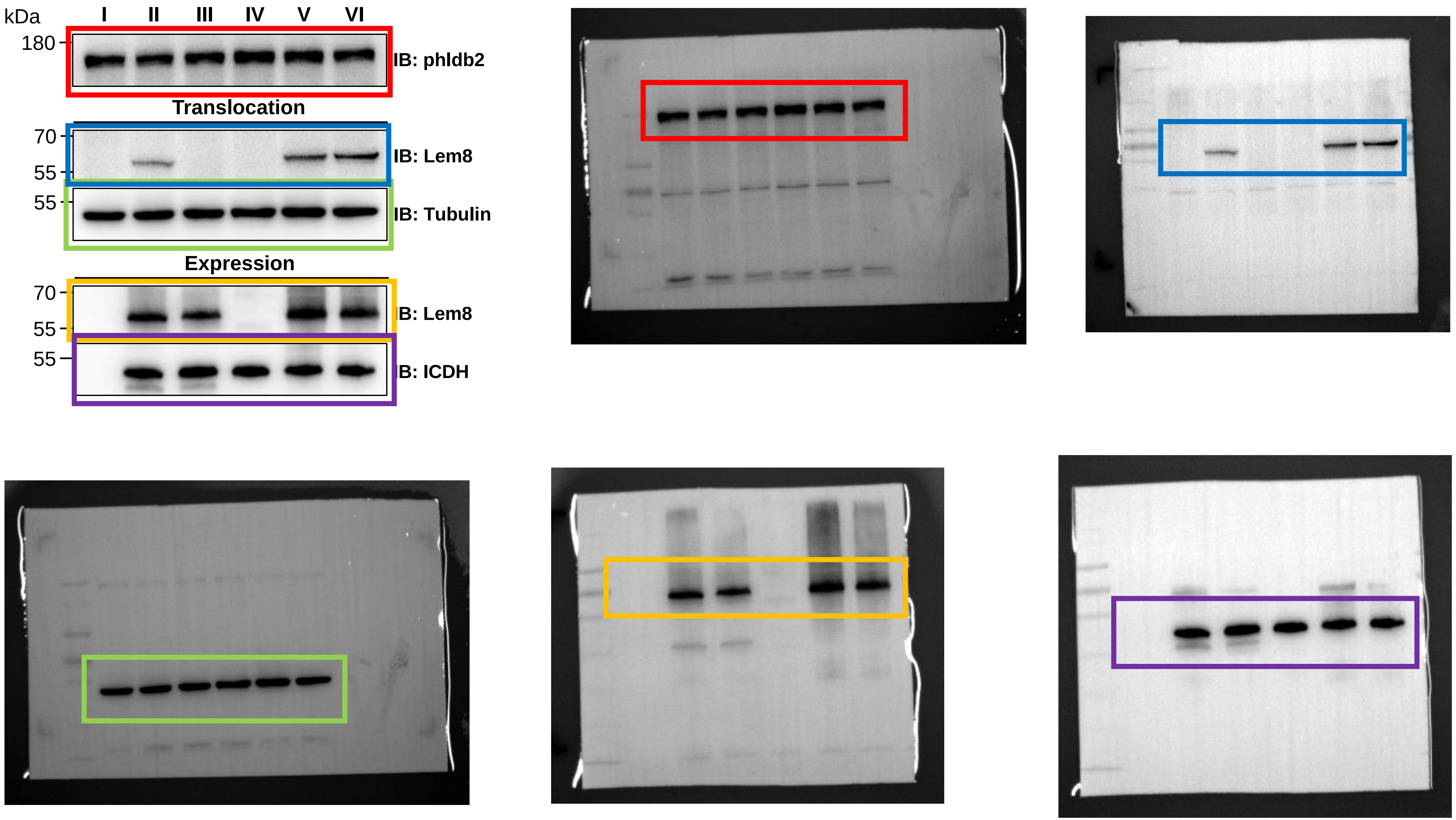

Figure 5-source data 1

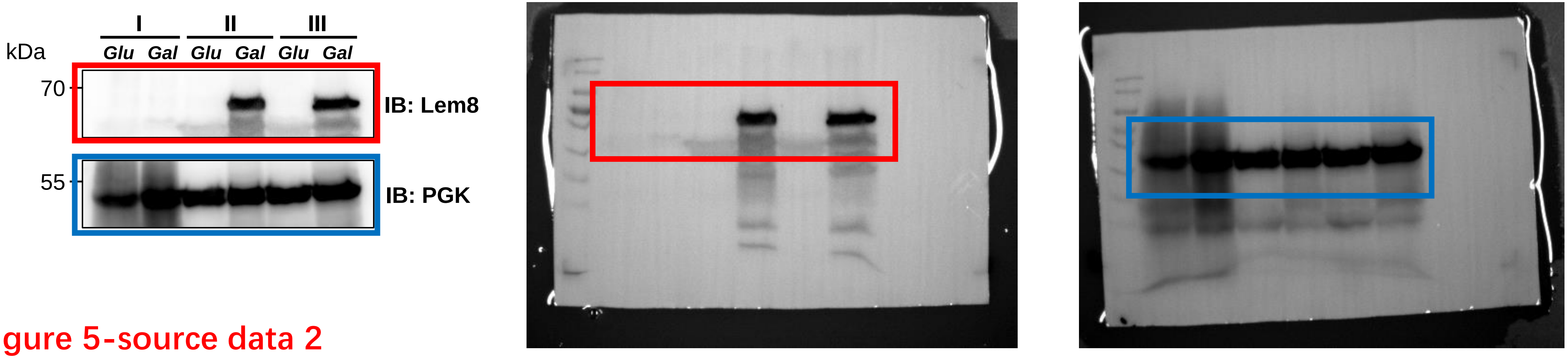

Figure 5-source data 2

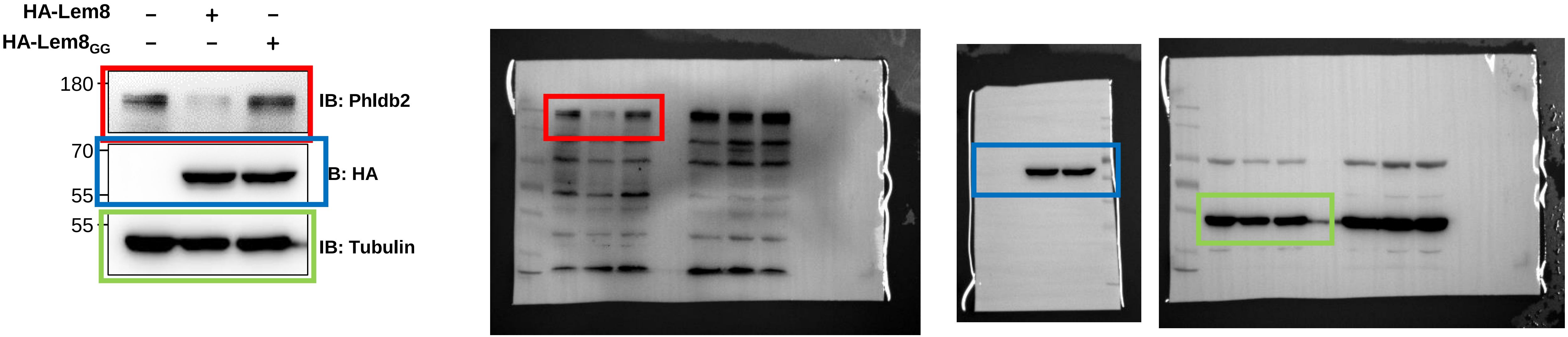

Figure 5-source data 3

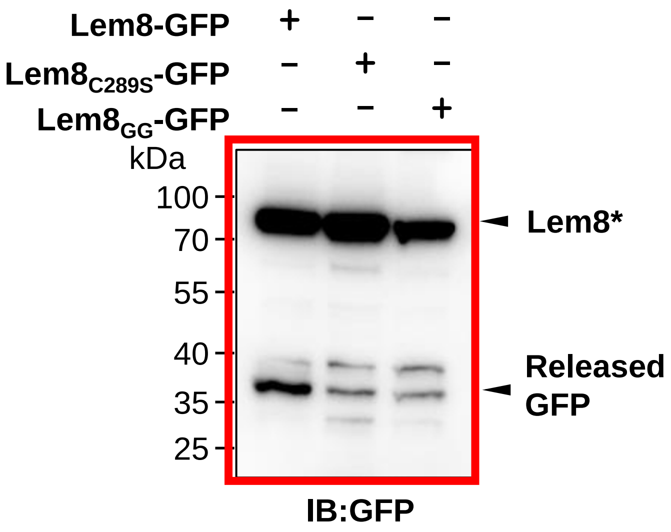

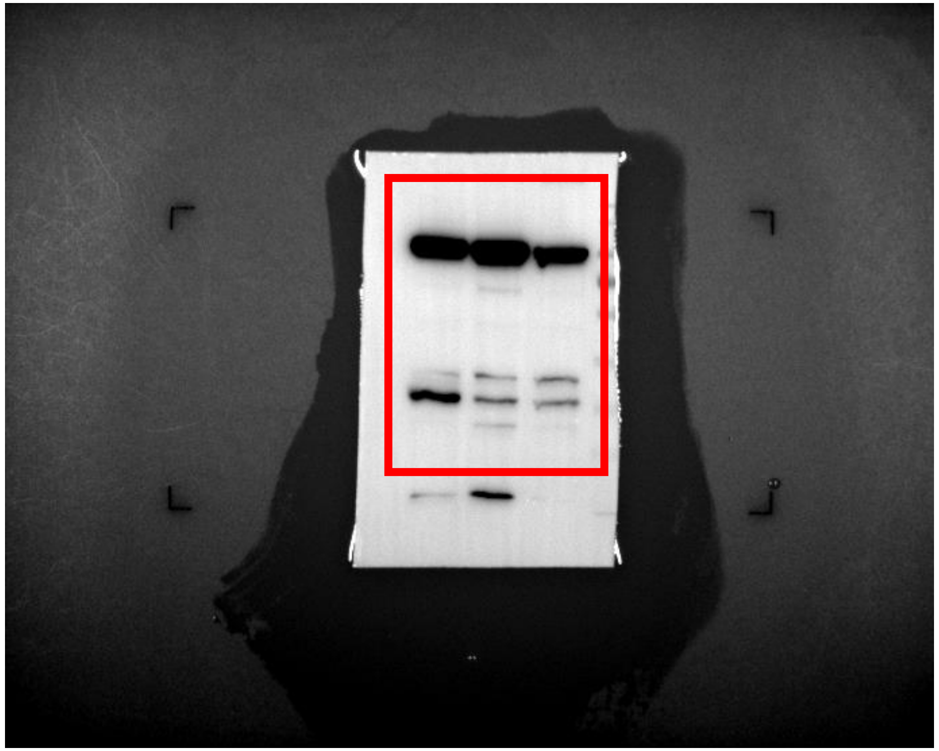

Figure 5-source data 4

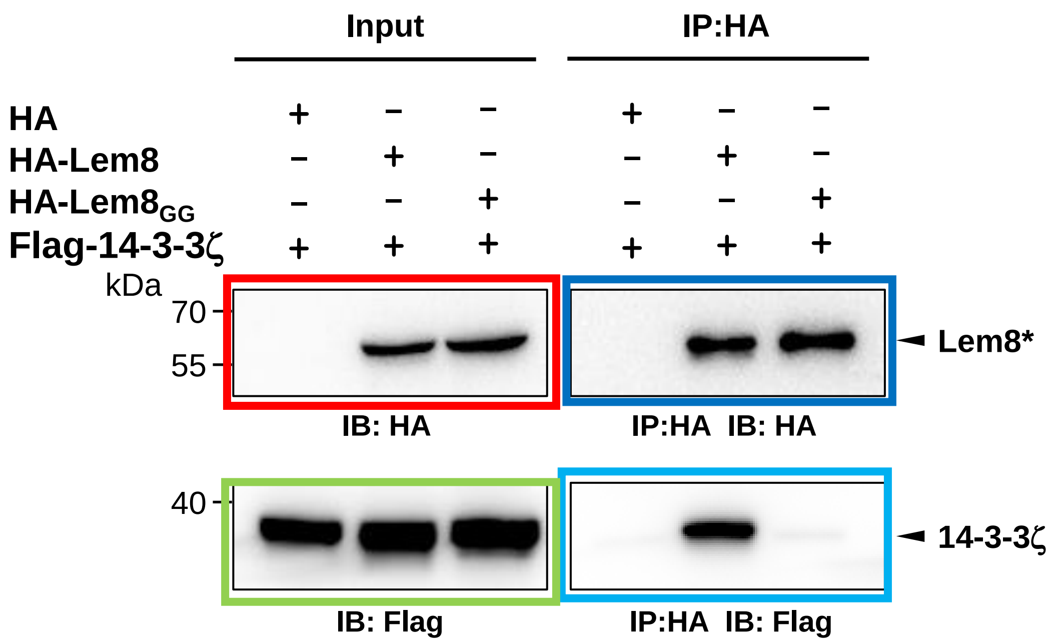

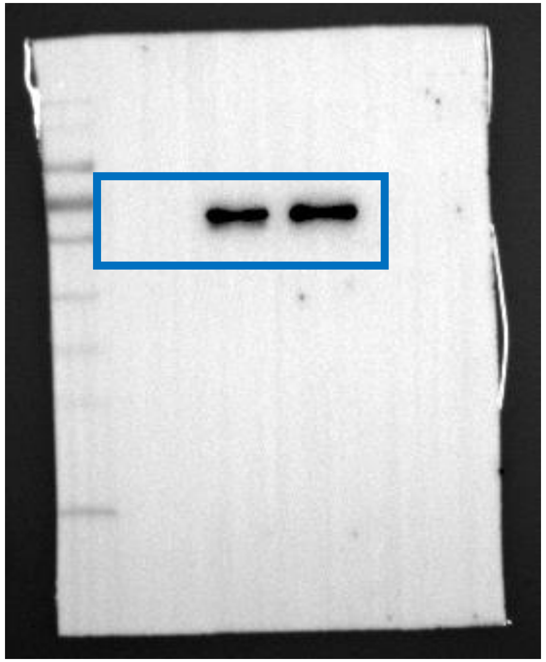

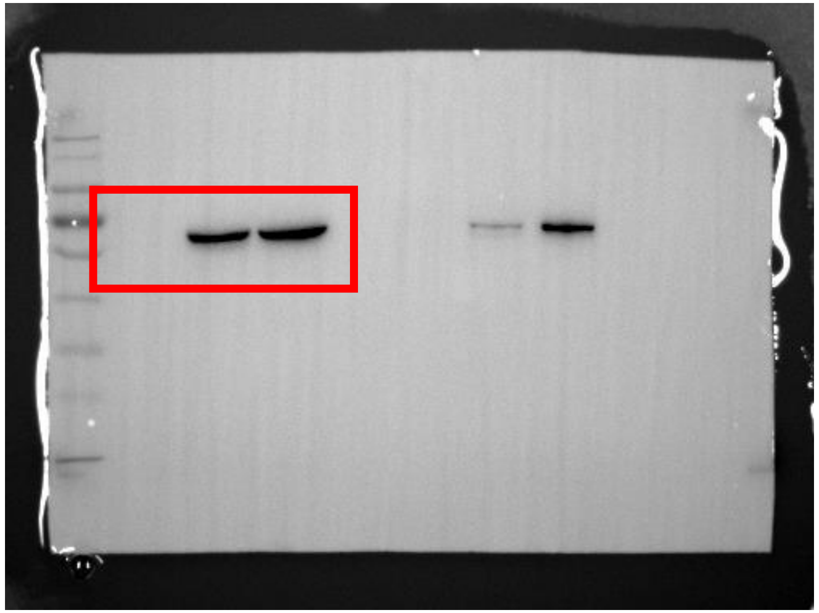

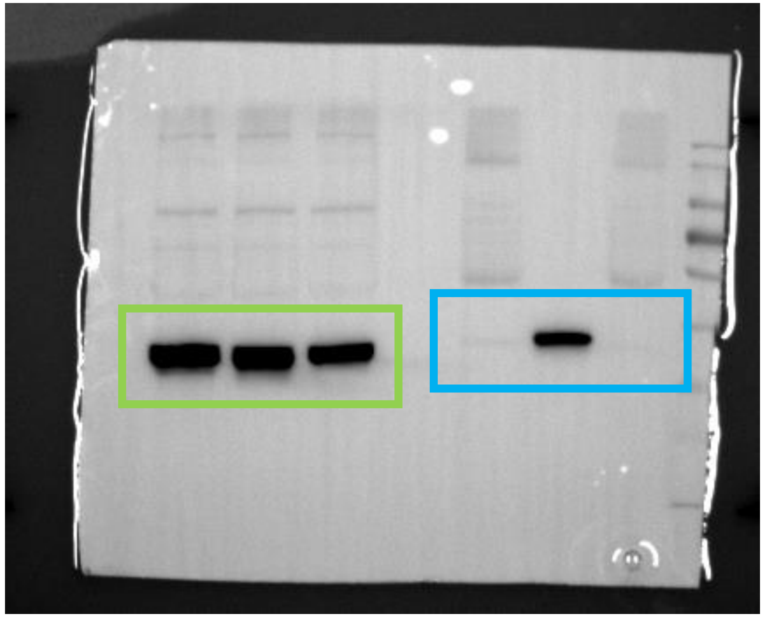

Figure 6-source data 1

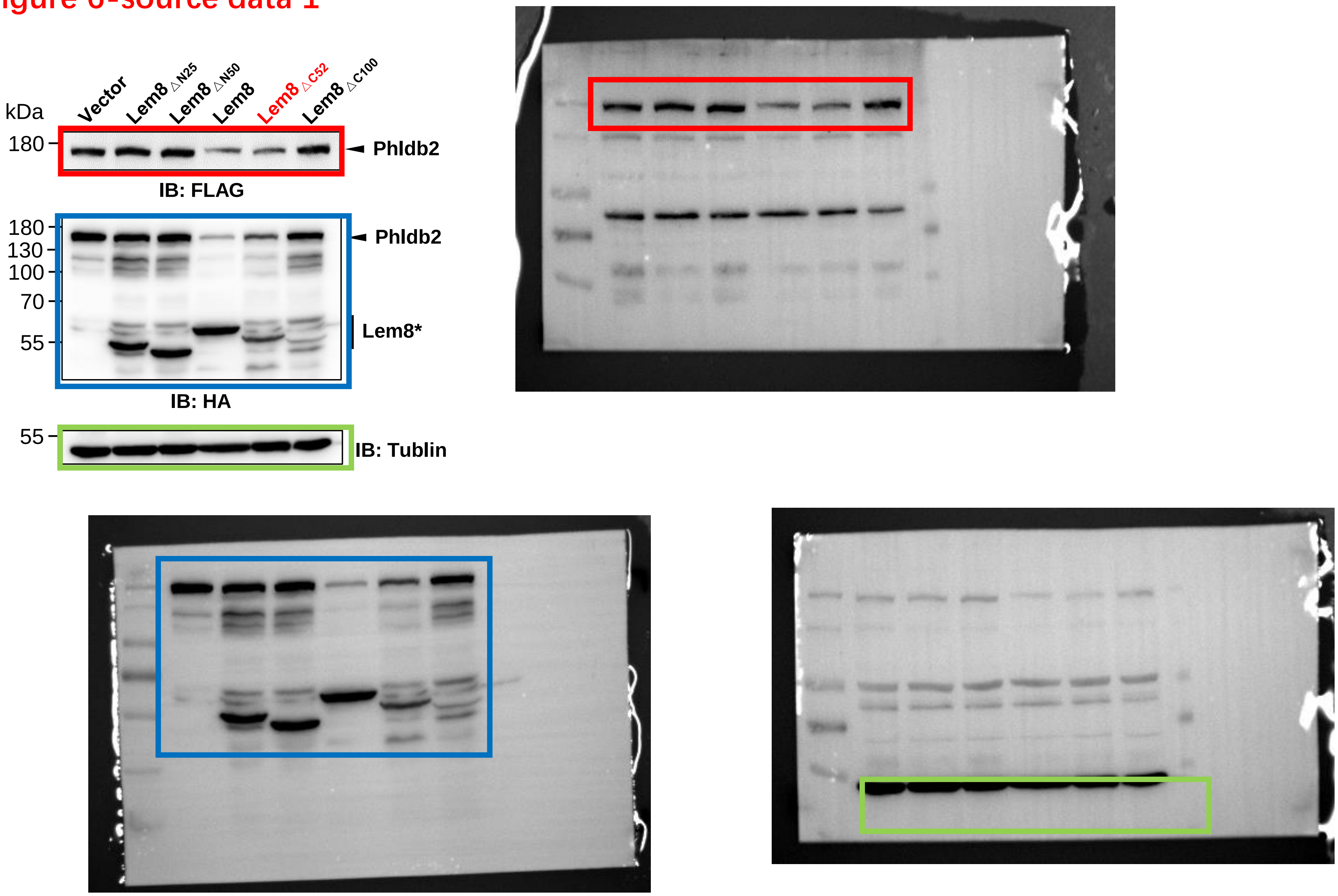

Figure 6-source data 3

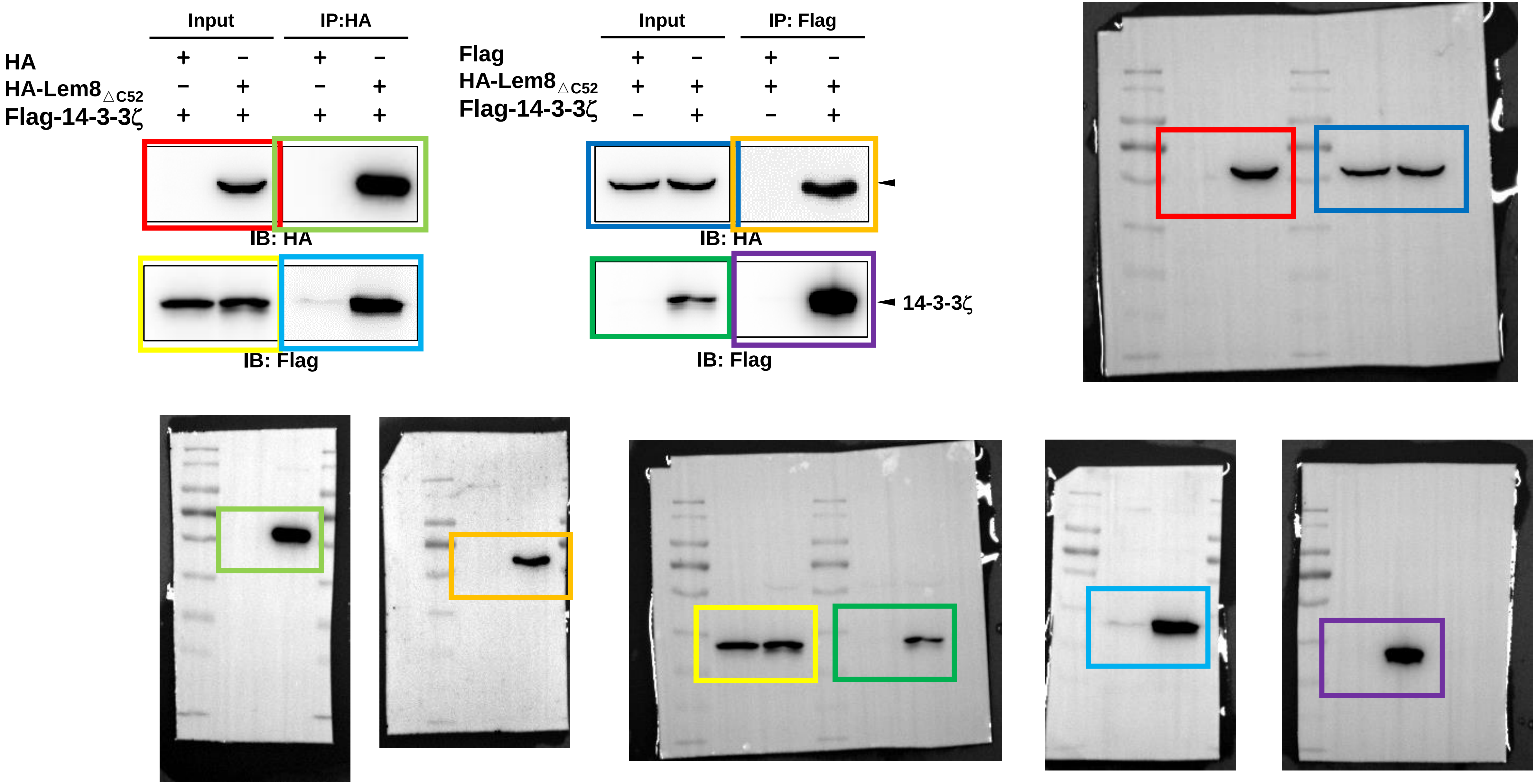

Figure 6-source data 4

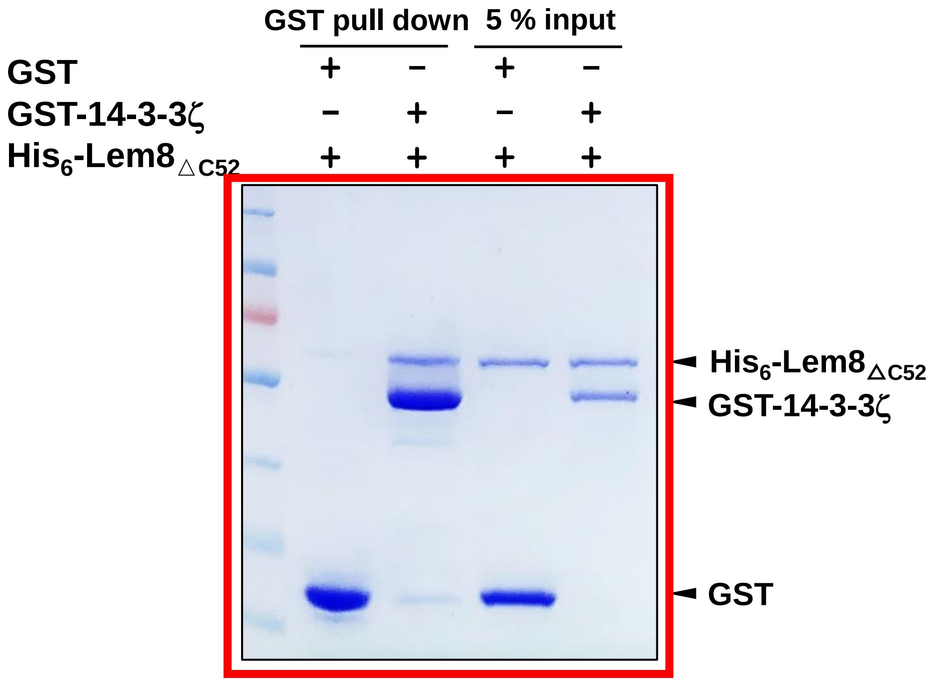

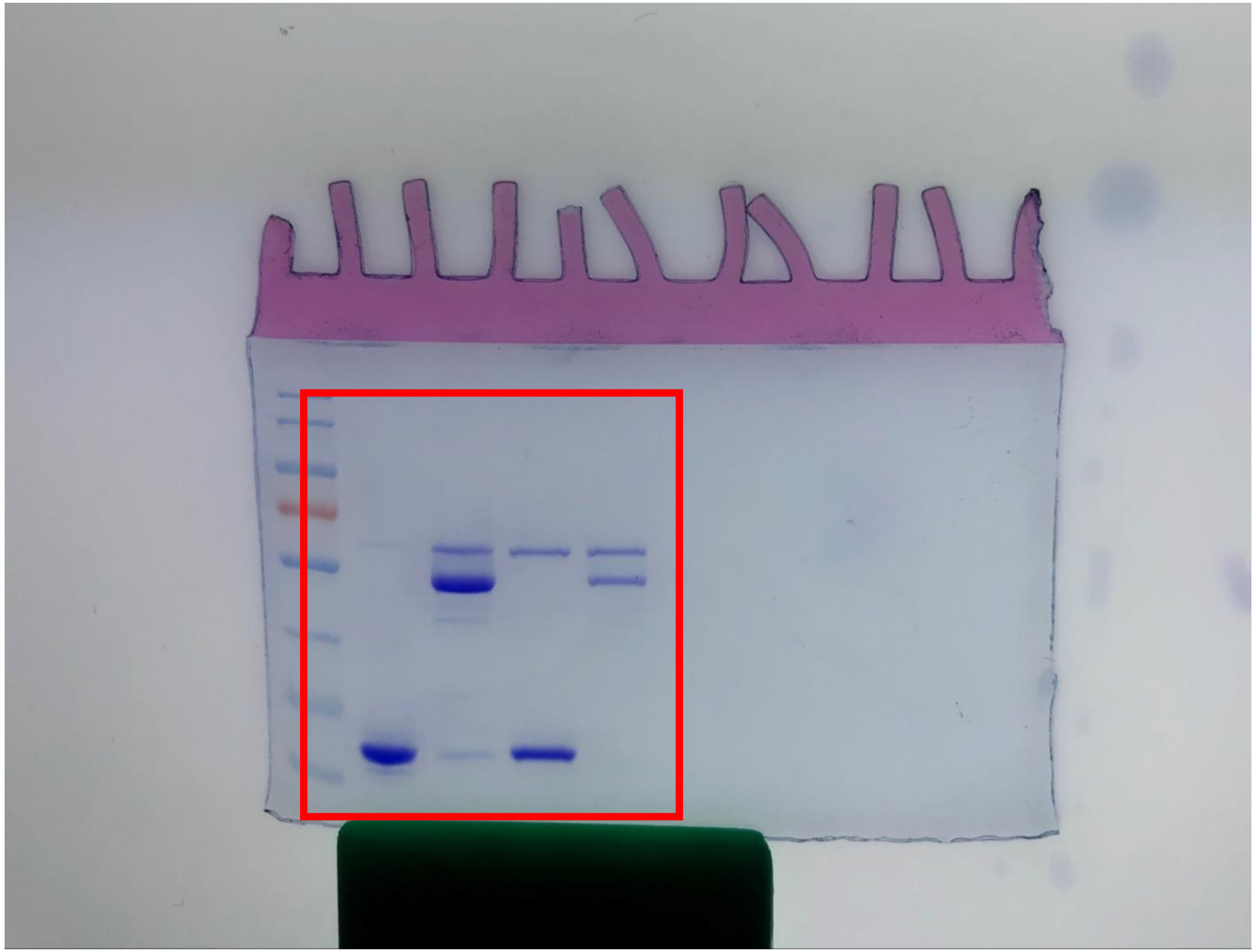

Figure 6-source data 5

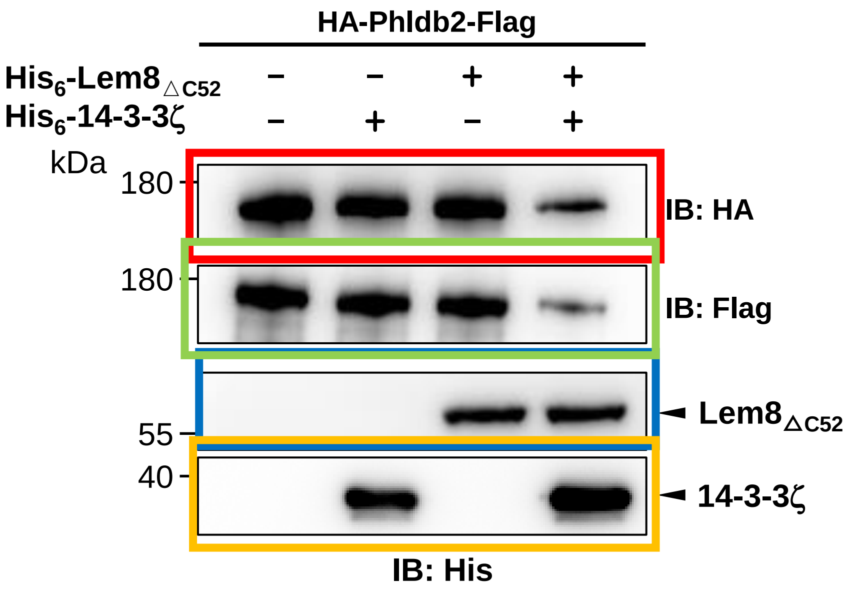

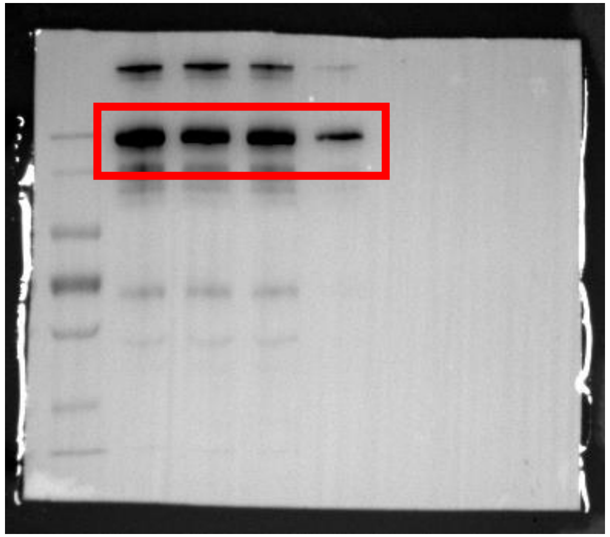

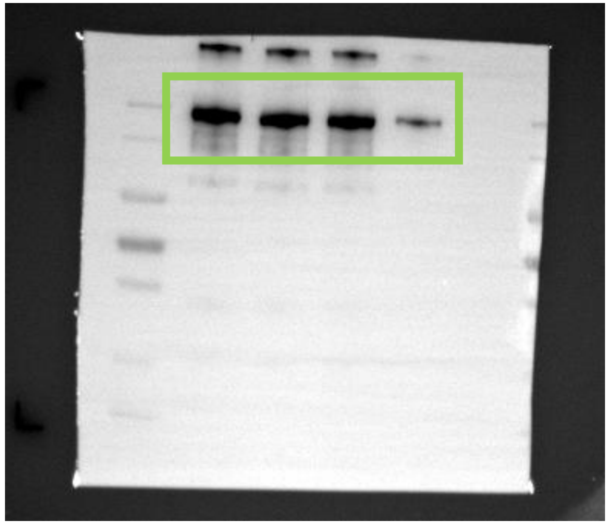

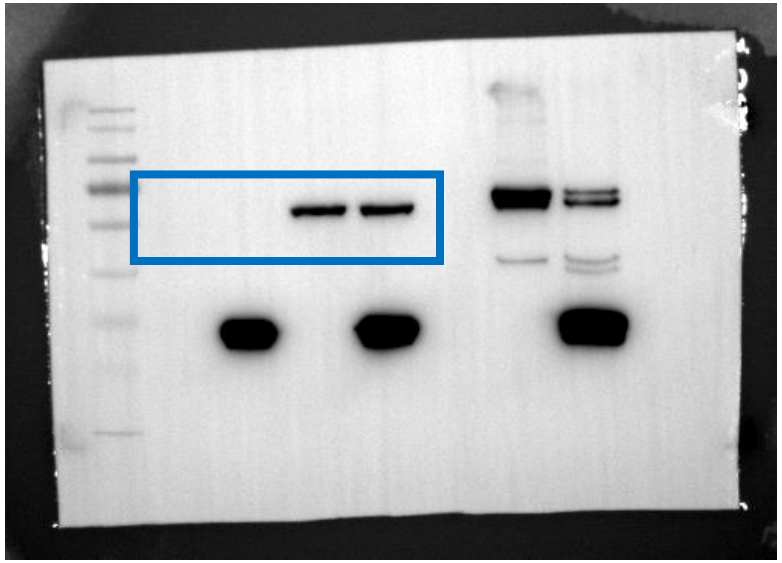

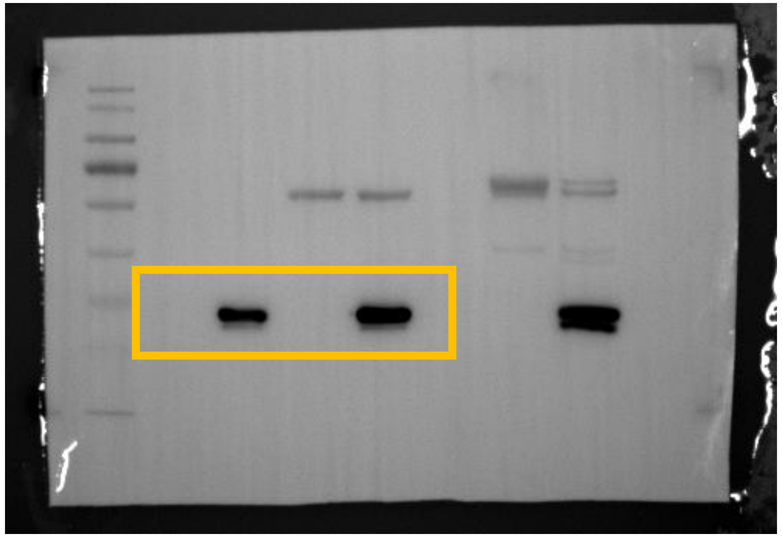

Figure 7-source data 1

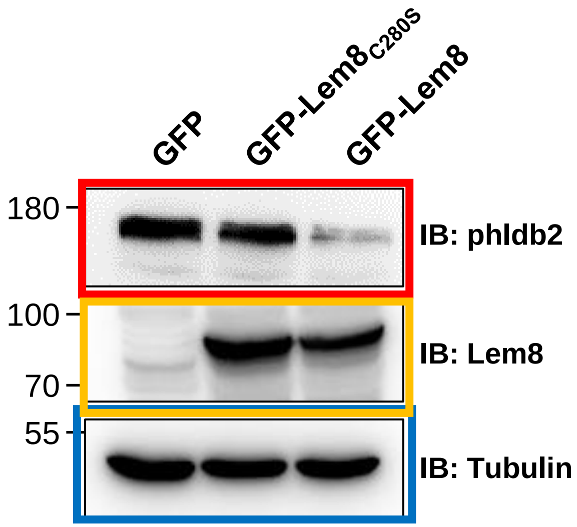

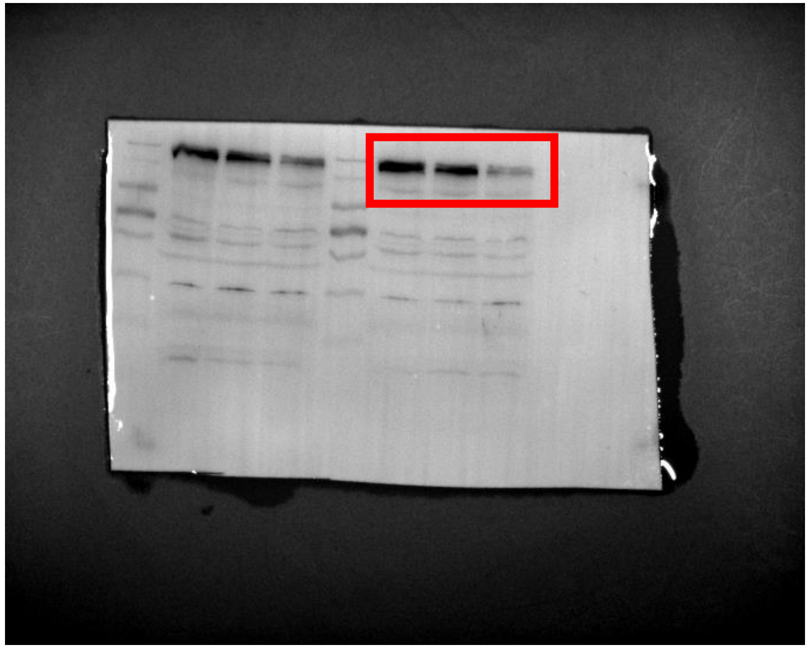

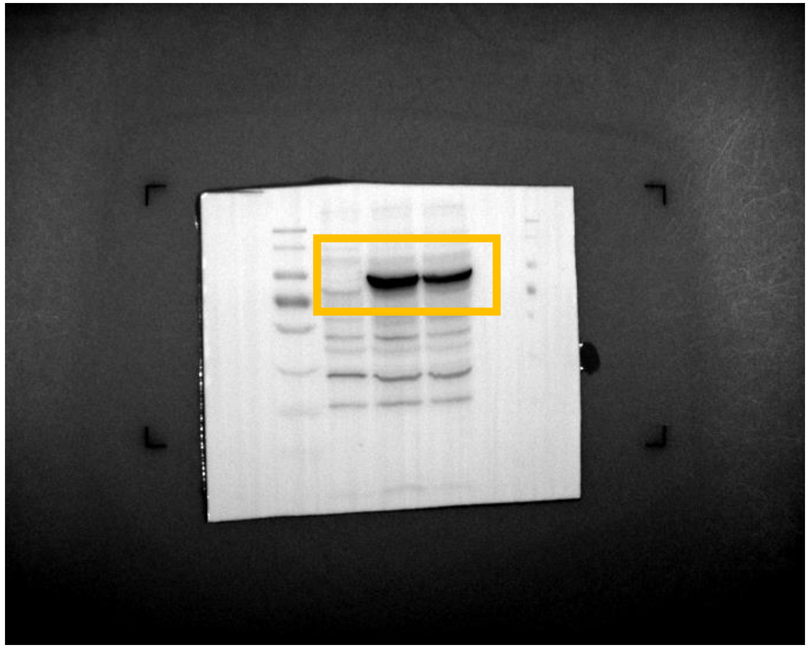

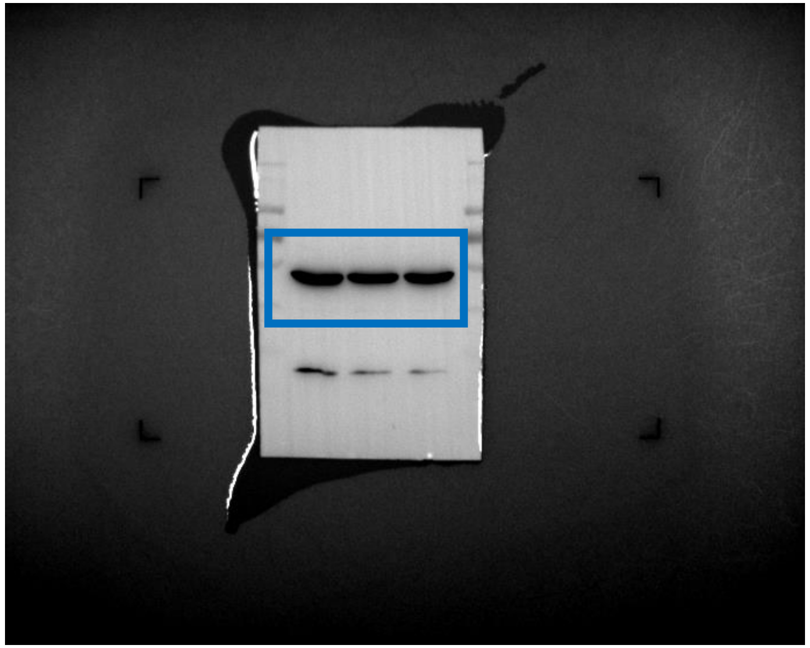

Figure 7-figure supplement 1-source data 1

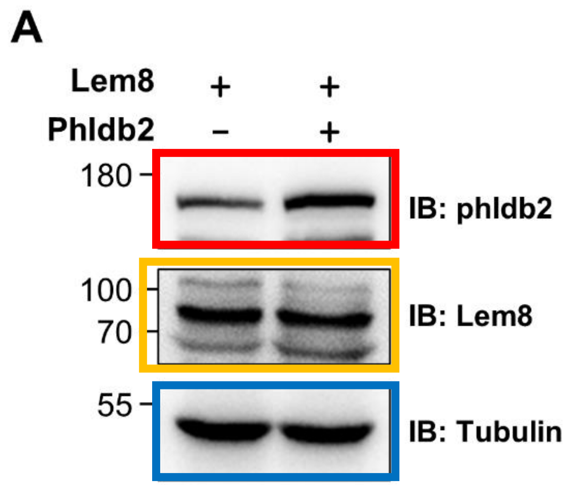

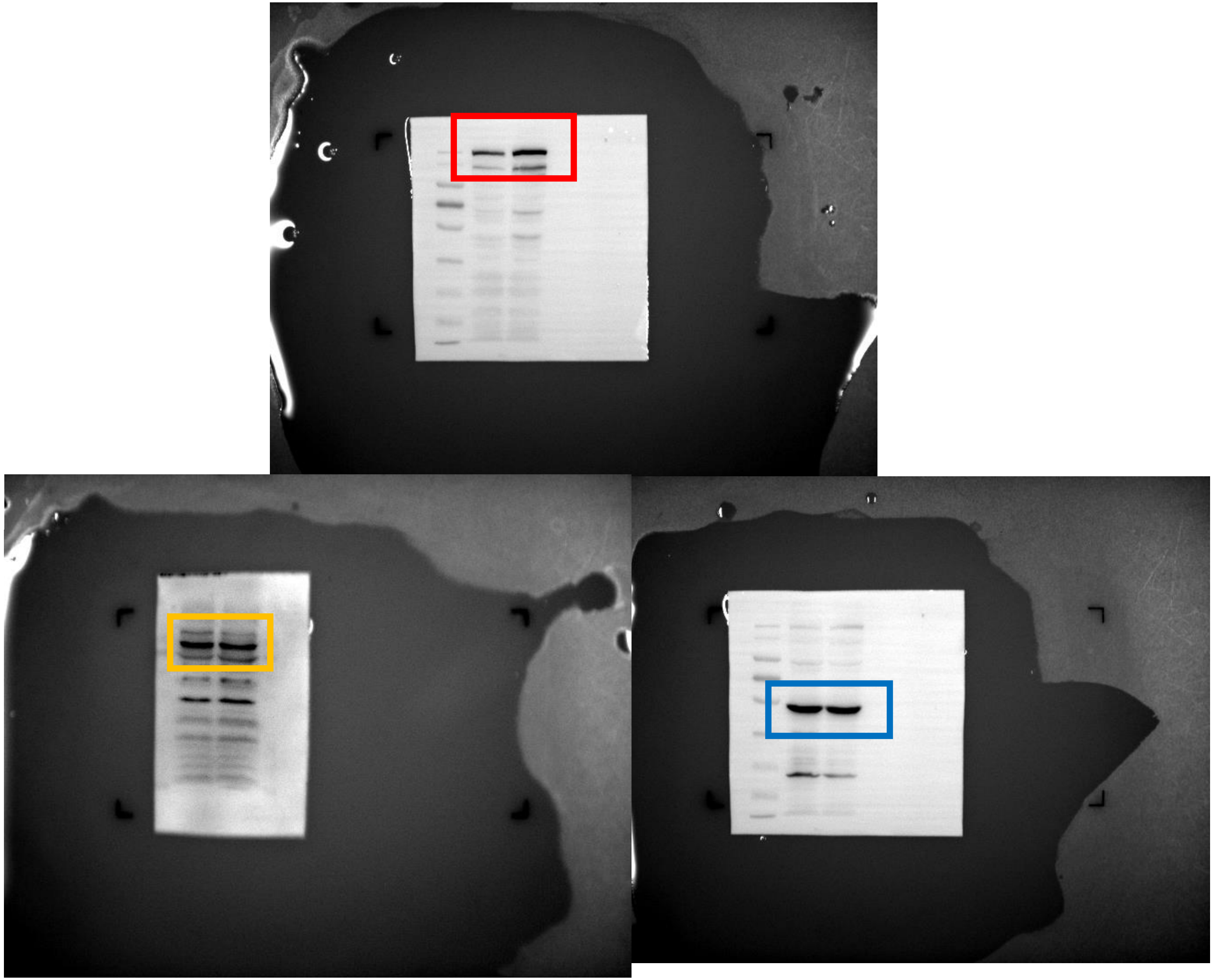

Supplement: Source data 1. [file elife-73220-data1.zip › Figures and Raw data(revision).pdf]
