# Supplementary material for: Legionella pneumophila regulates host cell motility by targeting Phldb2 with a 14-3-3ζ-dependent protease effector
Source: eLife. 2022 Feb 17;11:e73220. doi: 10.7554/eLife.73220 (PMC8871388; doi:10.7554/eLife.73220)
Supplement: Source data 1. [file elife-73220-data1.zip › source data (revision)/Figure 1-figure supplement 2-source data 1/Figure 1-figure supplement 2-source data 1 legend.docx]

**B.** Intracellular growth of the Δ*lem8* strain in *D. discoideum*. *D. discoideum* were infected with the indicated bacterial strains at an MOI of 0.1, and the intracellular growth was determined at a 24-h interval for 72 h (left panel). The expression and translocation of Lem8 in each strain was probed with Lem8 specific antibodies (right panel). ICDH and Tubulin were used as loading controls for bacterial and host cells, respectively. Similar results were obtained in three independent experiments.
