# Supplementary material for: Legionella pneumophila regulates host cell motility by targeting Phldb2 with a 14-3-3ζ-dependent protease effector
Source: eLife. 2022 Feb 17;11:e73220. doi: 10.7554/eLife.73220 (PMC8871388; doi:10.7554/eLife.73220)
Supplement: Source data 1. [file elife-73220-data1.zip › source data (revision)/Figure 1-figure supplement 2-source data 2/Figure 1-figure supplement 2-source data 2 legend.docx]

**C.** Intracellular growth of Δ*lem8* strain in BMDMs. The bacterial strains were used to infect BMDMs at an MOI of 0.1 and the intracellular growth was monitored at the indicated time points (left panel). Similar results were obtained in three independent experiments.
