# Supplementary material for: Legionella pneumophila regulates host cell motility by targeting Phldb2 with a 14-3-3ζ-dependent protease effector
Source: eLife. 2022 Feb 17;11:e73220. doi: 10.7554/eLife.73220 (PMC8871388; doi:10.7554/eLife.73220)
Supplement: Source data 1. [file elife-73220-data1.zip › source data (revision)/Figure 1-source data 1/Figure 1-source data 1 legend.docx]

**B.** Lem8 is translocated into mammalian cells via the Dot/Icm transporter. U937 cells were infected with wild-type *L. pneumophila* or a *dotA^-^* mutant expressing a β-lactamase-Lem8 fusion. One hour after infection, the CCF4-AM fluorescence substrate was added into the cultures and the cells were incubated for another 2 h at room temperature before image acquisition. Cells emitting blue fluorescence signals were quantitated by counting at least 500 cells in each experiment done in triplicate. Resutls shown are mean ± s.e. from one representative experiment.
