# Supplementary material for: Legionella pneumophila regulates host cell motility by targeting Phldb2 with a 14-3-3ζ-dependent protease effector
Source: eLife. 2022 Feb 17;11:e73220. doi: 10.7554/eLife.73220 (PMC8871388; doi:10.7554/eLife.73220)
Supplement: Source data 1. [file elife-73220-data1.zip › source data (revision)/Figure 1-source data 2/Figure 1-source data 2 legend.docx]

**C.** Expression profile of *lem8* in *L. pneumophila* grown in AYET broth. Bacteria grown to stationary phase were diluted at 1:20 in fresh medium and subcultures were grown in a shaker. Bacterial growth was monitored by measuring OD_600_ at the indicated time points. Equal amounts of bacterial cells were lysed for measurement of Lem8 levels by immunoblotting with Lem8-specific antibodies. The metabolic protein isocitrate dehydrogenase (ICDH) was probed as loading control.
