# Supplementary material for: Legionella pneumophila regulates host cell motility by targeting Phldb2 with a 14-3-3ζ-dependent protease effector
Source: eLife. 2022 Feb 17;11:e73220. doi: 10.7554/eLife.73220 (PMC8871388; doi:10.7554/eLife.73220)
Supplement: Source data 1. [file elife-73220-data1.zip › source data (revision)/Figure 1-source data 3/Figure 1-source data 3 legend.docx]

**D.** Lem8 is toxic to yeast in a manner that requires the predicted Cys-His-Asp motif. Yeast strains expressing Lem8 or the indicated mutants from the galactose-inducible promotor were serially diluted and spotted on the indicated media. The plates were incubated at 30°C for 48 h before image acquisition. The expression of Lem8 and its mutants induced by galactose were determined by immunoblotting with Lem8-specific antibodies. The 3-phosphoglycerate kinase (PGK) was detected as loading control.
