# Supplementary material for: Legionella pneumophila regulates host cell motility by targeting Phldb2 with a 14-3-3ζ-dependent protease effector
Source: eLife. 2022 Feb 17;11:e73220. doi: 10.7554/eLife.73220 (PMC8871388; doi:10.7554/eLife.73220)
Supplement: Source data 1. [file elife-73220-data1.zip › source data (revision)/Figure 2-figure supplement 1-source data 1/Figure 2-figure supplement 1-source data 1 legend.docx]

**Fig. S3** **Phospohorylation of Lem8 is not required for 14-3-3ζ binding.** Lysates of HEK293T cells expressing indicated HA tagged proteins were subjected to immunoprecipitation with agarose beads coated with the HA antibody. The precipitates, as well as His_6_-Lem8 purified from *E coli* were resolved by SDS-PAGE and probed by immunoblotting with a pan phospho-serine/threonine antibody, the HA specific antibody and the His_6_-specific antibody, respectively.
