# Supplementary material for: Legionella pneumophila regulates host cell motility by targeting Phldb2 with a 14-3-3ζ-dependent protease effector
Source: eLife. 2022 Feb 17;11:e73220. doi: 10.7554/eLife.73220 (PMC8871388; doi:10.7554/eLife.73220)
Supplement: Source data 1. [file elife-73220-data1.zip › source data (revision)/Figure 2-source data 1/Figure 2-source data 1 legend.docx]

**B.** Lem8 and 14-3-3ζ form a protein complex in mammalian cell. Total lysates of HEK293T cells transfected with indicated plasmid combinations were immunoprecipitated with a Flag-specific antibody (left panels) or GFP-specific antibodies (right panels), and the precipitates were probed with both Flag and GFP antibodies. Similar results were obtained from at least three independent experiments and the data shown here were from one representative experiment.
