# Supplementary material for: Legionella pneumophila regulates host cell motility by targeting Phldb2 with a 14-3-3ζ-dependent protease effector
Source: eLife. 2022 Feb 17;11:e73220. doi: 10.7554/eLife.73220 (PMC8871388; doi:10.7554/eLife.73220)
Supplement: Source data 1. [file elife-73220-data1.zip › source data (revision)/Figure 2-source data 2/Figure 2-source data 2 legend.docx]

**C.** Lem8 directly interacts with 14-3-3ζ. GST-14-3-3ζ was incubated with His_6_-Lem8 or His_6_-Lem8_C280S_, and the potential protein complex was captured by glutathione beads for 1 h at 4°C. After extensive washing, bound proteins were solublized with SDS loading buffer, and proteins were detected by Coomassie brilliant blue staining after being resolved by SDS/PAGE.
