# Supplementary material for: Legionella pneumophila regulates host cell motility by targeting Phldb2 with a 14-3-3ζ-dependent protease effector
Source: eLife. 2022 Feb 17;11:e73220. doi: 10.7554/eLife.73220 (PMC8871388; doi:10.7554/eLife.73220)
Supplement: Source data 1. [file elife-73220-data1.zip › source data (revision)/Figure 3-figure supplement 1-source data 1/Figure 3-figure supplement 1-source data 1 legend.docx]

**A.** Determination of the self-cleavage site of Lem8 by mass spectrometry. A diagram of the sequence containing the recognition site with the two diagnostic peptides used to determine the cleavage site (top panel). Protein bands corresponding to full-length and cleaved Lem8 band was excised ( lower left panel), digested with trypsin and analyzed by mass spectrometry. The semi-tryptic peptide -L_464_CEKAPQPTPQRQ_476_- is present in cleaved samples but not in samples of full-length Lem8, whereas the fragment -A_478_QSLSAETER_487_- was only detected in samples of the full-length protein (lower right panel), supporting the notion that the cleavage site lies between Gln476 and Arg477 described in Fig.3C.
