# Supplementary material for: Legionella pneumophila regulates host cell motility by targeting Phldb2 with a 14-3-3ζ-dependent protease effector
Source: eLife. 2022 Feb 17;11:e73220. doi: 10.7554/eLife.73220 (PMC8871388; doi:10.7554/eLife.73220)
Supplement: Source data 1. [file elife-73220-data1.zip › source data (revision)/Figure 3-figure supplement 1-source data 2/Figure 3-figure supplement 1-source data 2 legend.docx]

**B.** Self-cleavage of Lem8 removes GFP fused to its carboxyl end. GFP was fused to the indicated alleles of Lem8 and the fusion proteins were individually expressed in HEK293T cells by transfection. Samples resolved by SDS-PAGE were detected by immunoblotting with GFP-specific antibodies. Results shown were one representative from three independent experiments with similar results.
