# Supplementary material for: Legionella pneumophila regulates host cell motility by targeting Phldb2 with a 14-3-3ζ-dependent protease effector
Source: eLife. 2022 Feb 17;11:e73220. doi: 10.7554/eLife.73220 (PMC8871388; doi:10.7554/eLife.73220)
Supplement: Source data 1. [file elife-73220-data1.zip › source data (revision)/Figure 3-figure supplement 1-source data 3/Figure 3-figure supplement 1-source data 3 legend.docx]

**Fig. 3-figure supplement 1 Identification of the self-cleavage sites of Lem8**.

**C.** The self-cleavage site of the 4A mutant. Protein bands from stained SDS-PAGE gels were excised and analyzed similarly as described in A. The tryptic fragment A_468_PQPTPAAAAQSLSAETER_487_- was detected only in samples prepared from the cleaved protein but not full-length protein. Identical results were obtained in multiple samples analyzed by two different mass spectromentry facilities.
