# Supplementary material for: Legionella pneumophila regulates host cell motility by targeting Phldb2 with a 14-3-3ζ-dependent protease effector
Source: eLife. 2022 Feb 17;11:e73220. doi: 10.7554/eLife.73220 (PMC8871388; doi:10.7554/eLife.73220)
Supplement: Source data 1. [file elife-73220-data1.zip › source data (revision)/Figure 3-source data 1/Figure 3-source data 1 legend.docx]

**A.** Self-processing of Lem8 requires 14-3-3ζ. His_6_-Lem8 or His_6_-Lem8_C280S_ was incubated with His_6_-14-3-3ζ for 2 h, proteins resolved by SDS-PAGE were detected by Coomassie brilliant blue staining. The cysteine protease inhibitor E64 was added to the indicated samples.
