# Supplementary material for: Legionella pneumophila regulates host cell motility by targeting Phldb2 with a 14-3-3ζ-dependent protease effector
Source: eLife. 2022 Feb 17;11:e73220. doi: 10.7554/eLife.73220 (PMC8871388; doi:10.7554/eLife.73220)
Supplement: Source data 1. [file elife-73220-data1.zip › source data (revision)/Figure 3-source data 2/Figure 3-source data 2 legend.docx]

**B.** The 14-3-3 protein from *D. discoideum* induces the self-cleavage of Lem8. His_6_-Lem8 was incubated with GST-14-3-3ζ or GST-14-3-3Dd for the indicated time and the mixtures separated by SDS-PAGE were detected by immunoblotting with antibodies specific for Lem8 and GST, respectively.
