# Supplementary material for: Legionella pneumophila regulates host cell motility by targeting Phldb2 with a 14-3-3ζ-dependent protease effector
Source: eLife. 2022 Feb 17;11:e73220. doi: 10.7554/eLife.73220 (PMC8871388; doi:10.7554/eLife.73220)
Supplement: Source data 1. [file elife-73220-data1.zip › source data (revision)/Figure 3-source data 3/Figure 3-source data 3 legend.docx]

**C.** Determination of the self-cleavage site of Lem8. His_6_-Lem8 was incubated with His_6_-14-3-3ζ for 16 h, proteins were resolved by SDS-PAGE, stained with Coomassie brilliant blue. Protein bands corresponding to full-length and cleaved Lem8 band was excised, digested with trypsin and analyzed by mass spectrometry. The detection of the semi-tryptic peptide -L_464_CEKAPQPTPQRQ_476_- in cleaved samples suggested that the cleavage site lies between Gln476 and Arg477.
