# Supplementary material for: Legionella pneumophila regulates host cell motility by targeting Phldb2 with a 14-3-3ζ-dependent protease effector
Source: eLife. 2022 Feb 17;11:e73220. doi: 10.7554/eLife.73220 (PMC8871388; doi:10.7554/eLife.73220)
Supplement: Source data 1. [file elife-73220-data1.zip › source data (revision)/Figure 3-source data 4/Figure 3-source data 4 legend.docx]

**D.** Mutations in cleavage site does not abolish Lem8 self-processing. Recombinant protein of Lem8 and the 4A mutant were each incubated with His_6_-14-3-3ζ for 4 h. Proteins resolved by SDS-PAGE were detected by Coomassie brilliant blue.
