# Supplementary material for: Legionella pneumophila regulates host cell motility by targeting Phldb2 with a 14-3-3ζ-dependent protease effector
Source: eLife. 2022 Feb 17;11:e73220. doi: 10.7554/eLife.73220 (PMC8871388; doi:10.7554/eLife.73220)
Supplement: Source data 1. [file elife-73220-data1.zip › source data (revision)/Figure 4-figure supplement 1-source data 1/Figure 4-figure supplement 1-source data 1 legend.docx]

**Figure 4-figure supplement 1 Verification of Lem8-mediated cleavage of candidate proteins and its cleavage of phldb2 at multiple sites**

**A.** Cleavage of substrate candidates by Lem8. Flag- or HA- tagged Rasgrp2, Pak6, Exoc8, Ankrd13B, Chkb, Ppp6R1, Kiaa1033, Gnal and Gpr61 expressed in HEK293T cells each was immunoprecipitated with antibodies specific for Flag or HA. Proteins eluted with 3×Flag or HA peptides were incubated with His-Lem8 and His_6_-14-3-3ζ. Samples resolved with SDS-PAGE were probed by immunoblotting with a Flag- or HA- specific antibody. Note that Flag- or HA-GPR61 did not express so it was not examined. Results shown were one representative from three independent experiments with similar results.
