# Supplementary material for: Legionella pneumophila regulates host cell motility by targeting Phldb2 with a 14-3-3ζ-dependent protease effector
Source: eLife. 2022 Feb 17;11:e73220. doi: 10.7554/eLife.73220 (PMC8871388; doi:10.7554/eLife.73220)
Supplement: Source data 1. [file elife-73220-data1.zip › source data (revision)/Figure 4-figure supplement 1-source data 2/Figure 4-figure supplement 1-source data 2 legend.docx]

**Figure 4-figure supplement 1 Verification of Lem8-mediated cleavage of candidate proteins and its cleavage of phldb2 at multiple sites**

**B.** Lem8 causes redistribution of Phldb2 in cells. Hela cells were transfected to express the indicated mCherry fusion proteins. 24 h after transfection, cells were fixed and immnostained with anti-Phldb2 antibodies. The nuclei were stained by Hoechst 33342. Images were acquired with a Zeiss LSM 880 confocal microscope. Phldb2, green (GFP); Lem8 and its mutants, red (mCherry); nuclei, blue (Hoechst). The percentage of Phldb2 positive cells was calculated in Lem8 positive cells (Right panel). Bar, 10 μm.
