# Supplementary material for: Legionella pneumophila regulates host cell motility by targeting Phldb2 with a 14-3-3ζ-dependent protease effector
Source: eLife. 2022 Feb 17;11:e73220. doi: 10.7554/eLife.73220 (PMC8871388; doi:10.7554/eLife.73220)
Supplement: Source data 1. [file elife-73220-data1.zip › source data (revision)/Figure 4-figure supplement 1-source data 3/Figure 4-figure supplement 1-source data 3 legend.docx]

**Figure 4-figure supplement 1 Verification of Lem8-mediated cleavage of candidate proteins and its cleavage of phldb2 at multiple sites**

**C.** Mutations were introduced into HA-Phldb2-Flag to replace residues Arg_1111_ and Gln_1112_ (Phldb2_AA1_) or Gln_1112_ and Arg_1113_ (Phldb2_AA2_) with alanine, respectively. The two mutants were co-expressed in HEK293T cells with Lem8 or Lem8_C280S_. Samples were resolved with SDS-PAGE, and probed by immunoblotting with the antibody specific to HA and Flag_,_ respectively.
