# Supplementary material for: Legionella pneumophila regulates host cell motility by targeting Phldb2 with a 14-3-3ζ-dependent protease effector
Source: eLife. 2022 Feb 17;11:e73220. doi: 10.7554/eLife.73220 (PMC8871388; doi:10.7554/eLife.73220)
Supplement: Source data 1. [file elife-73220-data1.zip › source data (revision)/Figure 4-figure supplement 1-source data 4/Figure 4-figure supplement 1-source data 4 legend.docx]

**Fig. 4-figure supplement 1** **Verification of Lem8-mediated cleavage of candidate proteins and its cleavage of phldb2 at multiple sites**

**D.** Lem8 removes the GFP tag fused to the amino end of Phldb2 deletion mutants. GFP was fused to the amino end of Phldb2 and the indicated truncation mutants. The fusion proteins were individually co-expressed with HA-Lem8 or HA-Lem8_C280S_ in HEK293T cells by transfection. Samples resolved by SDS-PAGE were detected by immunoblotting with GFP-specific antibodies. Results shown were one representative from three independent experiments with similar results.
