# Supplementary material for: Legionella pneumophila regulates host cell motility by targeting Phldb2 with a 14-3-3ζ-dependent protease effector
Source: eLife. 2022 Feb 17;11:e73220. doi: 10.7554/eLife.73220 (PMC8871388; doi:10.7554/eLife.73220)
Supplement: Source data 1. [file elife-73220-data1.zip › source data (revision)/Figure 4-figure supplement 1-source data 5/Figure 4-figure supplement 1-source data 5 legend.docx]

**Fig. 4-figure supplement 1** **Verification of Lem8-mediated cleavage of candidate proteins and its cleavage of phldb2 at multiple sites**

**E.** Cleavage of Phldb2 is undetectable during *L. pneumophila* infection. HEK293T cells transfected to express FcγRII receptor were infected with the indicated bacterial strains. 2 h after infection, the protein levels of Phldb2, as well as the translocation and expression of Lem8, were probed with the appropriate antibodies with Tubulin and ICDH as loading control, respectively. Results shown were one representative from three independent experiments with similar results. Bacterial strains: I, No infection; II, Lp02 (WT); III, *dotA*^-^(defective in Dot/Icm); IV, Lp02Δlem8; V, Lp02Δlem8(pLem8); VI, Lp02Δlem8(pLem8_C280S_).
