# Supplementary material for: Legionella pneumophila regulates host cell motility by targeting Phldb2 with a 14-3-3ζ-dependent protease effector
Source: eLife. 2022 Feb 17;11:e73220. doi: 10.7554/eLife.73220 (PMC8871388; doi:10.7554/eLife.73220)
Supplement: Source data 1. [file elife-73220-data1.zip › source data (revision)/Figure 4-source data 1/Figure 4-source data 1 legend.docx]

**B.** Lem8 reduces the protein levels of endogenous Phldb2 in mammalian cells. Lem8 and the indicated mutants were individually expressed in HEK293T cells by transfection. 24 h after transfection, the samples were resolved by SDS-PAGE and detected by immunoblotting with anti-Phldb2 antibodies. Tubulin was used as a loading control. Results shown were one representative from three independent experiments with similar results.
