# Supplementary material for: Legionella pneumophila regulates host cell motility by targeting Phldb2 with a 14-3-3ζ-dependent protease effector
Source: eLife. 2022 Feb 17;11:e73220. doi: 10.7554/eLife.73220 (PMC8871388; doi:10.7554/eLife.73220)
Supplement: Source data 1. [file elife-73220-data1.zip › source data (revision)/Figure 4-source data 2/Figure 4-source data 2 legend.docx]

**C.** Lem8 cleaves exogenous Phldb2 in mammalian cells. HA and Flag tag were fused to the amino and carboxyl end of Phldb2 respectively and the double tagged protein was co-expressed in HEK293T cells with Lem8 or each of the mutants. 24 h after transfection, the samples were resolved by SDS-PAGE and probed by a HA-specific antibody and a Flag-specific antibody, respectively. Tubulin was detected as a loading control. Results shown were one representative from three independent experiments with similar results.
