# Supplementary material for: Legionella pneumophila regulates host cell motility by targeting Phldb2 with a 14-3-3ζ-dependent protease effector
Source: eLife. 2022 Feb 17;11:e73220. doi: 10.7554/eLife.73220 (PMC8871388; doi:10.7554/eLife.73220)
Supplement: Source data 1. [file elife-73220-data1.zip › source data (revision)/Figure 4-source data 3/Figure 4-source data 3 legend.docx]

**D.** Lem8 alters the subcellular distribution of GFP fused to Phldb2. GFP was fused to the amino end of Phldb2 and the protein was co-expressed in HEK293T cells with mCherry-Lem8 or each of the mutants. 24 h after transfection, cells were fixed and nucleus were stained by Hoechst 33342. The fluorescence Images of GFP (green), mCherry (red) and Hoechst (blue) were acquired with a Zeiss LSM 880 confocal microscope. The percentage of cells with membrane Phldb2 was calculated in Phldb2 and Lem8 positive cells (Right panel). Bar, 10 μm.
