# Supplementary material for: Legionella pneumophila regulates host cell motility by targeting Phldb2 with a 14-3-3ζ-dependent protease effector
Source: eLife. 2022 Feb 17;11:e73220. doi: 10.7554/eLife.73220 (PMC8871388; doi:10.7554/eLife.73220)
Supplement: Source data 1. [file elife-73220-data1.zip › source data (revision)/Figure 4-source data 4/Figure 4-source data 4 legend.docx]

**E.** 14-3-3ζ is required for the cleavage of Phldb2 by Lem8. HA-Phldb2-Flag was expressed in HEK293T cells, immunoprecipitated with a Flag-specific antibody, and eluted with 3×Flag peptides. Purified Phldb2 was incubated with His_6_-Lem8 or each of the mutants in reactions with or without His_6_-14-3-3ζ. Total proteins of all samples were resolved with SDS-PAGE, and probed by immunoblotting with a HA-specific antibody, a Flag-specific antibody and a His-specific antibody.
