# Supplementary material for: Legionella pneumophila regulates host cell motility by targeting Phldb2 with a 14-3-3ζ-dependent protease effector
Source: eLife. 2022 Feb 17;11:e73220. doi: 10.7554/eLife.73220 (PMC8871388; doi:10.7554/eLife.73220)
Supplement: Source data 1. [file elife-73220-data1.zip › source data (revision)/Figure 5-source data 1/Figure 5-source data 1 legend.docx]

**B.** The predicted coiled coil motif is critical for Lem8-mediated yeast toxicity. Yeast cells inducibly expressing Lem8 or mutant Lem8_GG_ were serially diluted and spotted onto the indicated media for 48 h (top panel). The expression of Lem8 and Lem8_GG_ was examined and PGK1 was probed as a loading control (lower panel).
