# Supplementary material for: Legionella pneumophila regulates host cell motility by targeting Phldb2 with a 14-3-3ζ-dependent protease effector
Source: eLife. 2022 Feb 17;11:e73220. doi: 10.7554/eLife.73220 (PMC8871388; doi:10.7554/eLife.73220)
Supplement: Source data 1. [file elife-73220-data1.zip › source data (revision)/Figure 5-source data 2/Figure 5-source data 2 legend.docx]

**C.** Lem8_GG_ loses the capacity to cleave Phldb2 in mammalian cells. Lysates of HEK293T cells expressing Lem8 or Lem8_GG_ were resolved by SDS-PAGE and detected by immunoblotting with antibodies specific for Phldb2 and Lem8, respectively. Tubulin was used as a loading control. Results shown were one representative from three independent experiments with similar results.
