# Supplementary material for: Legionella pneumophila regulates host cell motility by targeting Phldb2 with a 14-3-3ζ-dependent protease effector
Source: eLife. 2022 Feb 17;11:e73220. doi: 10.7554/eLife.73220 (PMC8871388; doi:10.7554/eLife.73220)
Supplement: Source data 1. [file elife-73220-data1.zip › source data (revision)/Figure 5-source data 3/Figure 5-source data 3 legend.docx]

**D.** The predicted coiled coil motif is required for self-processing of Lem8. The indicated alleles of Lem8-GFP were individually expressed in HEK293T cells by transfection. Samples resolved by SDS-PAGE were detected by immunoblotting with GFP-specific antibodies. Results shown were one representative from three independent experiments with similar results.
