# Supplementary material for: Legionella pneumophila regulates host cell motility by targeting Phldb2 with a 14-3-3ζ-dependent protease effector
Source: eLife. 2022 Feb 17;11:e73220. doi: 10.7554/eLife.73220 (PMC8871388; doi:10.7554/eLife.73220)
Supplement: Source data 1. [file elife-73220-data1.zip › source data (revision)/Figure 5-source data 4/Figure 5-source data 4 legend.docx]

**E.** Interactions between 14-3-3ζ and the Lem8_GG_ mutant. Lysates of 293T cells expressing Flag-14-3-3ζ with HA-Lem8 or HA-Lem8_GG_ were subjected to immunoprecipitation with the anti-HA antibody and the presence of 14-3-3ζ in the precipitates was probed with the Flag-specific antibody.
