# Supplementary material for: Legionella pneumophila regulates host cell motility by targeting Phldb2 with a 14-3-3ζ-dependent protease effector
Source: eLife. 2022 Feb 17;11:e73220. doi: 10.7554/eLife.73220 (PMC8871388; doi:10.7554/eLife.73220)
Supplement: Source data 1. [file elife-73220-data1.zip › source data (revision)/Figure 6-source data 1/Figure 6-source data 1 legend.docx]

**A.** The auto-processed form of Lem8 cleaves Phldb2 in cells. HA-Phldb2-Flag was co-expressed in HEK293T cells with Lem8 or the indicated truncation mutants including the self-processed form, Lem8_△C52_. 24 h after transfection, the samples were resolved by SDS-PAGE and probed by a HA-specific antibody and a Flag-specific antibody. Tubulin was used as a loading control. Results shown were one representative from three independent experiments with similar results.
