# Supplementary material for: Legionella pneumophila regulates host cell motility by targeting Phldb2 with a 14-3-3ζ-dependent protease effector
Source: eLife. 2022 Feb 17;11:e73220. doi: 10.7554/eLife.73220 (PMC8871388; doi:10.7554/eLife.73220)
Supplement: Source data 1. [file elife-73220-data1.zip › source data (revision)/Figure 6-source data 2/Figure 6-source data 2 legend.docx]

**B.** Lem8_∆C52_ causes redistribution of GFP-Phldb2. Truncations of Lem8, including Lem8_∆N25_, Lem8_∆C52_ and Lem8_∆C100_ fused to mCherry was individually expressed in HEK293T cells with GFP-Phldb2. 24 h after transfection, the fluorescence images were acquired with a Zeiss LSM 880 confocal microscope. The percentage of cells with membrane Phldb2 was calculated in Phldb2 and Lem8 positive cells (Right panel). Bar, 10 μm.
