# Supplementary material for: Legionella pneumophila regulates host cell motility by targeting Phldb2 with a 14-3-3ζ-dependent protease effector
Source: eLife. 2022 Feb 17;11:e73220. doi: 10.7554/eLife.73220 (PMC8871388; doi:10.7554/eLife.73220)
Supplement: Source data 1. [file elife-73220-data1.zip › source data (revision)/Figure 6-source data 3/Figure 6-source data 3 legend.docx]

**C.** The interaction between Lem8_△C52_ and 14-3-3ζ. Total lysates of HEK293T cells transfected with indicated plasmid combinations were immunoprecipitated with antibodies specific for HA (left panel) or Flag (right), and the precipitates were probed with both HA and Flag antibodies. Similar results were obtained from at least three independent experiments and the data shown here were from one representative experiment.
