# Supplementary material for: Legionella pneumophila regulates host cell motility by targeting Phldb2 with a 14-3-3ζ-dependent protease effector
Source: eLife. 2022 Feb 17;11:e73220. doi: 10.7554/eLife.73220 (PMC8871388; doi:10.7554/eLife.73220)
Supplement: Source data 1. [file elife-73220-data1.zip › source data (revision)/Figure 6-source data 4/Figure 6-source data 4 legend.docx]

**D.** Lem8_△C52_ directly interacts with 14-3-3ζ. Mixtures containing GST-14-3-3ζ and His_6_-Lem8_△C52_ were incubated with glutathione beads for 1 h at 4°C. After washing, samples resolved by SDS/PAGE were detected by Coomassie brilliant blue staining.
