# Supplementary material for: Legionella pneumophila regulates host cell motility by targeting Phldb2 with a 14-3-3ζ-dependent protease effector
Source: eLife. 2022 Feb 17;11:e73220. doi: 10.7554/eLife.73220 (PMC8871388; doi:10.7554/eLife.73220)
Supplement: Source data 1. [file elife-73220-data1.zip › source data (revision)/Figure 6-source data 5/Figure 6-source data 5 legend.docx]

**E.** The cleavage of Phldb2 by Lem8_△C52_ requires 14-3-3ζ. Purified HA-Phldb2-Flag from HEK293T was incubated with His_6_-Lem8_△C52_ in reactions with or without His_6_-14-3-3ζ. Total proteins of all samples were resolved with SDS-PAGE, and probed by immunoblotting with antibody specific for HA, Flag and His_6_, respectively.
