# Supplementary material for: Legionella pneumophila regulates host cell motility by targeting Phldb2 with a 14-3-3ζ-dependent protease effector
Source: eLife. 2022 Feb 17;11:e73220. doi: 10.7554/eLife.73220 (PMC8871388; doi:10.7554/eLife.73220)
Supplement: Source data 1. [file elife-73220-data1.zip › source data (revision)/Figure 7-figure supplement 1-source data 1/Figure 7-figure supplement 1-source data 1 legend.docx]

**Fig. 7-figure supplement 1 Overexpression of Phldb2 suppressed the inhibitory effects of Lem8 on cell migration**

A. Overexpression of Phldb2 in 293T cells stably expressing Lem8. A HEK293T-derived cell line stably expressing Lem8 was transfected with empty vector or HA-Phldb2 for 18 h, and the cell ysates were probed by immunoblotting with antibodies specific for Phldb2 or Lem8. Tubulin was used as a loading control. Results shown were one representative from three independent experiments with similar results.
