# Supplementary material for: Legionella pneumophila regulates host cell motility by targeting Phldb2 with a 14-3-3ζ-dependent protease effector
Source: eLife. 2022 Feb 17;11:e73220. doi: 10.7554/eLife.73220 (PMC8871388; doi:10.7554/eLife.73220)
Supplement: Source data 1. [file elife-73220-data1.zip › source data (revision)/Figure 7-figure supplement 1-source data 2/Figure 7-figure supplement 1-source data 2 legend.docx]

**Fig. 7-figure supplement 1 Overexpression of Phldb2 suppressed the inhibitory effects of Lem8 on cell migration**

**B.** Wound-healing scratch assay of cells expressing Phldb2. 18 h after transfection, monolayers were scratched using a pipette tip. Images of the wounds were captured at 2 h, 24 h and 48 h, respectively. Results shown were from a representative of three independent experiments from which the quantitation of wound healing was obtained by Image J (right panel).
