# Supplementary material for: Legionella pneumophila regulates host cell motility by targeting Phldb2 with a 14-3-3ζ-dependent protease effector
Source: eLife. 2022 Feb 17;11:e73220. doi: 10.7554/eLife.73220 (PMC8871388; doi:10.7554/eLife.73220)
Supplement: Source data 1. [file elife-73220-data1.zip › source data (revision)/Figure 7-source data 1/Figure 7-source data 1 legend.docx]

**A.** Establishment of cell lines stably expressing Lem8 or its enzymatically inactive mutant. HEK293T cells were transduced with lentiviral particles harboring the indicated plasmid at an MOI of 10 for two days, and the GFP-positive cells were isolated by a BD Influx™ cell sorter. Lysates of each cell lines were probed by immunoblotting with antibodies specific for Phldb2 or GFP. Tubulin was used as a loading control.
