# Supplementary material for: Legionella pneumophila regulates host cell motility by targeting Phldb2 with a 14-3-3ζ-dependent protease effector
Source: eLife. 2022 Feb 17;11:e73220. doi: 10.7554/eLife.73220 (PMC8871388; doi:10.7554/eLife.73220)
Supplement: Source data 1. [file elife-73220-data1.zip › source data (revision)/Figure 7-source data 2/Figure 7-source data 2 legend.docx]

**B.** Wound-healing scratch assay of the three stable cell lines. The three cell lines were individually seeded into 6 well plates. When reached confluency, cell monolayer of each cell lines was scratched using a pipette tip. Images of the wounds were captured at 2 h, 24 h and 48 h after making the scratches using an Olympus IX-83 fluorescence microscope. Images of a representative experiment were shown (left panel). The wound healing rates from three independent experiments was quantitated by Image J (right panel).
