# Supplementary material for: Legionella pneumophila regulates host cell motility by targeting Phldb2 with a 14-3-3ζ-dependent protease effector
Source: eLife. 2022 Feb 17;11:e73220. doi: 10.7554/eLife.73220 (PMC8871388; doi:10.7554/eLife.73220)
Supplement: Source data 1. [file elife-73220-data1.zip › source data (revision)/Figure 7-source data 3/Figure 7-source data 3 legend.docx]

**C-D.** Evaluation of the impact of Lem8 on cell migration in cells infected with *L. pneumophila*. HEK293T cells expressing the FcγII receptor (C) or Raw264.7 cells (D) were infected with opsonized bacteria of the indicated *L. pneumophila* strains at an MOI of 50 for 2 h. After washes, the wound-healing scratch assay was performed to evaluate the impact of infection on cell migration. Images of a representative experiment were shown (C-D, left panel) and the wound healing rate was analyzed by Image J (C-D, right panel).
