# Supplementary figures and images for: Legionella pneumophila regulates host cell motility by targeting Phldb2 with a 14-3-3ζ-dependent protease effector
Source: eLife. 2022 Feb 17;11:e73220. doi: 10.7554/eLife.73220 (PMC8871388; doi:10.7554/eLife.73220)

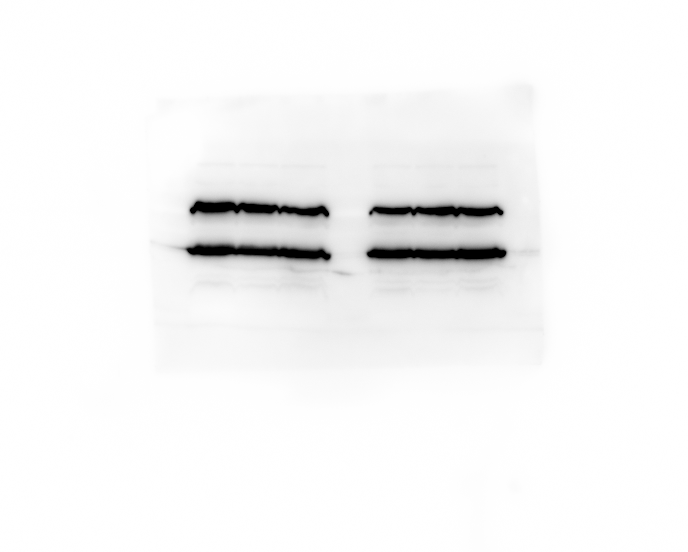

Supplement: Source data 1. [file elife-73220-data1.zip › source data (revision)/Figure 1-figure supplement 2-source data 1/Figure 1-figure supplement 2-2B ICDH.Tif]

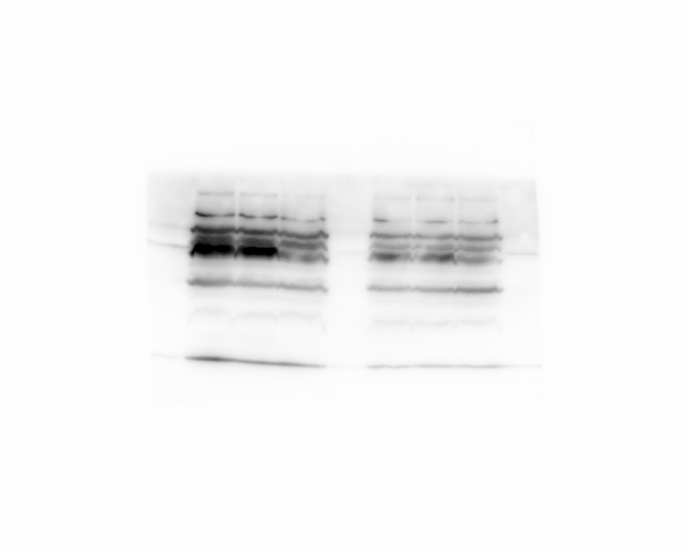

Supplement: Source data 1. [file elife-73220-data1.zip › source data (revision)/Figure 1-figure supplement 2-source data 1/Figure 1-figure supplement 2-2B Lem8 expression.Tif]

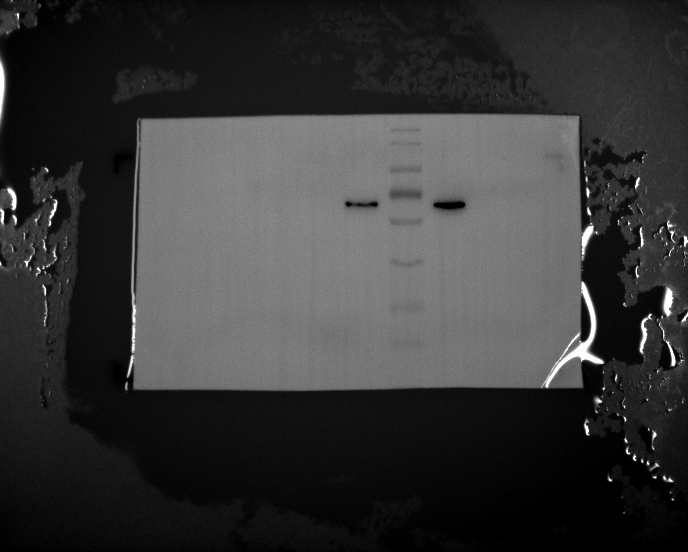

Supplement: Source data 1. [file elife-73220-data1.zip › source data (revision)/Figure 1-figure supplement 2-source data 1/Figure 1-figure supplement 2-2B Lem8 translocation.Tif]

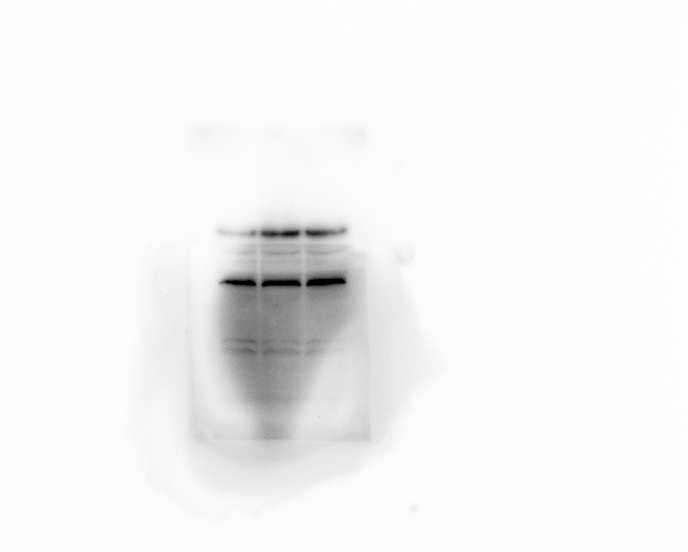

Supplement: Source data 1. [file elife-73220-data1.zip › source data (revision)/Figure 1-figure supplement 2-source data 1/Figure 1-figure supplement 2-2B Tubulin.Tif]

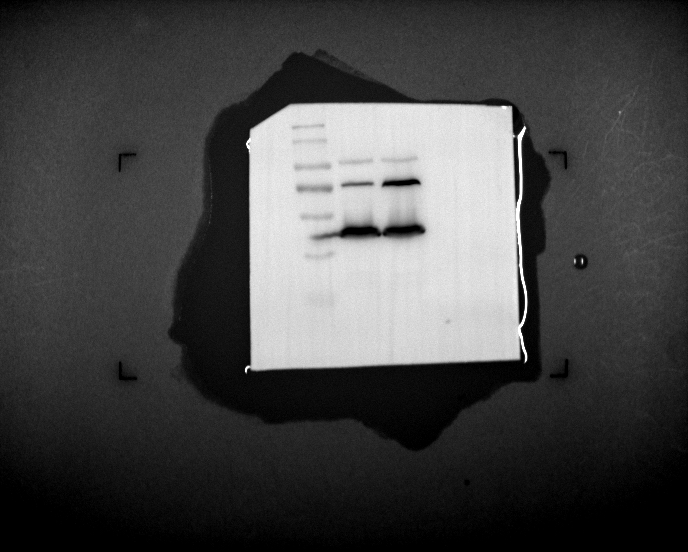

Supplement: Source data 1. [file elife-73220-data1.zip › source data (revision)/Figure 1-source data 1/Fig. 1B ICDH.jpg]

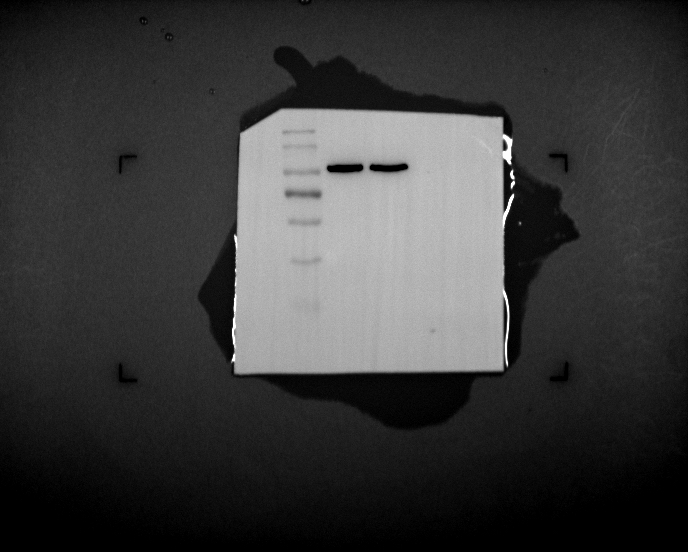

Supplement: Source data 1. [file elife-73220-data1.zip › source data (revision)/Figure 1-source data 1/Fig. 1B Lem8.jpg]

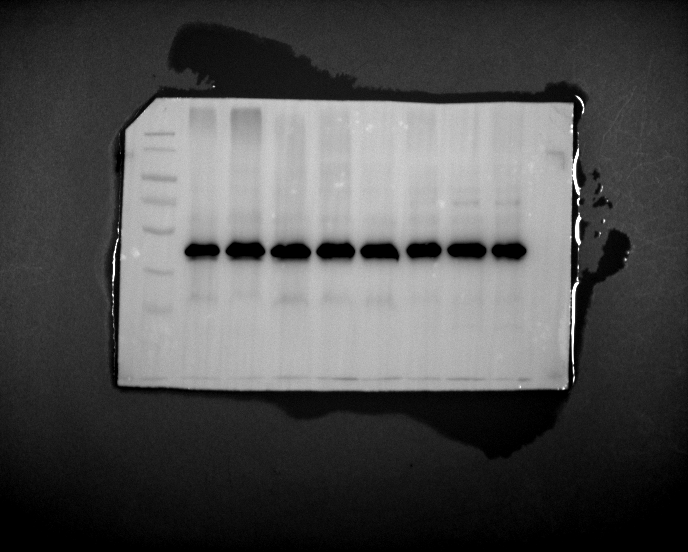

Supplement: Source data 1. [file elife-73220-data1.zip › source data (revision)/Figure 1-source data 2/Fig. 1C ICDH.Tif]

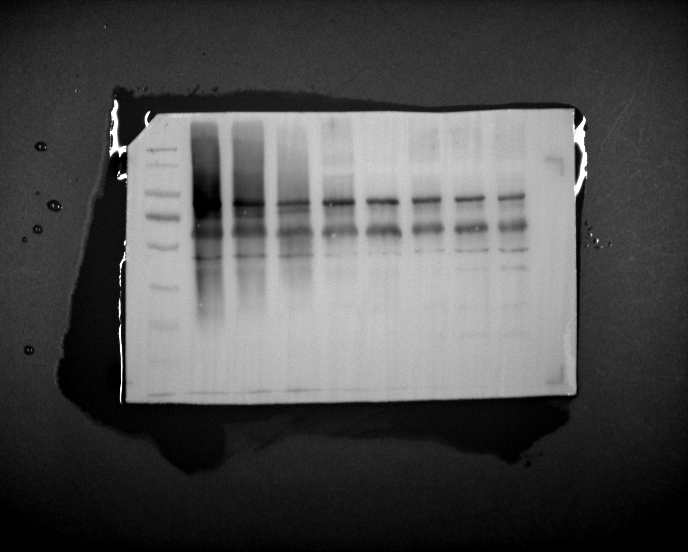

Supplement: Source data 1. [file elife-73220-data1.zip › source data (revision)/Figure 1-source data 2/Fig. 1C Lem8.Tif]

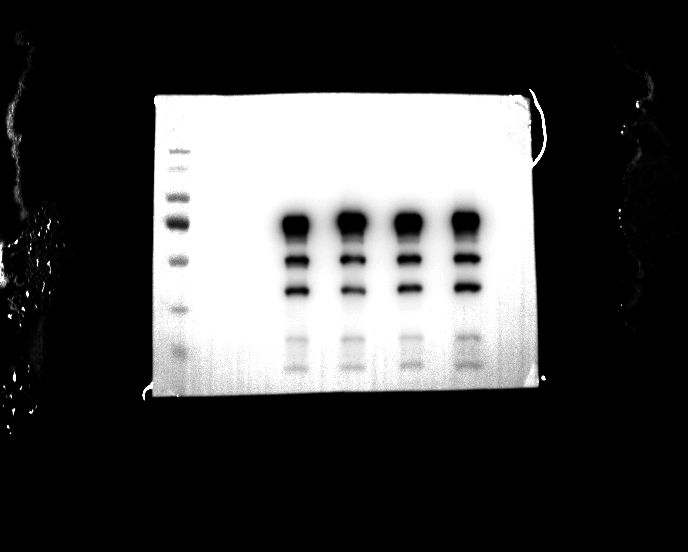

Supplement: Source data 1. [file elife-73220-data1.zip › source data (revision)/Figure 1-source data 3/Fig. 1D Lem8.Tif]

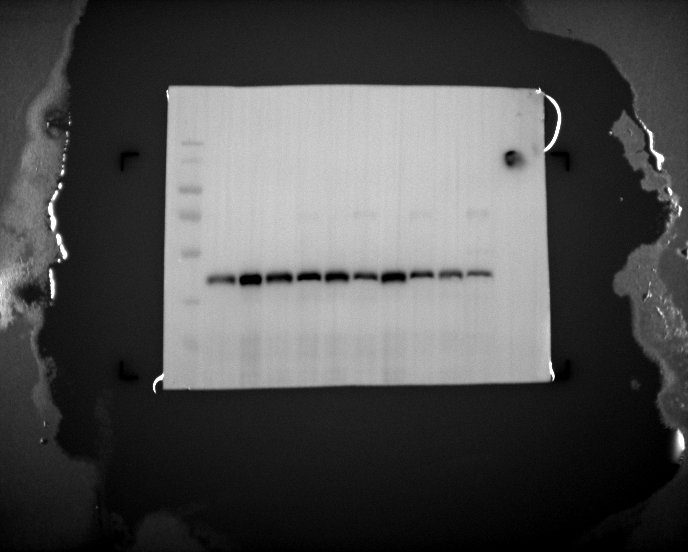

Supplement: Source data 1. [file elife-73220-data1.zip › source data (revision)/Figure 1-source data 3/Fig. 1D PGKalpha.Tif]

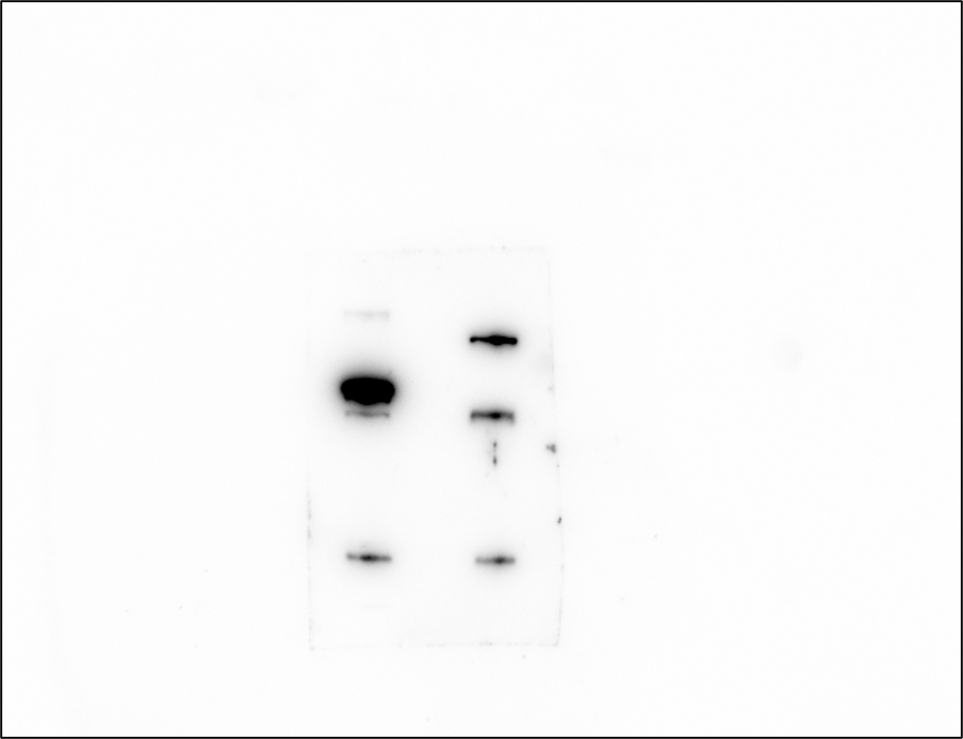

Supplement: Source data 1. [file elife-73220-data1.zip › source data (revision)/Figure 2-figure supplement 1-source data 1/Figure 2-figure supplement 1 HA.tif]

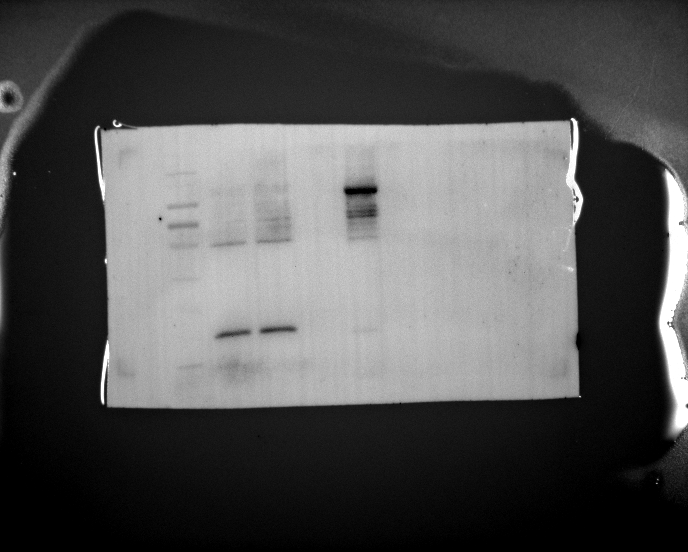

Supplement: Source data 1. [file elife-73220-data1.zip › source data (revision)/Figure 2-figure supplement 1-source data 1/Figure 2-figure supplement 1 pan-phosphorylation.Tif]

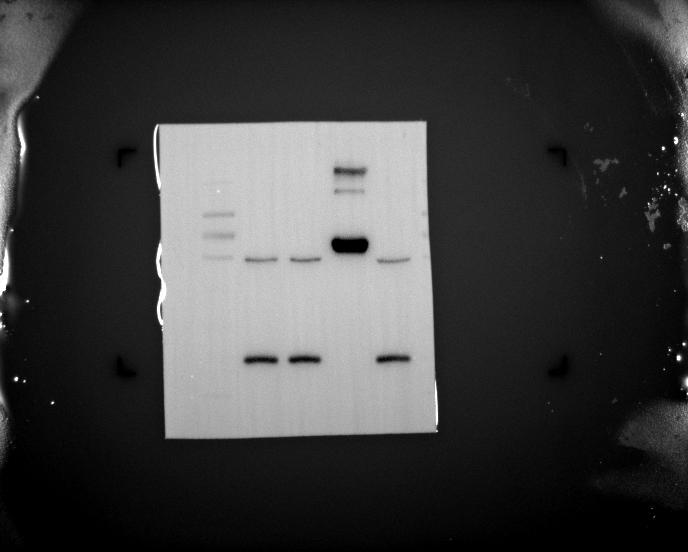

Supplement: Source data 1. [file elife-73220-data1.zip › source data (revision)/Figure 2-figure supplement 1-source data 1/Figure 2-figure supplement 1 His.Tif]

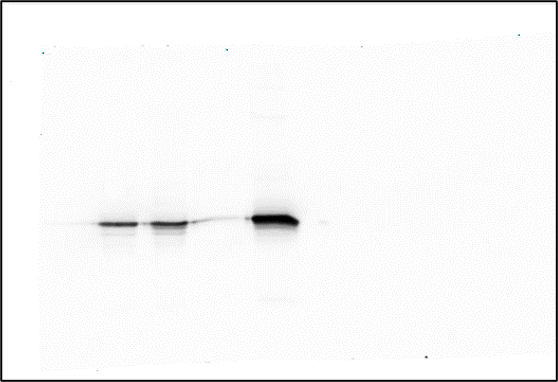

Supplement: Source data 1. [file elife-73220-data1.zip › source data (revision)/Figure 2-source data 1/Fig. 2B GFP-IP GFP-IB.tif]

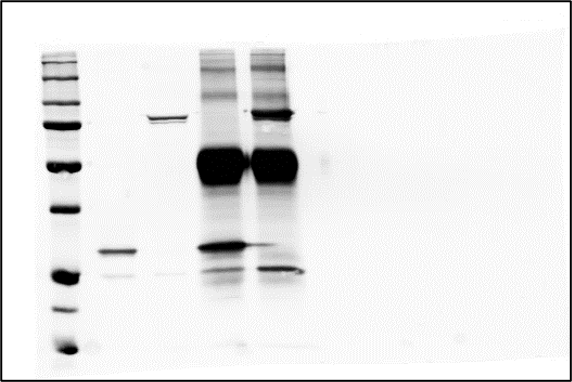

Supplement: Source data 1. [file elife-73220-data1.zip › source data (revision)/Figure 2-source data 1/Fig. 2B GFP-IP flag-IB.tif]

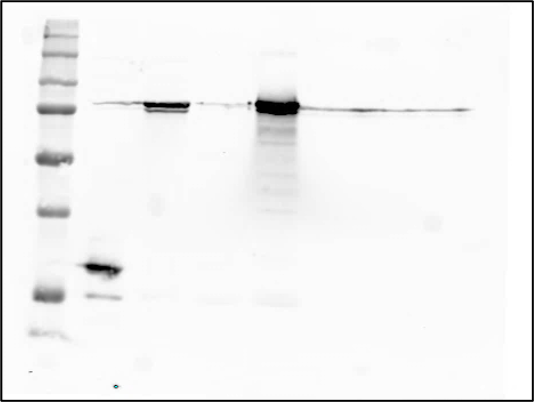

Supplement: Source data 1. [file elife-73220-data1.zip › source data (revision)/Figure 2-source data 1/Fig. 2B flag-IP GFP-IB.tif]

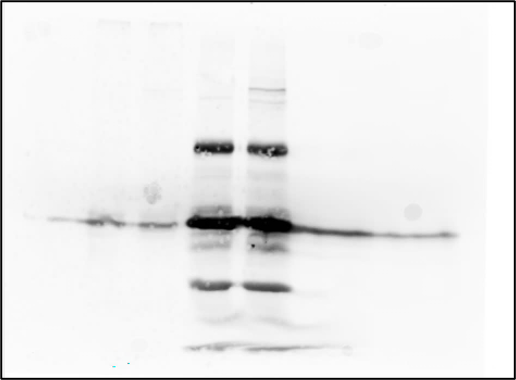

Supplement: Source data 1. [file elife-73220-data1.zip › source data (revision)/Figure 2-source data 1/Fig. 2B flag-IP flag-IB.tif]

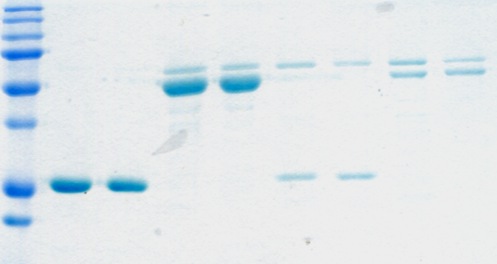

Supplement: Source data 1. [file elife-73220-data1.zip › source data (revision)/Figure 2-source data 2/Fig. 2C.tif]

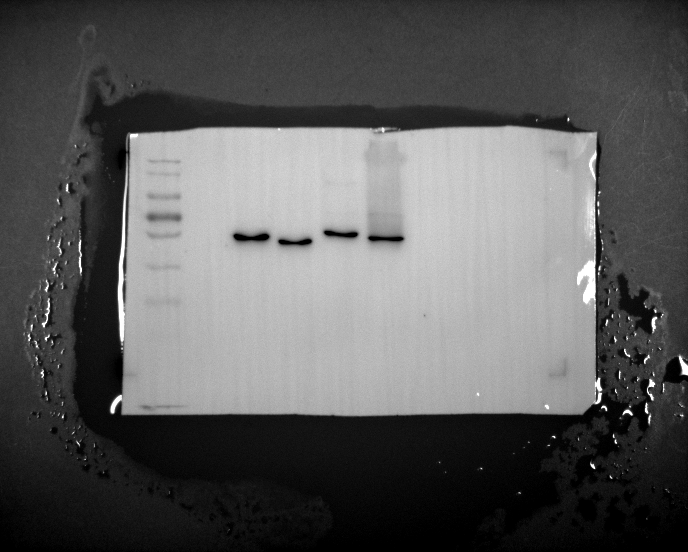

Supplement: Source data 1. [file elife-73220-data1.zip › source data (revision)/Figure 2-source data 3/Fig. 2D HA IP.Tif]

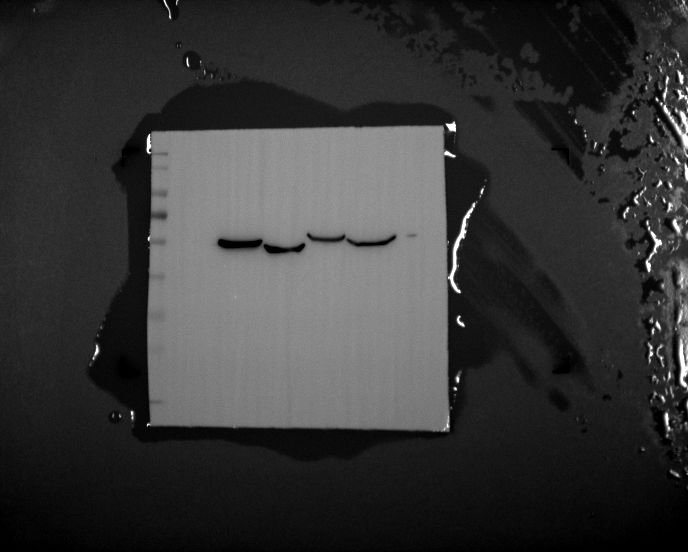

Supplement: Source data 1. [file elife-73220-data1.zip › source data (revision)/Figure 2-source data 3/Fig. 2D HA-input.Tif]

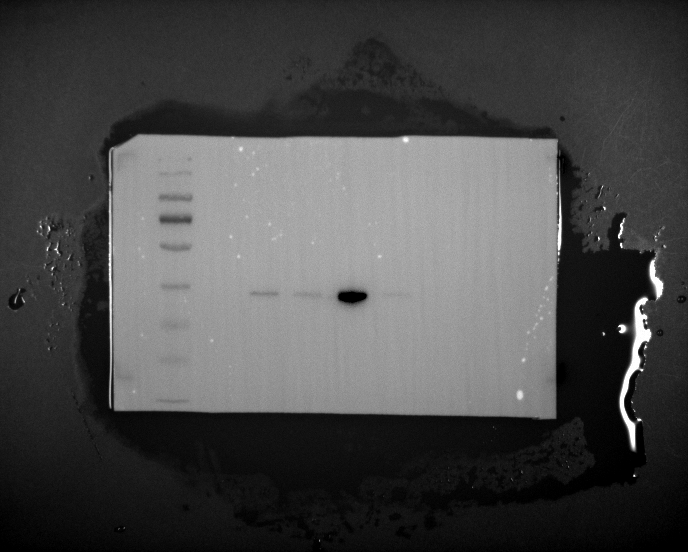

Supplement: Source data 1. [file elife-73220-data1.zip › source data (revision)/Figure 2-source data 3/Fig. 2D flag IP.Tif]

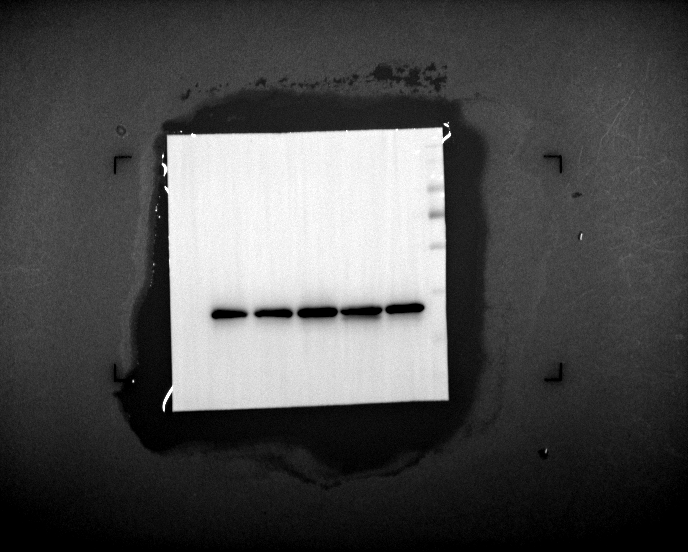

Supplement: Source data 1. [file elife-73220-data1.zip › source data (revision)/Figure 2-source data 3/Fig. 2D flag input.Tif]

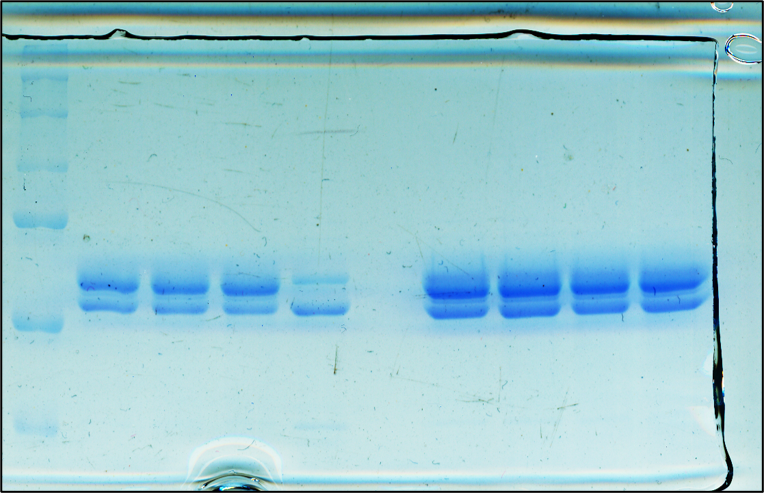

Supplement: Source data 1. [file elife-73220-data1.zip › source data (revision)/Figure 3-figure supplement 1-source data 1/Figure 3-figure supplement 1 A.tif]

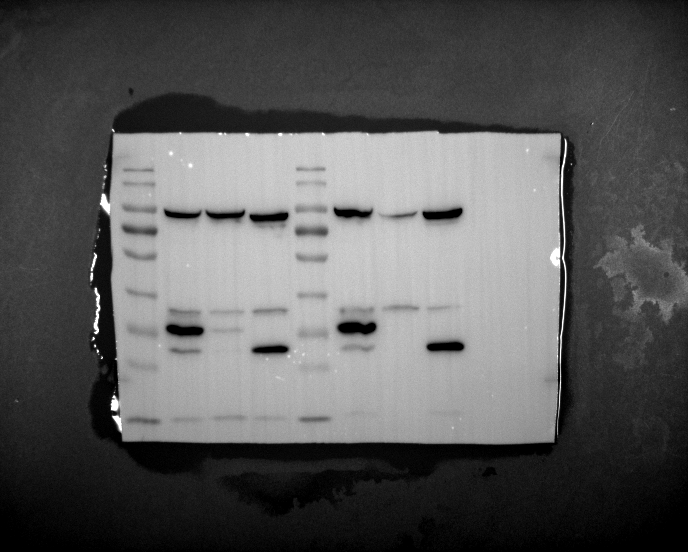

Supplement: Source data 1. [file elife-73220-data1.zip › source data (revision)/Figure 3-figure supplement 1-source data 2/Figure 3-figure supplement 1 B.Tif]

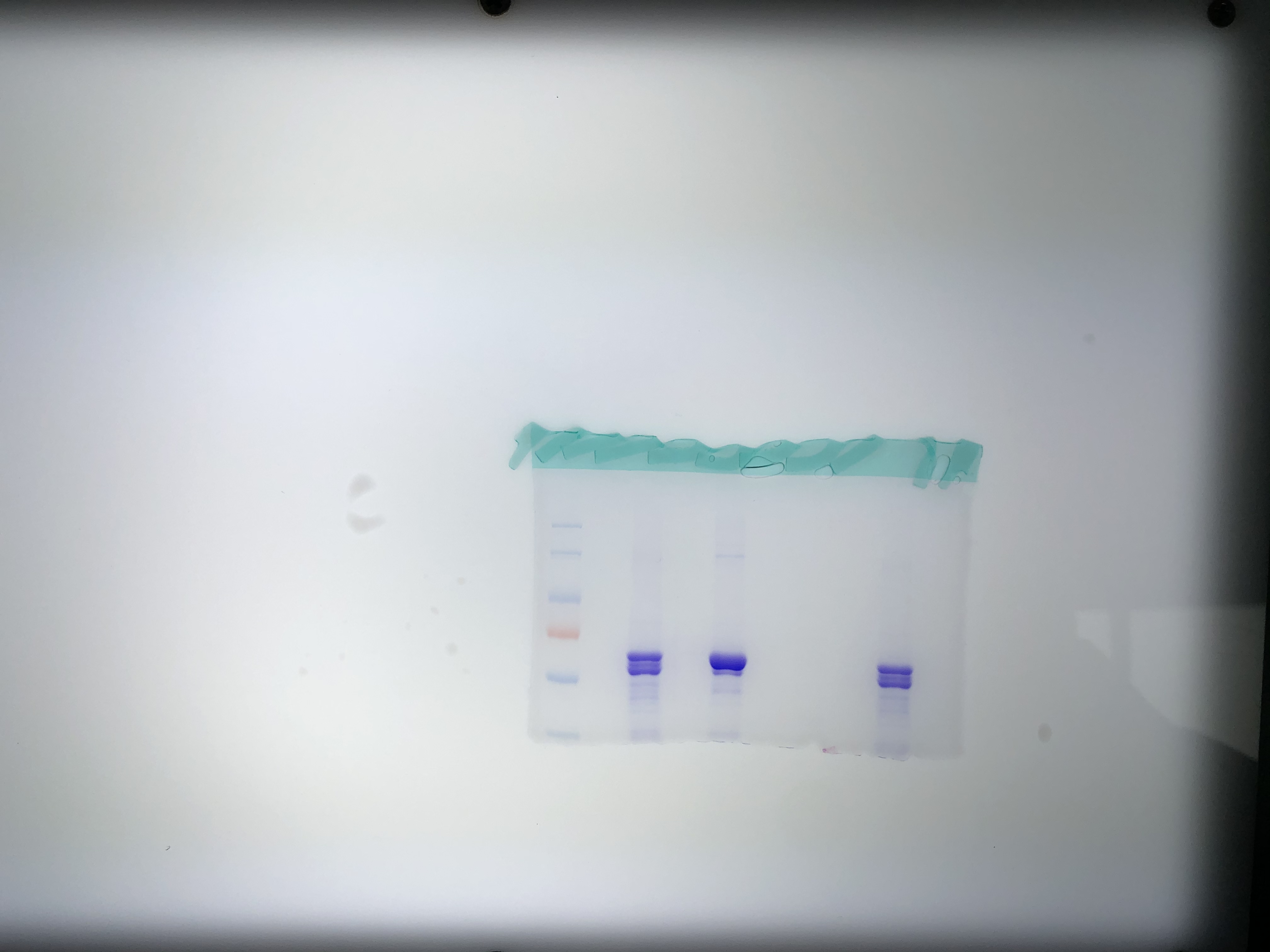

Supplement: Source data 1. [file elife-73220-data1.zip › source data (revision)/Figure 3-figure supplement 1-source data 3/Figure 3-figure supplement 1-C.jpg]

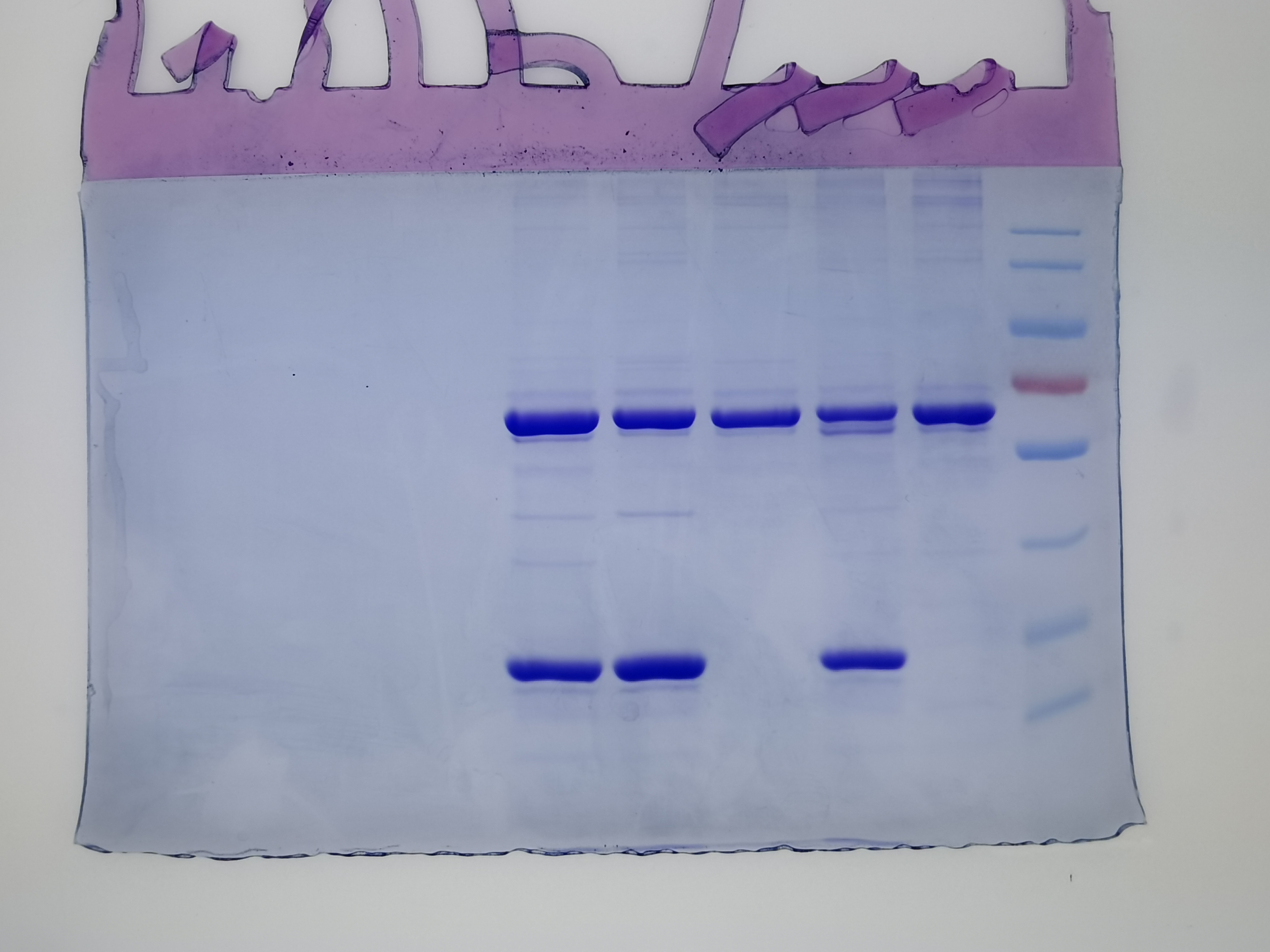

Supplement: Source data 1. [file elife-73220-data1.zip › source data (revision)/Figure 3-source data 1/Fig. 3A Lem8 autoclevage.jpg]

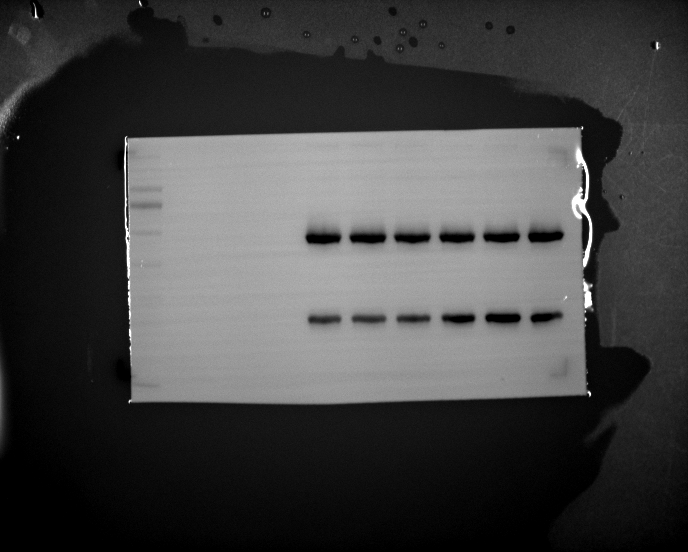

Supplement: Source data 1. [file elife-73220-data1.zip › source data (revision)/Figure 3-source data 2/Fig. 3B GST marker.Tif]

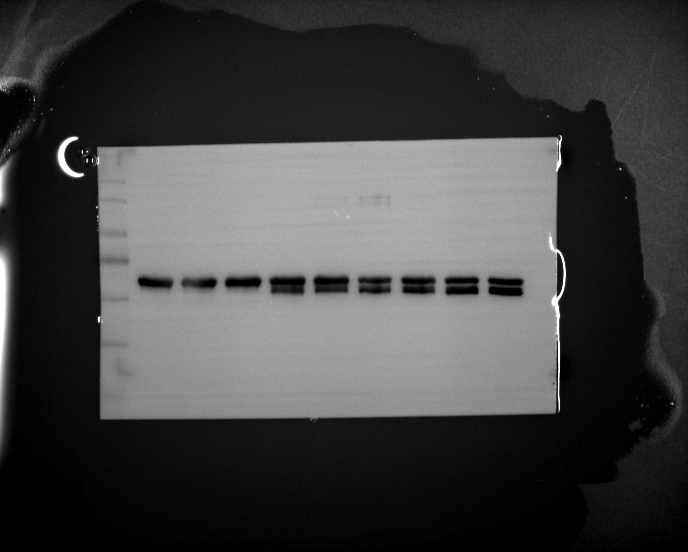

Supplement: Source data 1. [file elife-73220-data1.zip › source data (revision)/Figure 3-source data 2/Fig. 3B His marker.Tif]

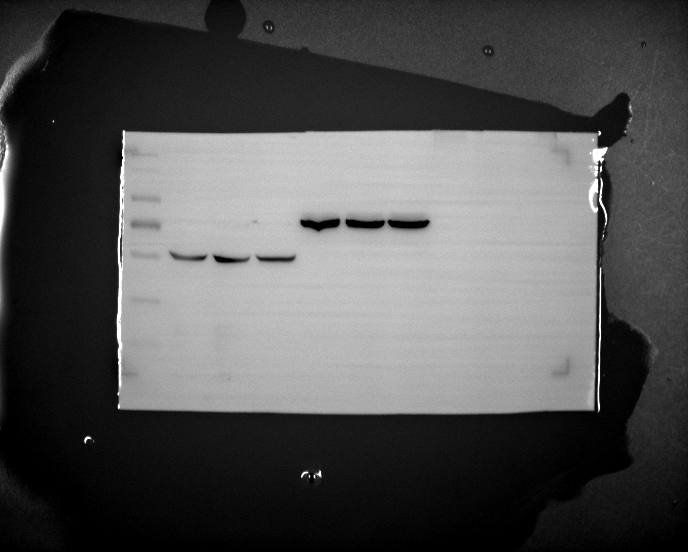

Supplement: Source data 1. [file elife-73220-data1.zip › source data (revision)/Figure 4-figure supplement 1-source data 1/Ankrd13B.Tif]

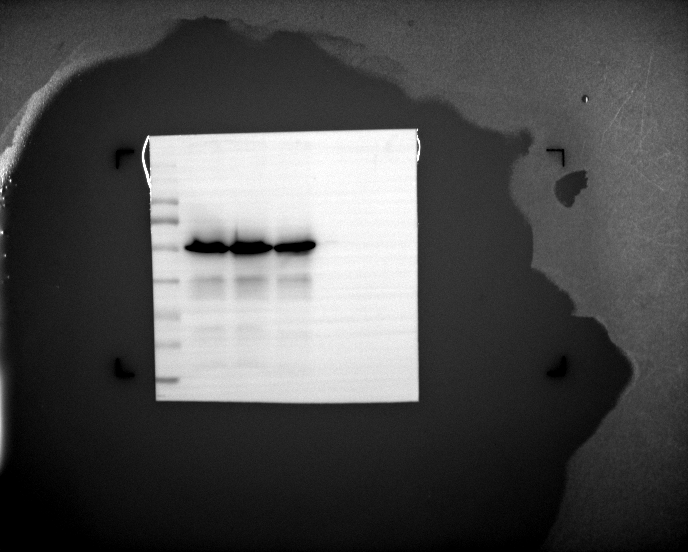

Supplement: Source data 1. [file elife-73220-data1.zip › source data (revision)/Figure 4-figure supplement 1-source data 1/ChkB.Tif]

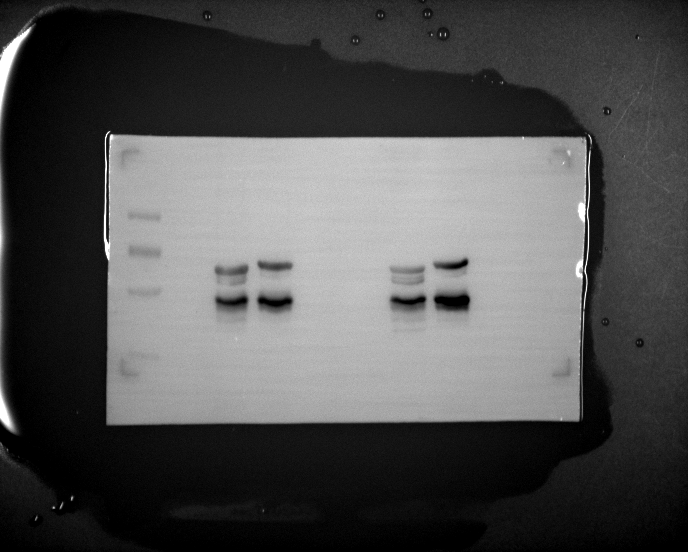

Supplement: Source data 1. [file elife-73220-data1.zip › source data (revision)/Figure 4-figure supplement 1-source data 1/GST1.Tif]

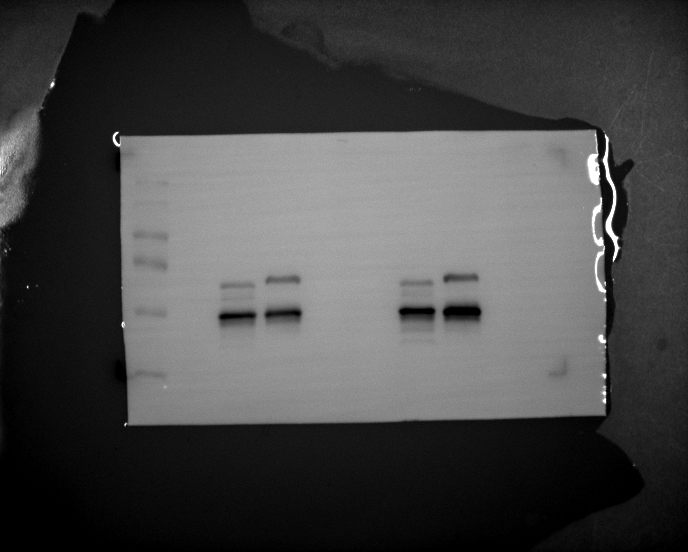

Supplement: Source data 1. [file elife-73220-data1.zip › source data (revision)/Figure 4-figure supplement 1-source data 1/GST2.Tif]

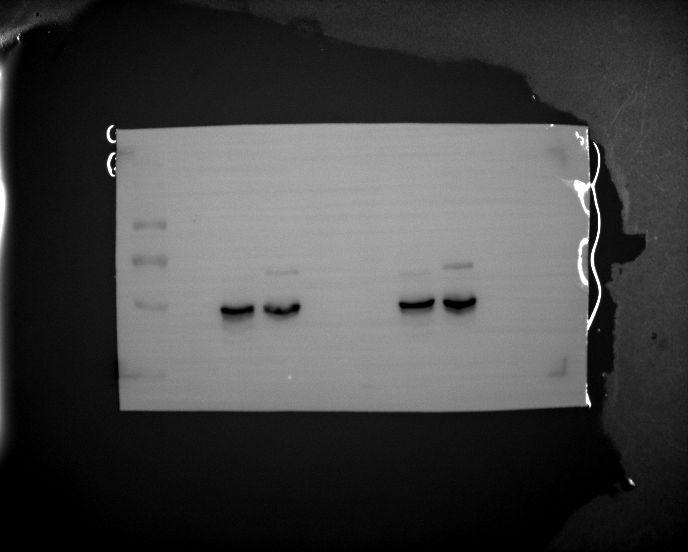

Supplement: Source data 1. [file elife-73220-data1.zip › source data (revision)/Figure 4-figure supplement 1-source data 1/GST3.Tif]

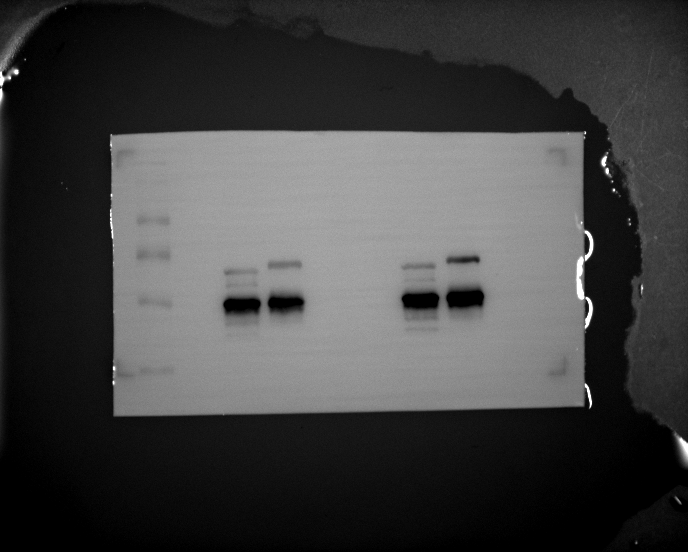

Supplement: Source data 1. [file elife-73220-data1.zip › source data (revision)/Figure 4-figure supplement 1-source data 1/GST4.Tif]

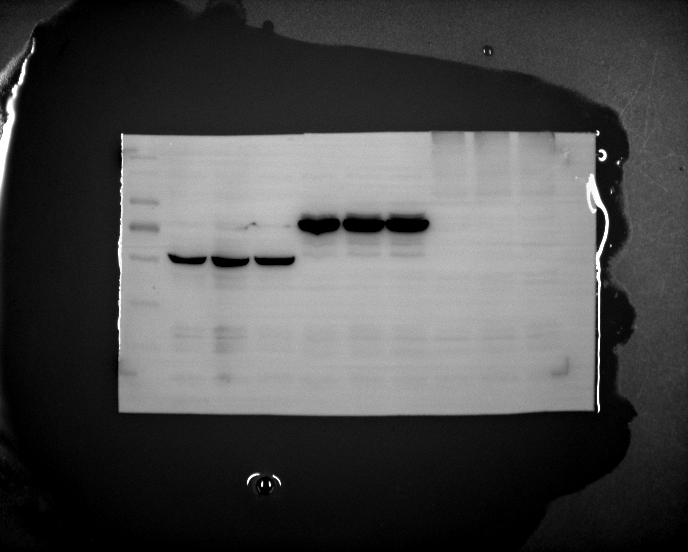

Supplement: Source data 1. [file elife-73220-data1.zip › source data (revision)/Figure 4-figure supplement 1-source data 1/Gnal.Tif]

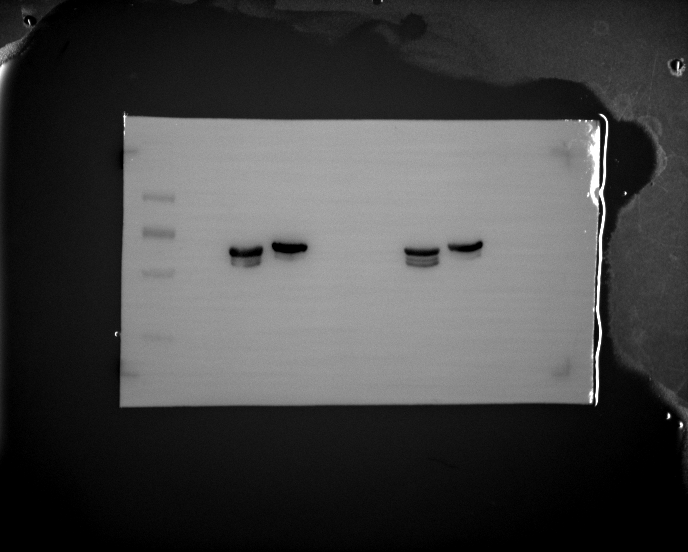

Supplement: Source data 1. [file elife-73220-data1.zip › source data (revision)/Figure 4-figure supplement 1-source data 1/HIS1.Tif]

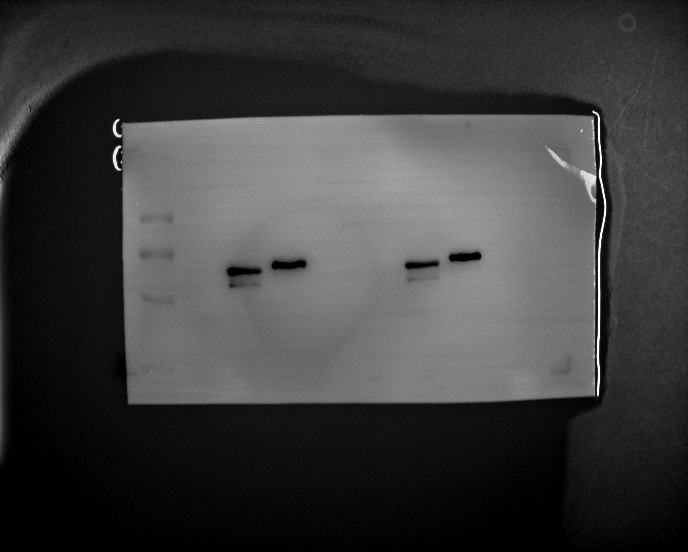

Supplement: Source data 1. [file elife-73220-data1.zip › source data (revision)/Figure 4-figure supplement 1-source data 1/HIS2.Tif]

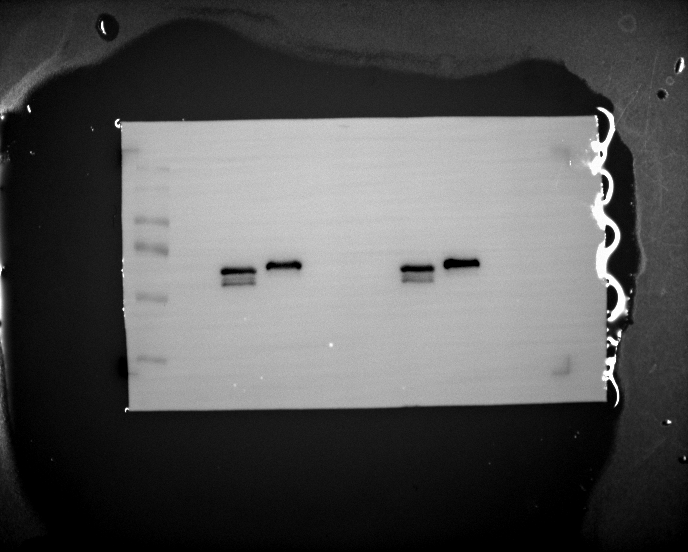

Supplement: Source data 1. [file elife-73220-data1.zip › source data (revision)/Figure 4-figure supplement 1-source data 1/HIS3.Tif]

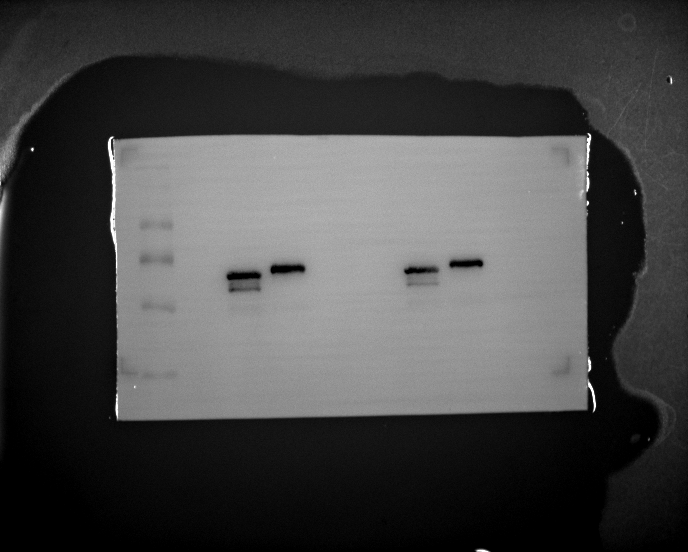

Supplement: Source data 1. [file elife-73220-data1.zip › source data (revision)/Figure 4-figure supplement 1-source data 1/HIS4.Tif]

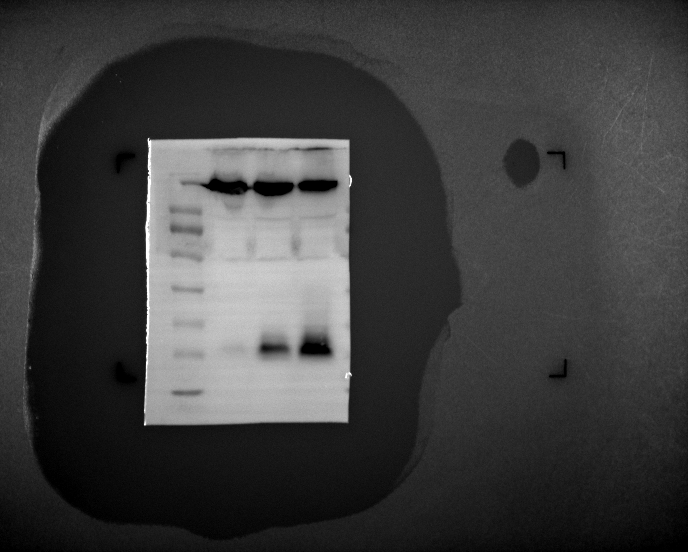

Supplement: Source data 1. [file elife-73220-data1.zip › source data (revision)/Figure 4-figure supplement 1-source data 1/KIAA1033.Tif]

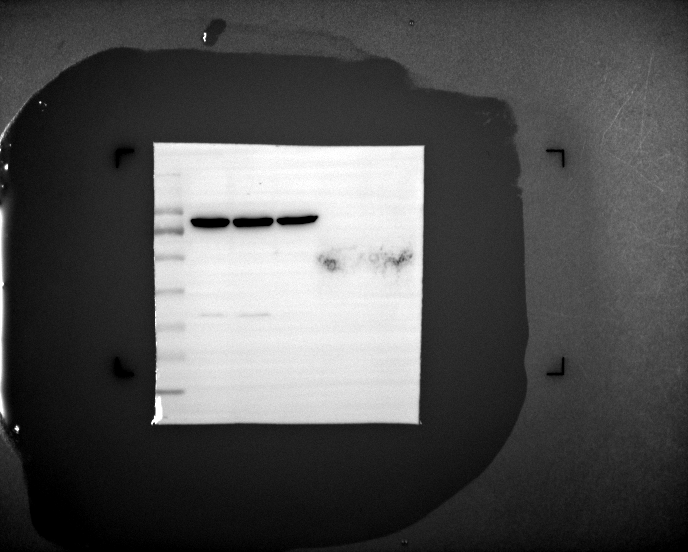

Supplement: Source data 1. [file elife-73220-data1.zip › source data (revision)/Figure 4-figure supplement 1-source data 1/Pak6.Tif]

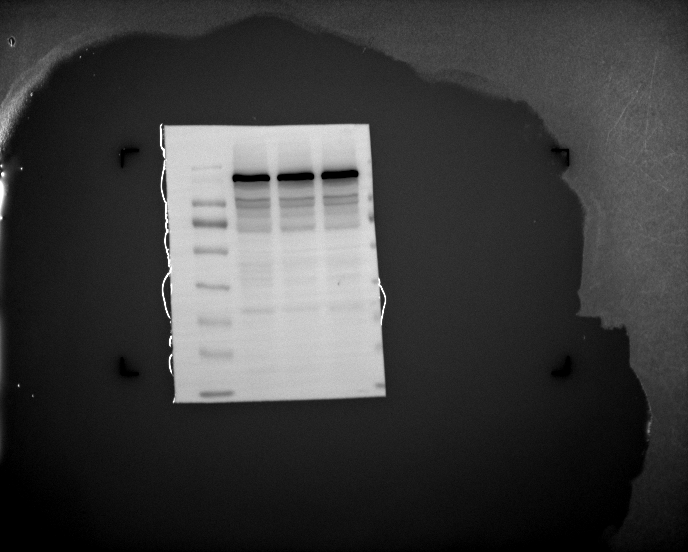

Supplement: Source data 1. [file elife-73220-data1.zip › source data (revision)/Figure 4-figure supplement 1-source data 1/Ppp6R1.Tif]

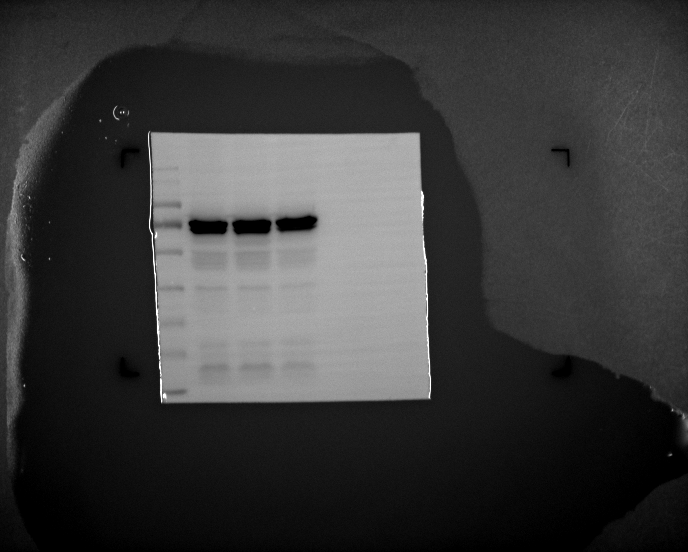

Supplement: Source data 1. [file elife-73220-data1.zip › source data (revision)/Figure 4-figure supplement 1-source data 1/RASGRP2.Tif]

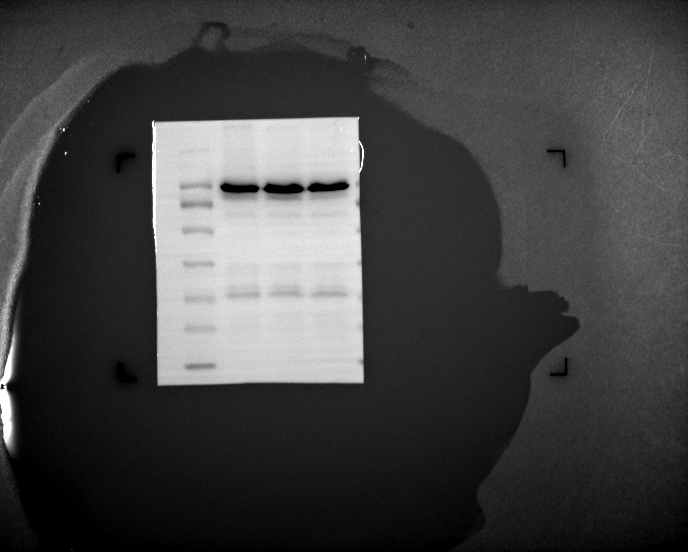

Supplement: Source data 1. [file elife-73220-data1.zip › source data (revision)/Figure 4-figure supplement 1-source data 1/exoc8.Tif]

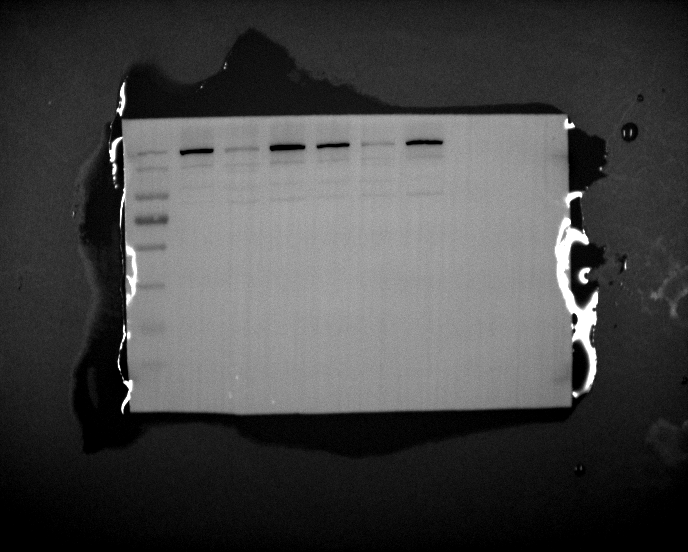

Supplement: Source data 1. [file elife-73220-data1.zip › source data (revision)/Figure 4-figure supplement 1-source data 3/Figure 4-figure supplement 1-C Flag.Tif]

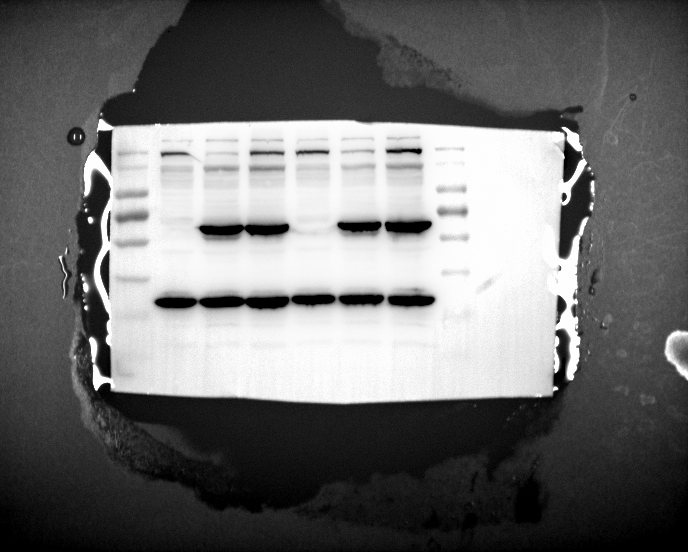

Supplement: Source data 1. [file elife-73220-data1.zip › source data (revision)/Figure 4-figure supplement 1-source data 3/Figure 4-figure supplement 1-C HA.Tif]

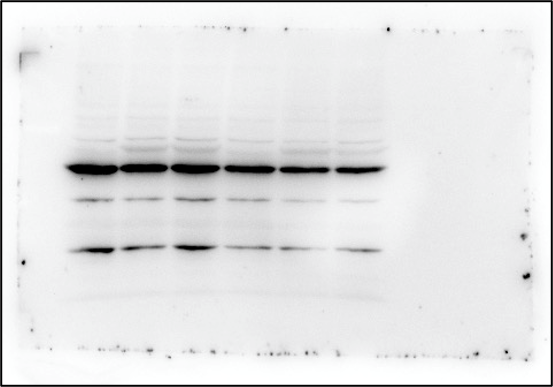

Supplement: Source data 1. [file elife-73220-data1.zip › source data (revision)/Figure 4-figure supplement 1-source data 3/Figure 4-figure supplement 1-C Tubulin.tif]

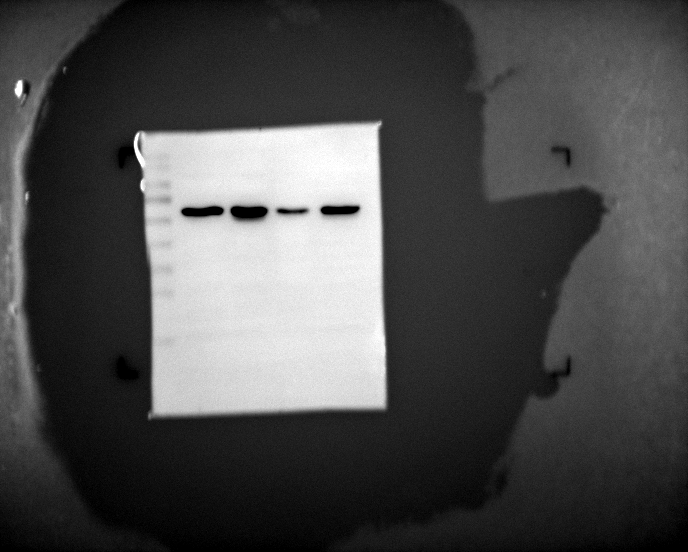

Supplement: Source data 1. [file elife-73220-data1.zip › source data (revision)/Figure 4-figure supplement 1-source data 4/Figure 4-figure supplement 1-D C Lem8.Tif]

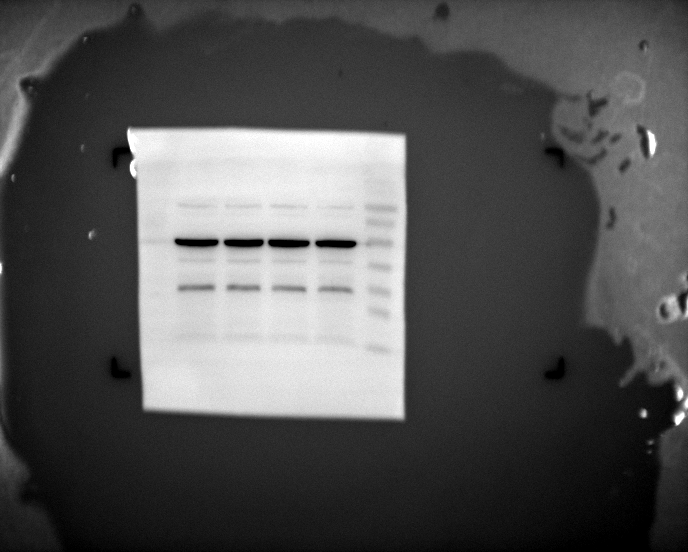

Supplement: Source data 1. [file elife-73220-data1.zip › source data (revision)/Figure 4-figure supplement 1-source data 4/Figure 4-figure supplement 1-D C Tubulin.Tif]

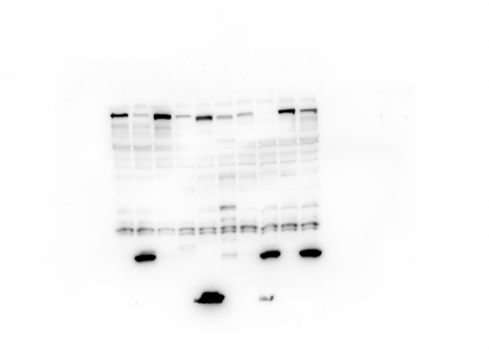

Supplement: Source data 1. [file elife-73220-data1.zip › source data (revision)/Figure 4-figure supplement 1-source data 4/Figure 4-figure supplement 1-D C cleaved GFP.tif]

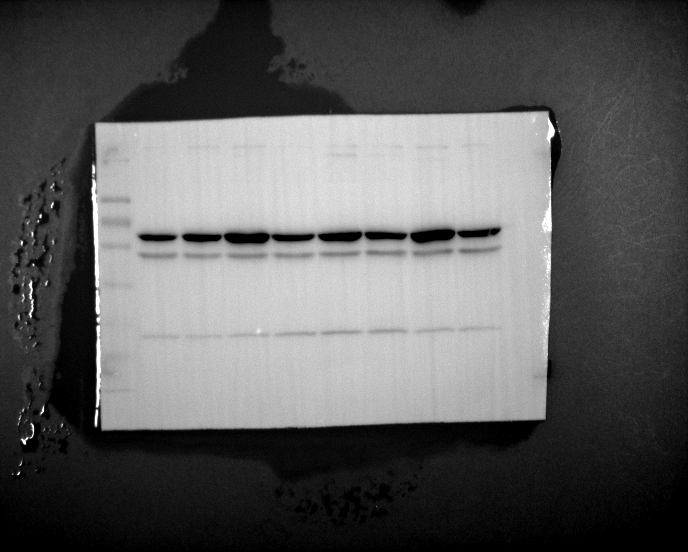

Supplement: Source data 1. [file elife-73220-data1.zip › source data (revision)/Figure 4-figure supplement 1-source data 4/Figure 4-figure supplement 1-D N Lem8.Tif]

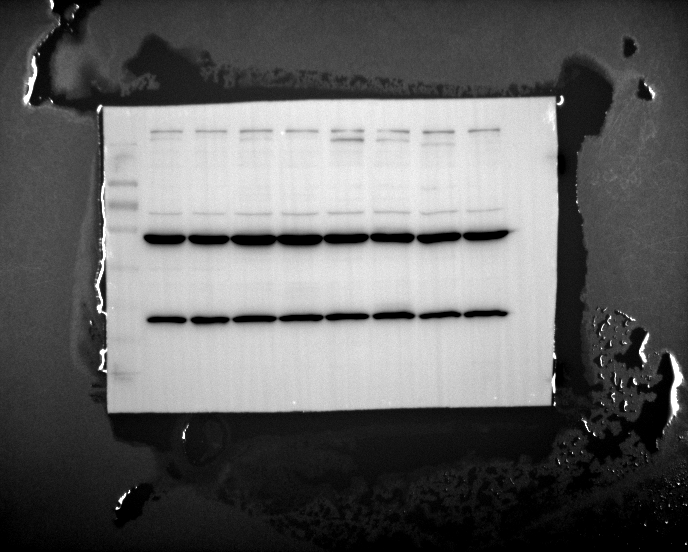

Supplement: Source data 1. [file elife-73220-data1.zip › source data (revision)/Figure 4-figure supplement 1-source data 4/Figure 4-figure supplement 1-D N Tubulin.Tif]

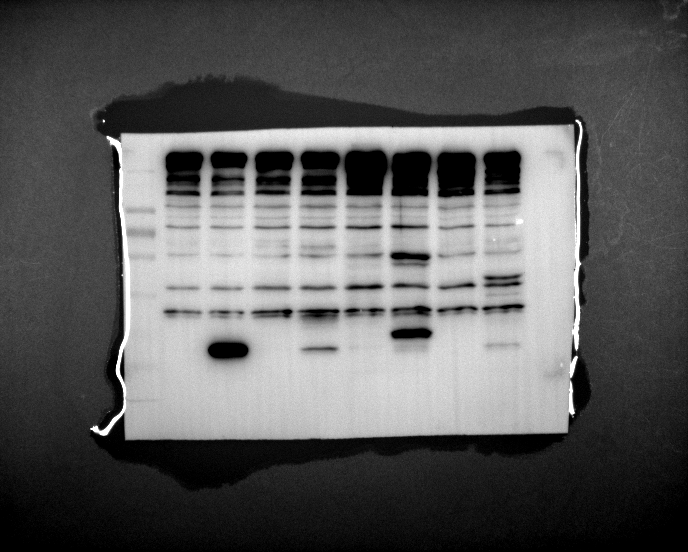

Supplement: Source data 1. [file elife-73220-data1.zip › source data (revision)/Figure 4-figure supplement 1-source data 4/Figure 4-figure supplement 1-D N cleaved GFP.Tif]

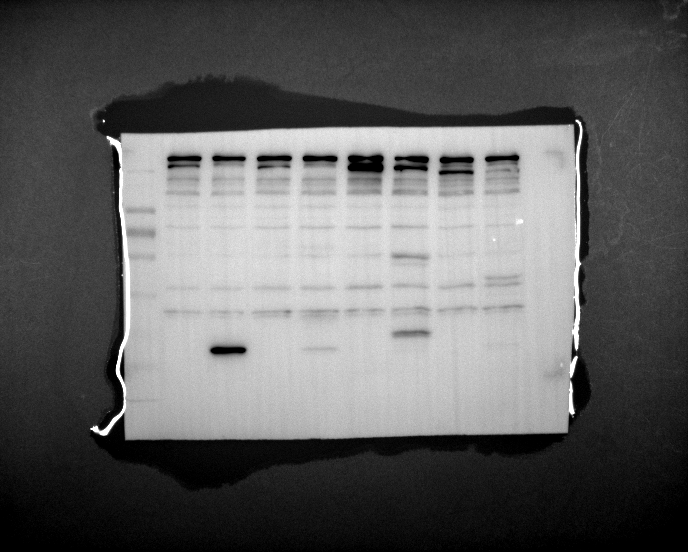

Supplement: Source data 1. [file elife-73220-data1.zip › source data (revision)/Figure 4-figure supplement 1-source data 4/Figure 4-figure supplement 1-D N phldb2.Tif]

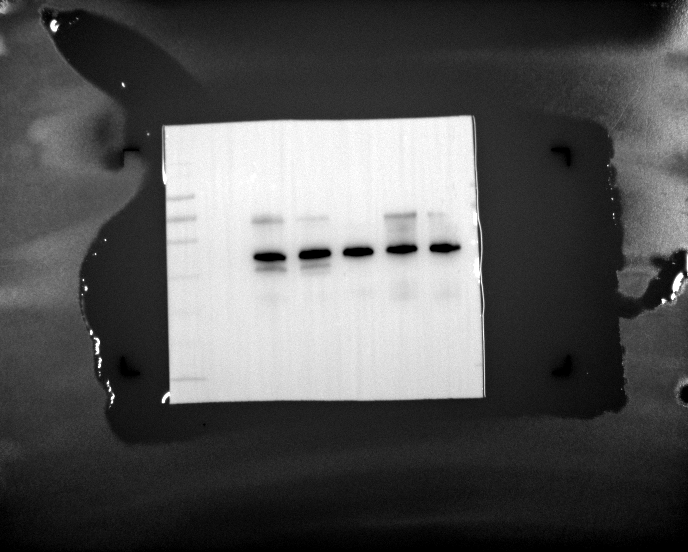

Supplement: Source data 1. [file elife-73220-data1.zip › source data (revision)/Figure 4-figure supplement 1-source data 5/Figure 4-figure supplement 1-E ICDH.Tif]

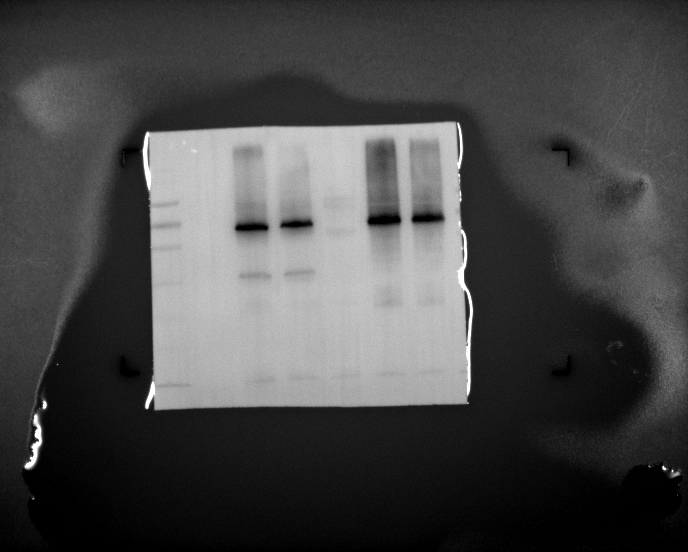

Supplement: Source data 1. [file elife-73220-data1.zip › source data (revision)/Figure 4-figure supplement 1-source data 5/Figure 4-figure supplement 1-E Lem8 expression.Tif]

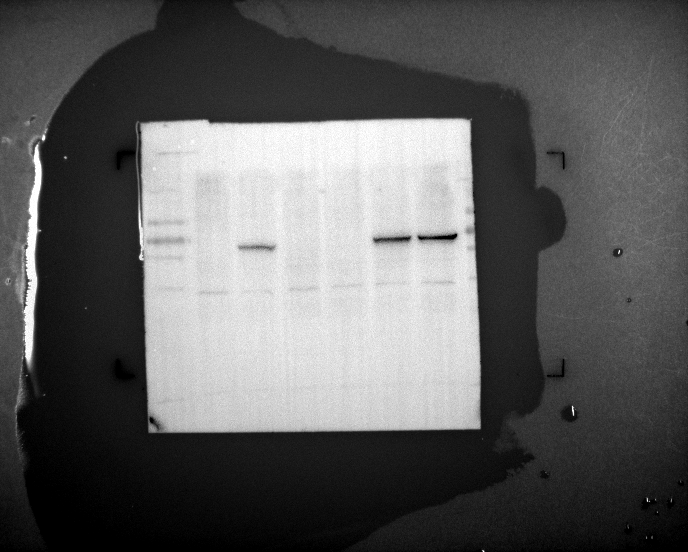

Supplement: Source data 1. [file elife-73220-data1.zip › source data (revision)/Figure 4-figure supplement 1-source data 5/Figure 4-figure supplement 1-E Lem8 translocation.Tif]

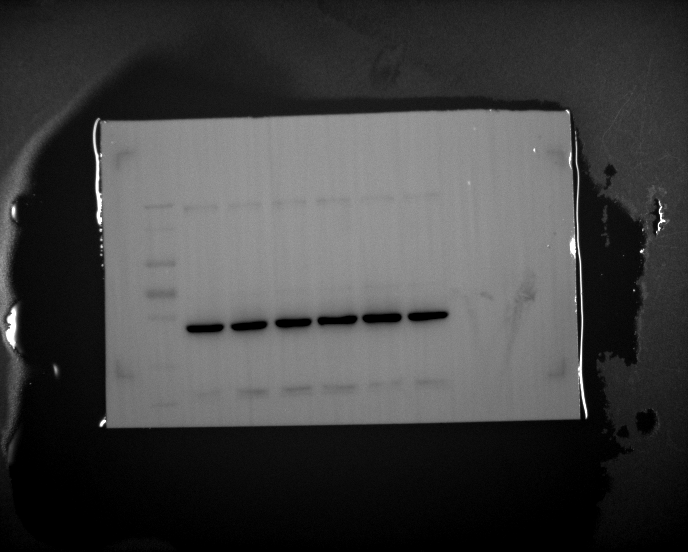

Supplement: Source data 1. [file elife-73220-data1.zip › source data (revision)/Figure 4-figure supplement 1-source data 5/Figure 4-figure supplement 1-E Tubulin.Tif]

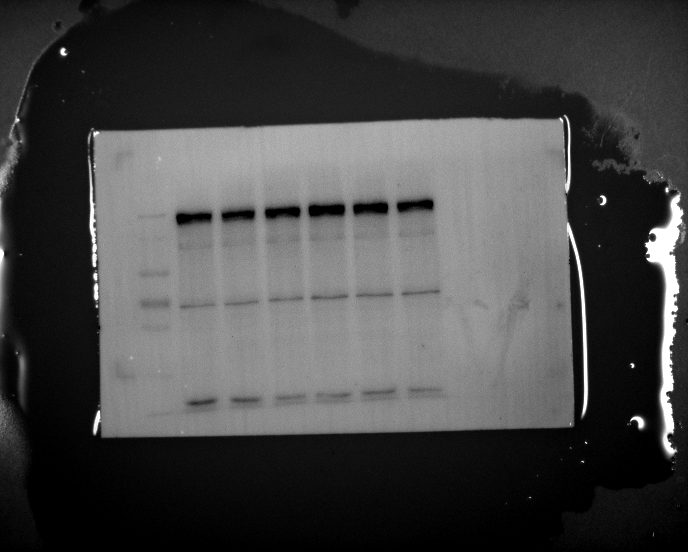

Supplement: Source data 1. [file elife-73220-data1.zip › source data (revision)/Figure 4-figure supplement 1-source data 5/Figure 4-figure supplement 1-E phldb2.Tif]

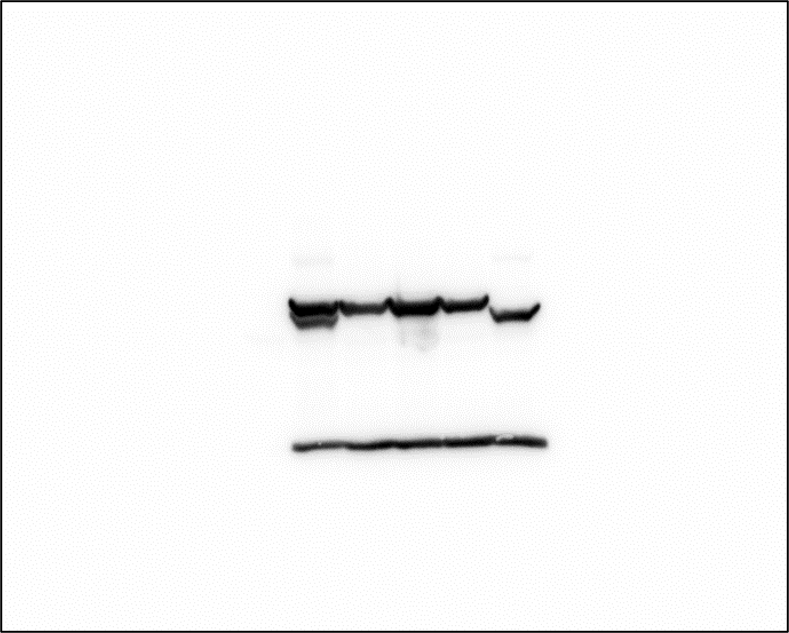

Supplement: Source data 1. [file elife-73220-data1.zip › source data (revision)/Figure 4-source data 1/Fig. 4B Lem8.tif]

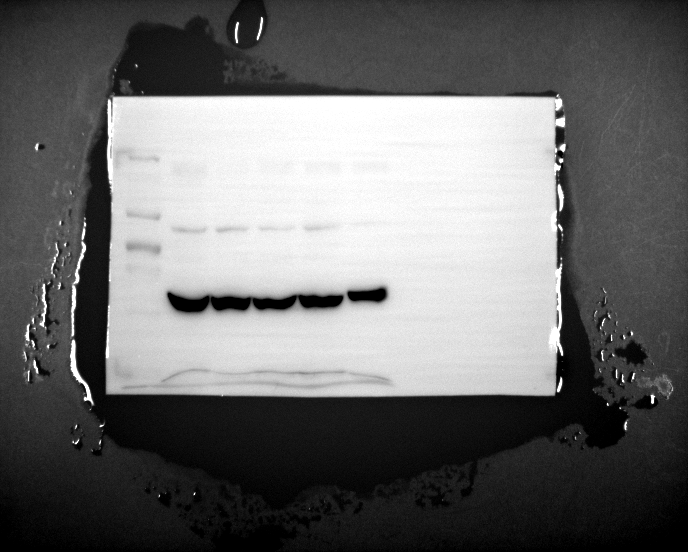

Supplement: Source data 1. [file elife-73220-data1.zip › source data (revision)/Figure 4-source data 1/Fig. 4B Tubulin.Tif]

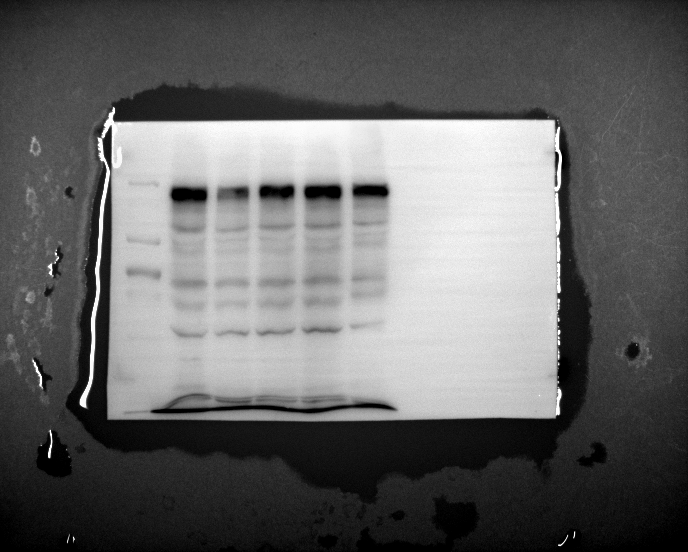

Supplement: Source data 1. [file elife-73220-data1.zip › source data (revision)/Figure 4-source data 1/Fig. 4B phldb2.Tif]

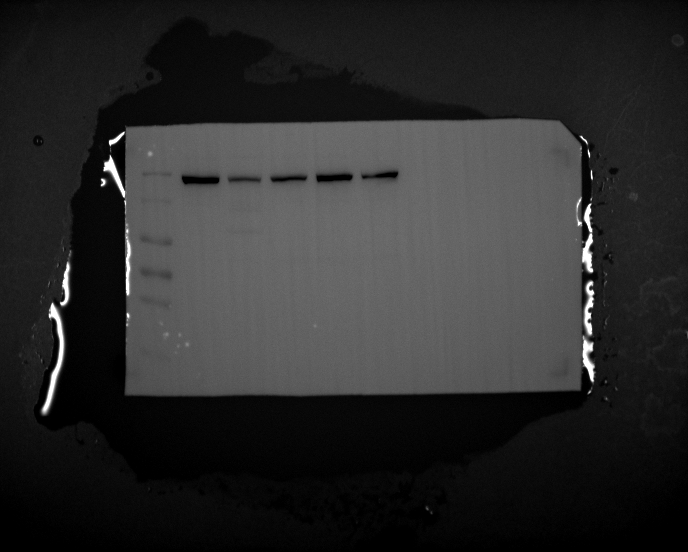

Supplement: Source data 1. [file elife-73220-data1.zip › source data (revision)/Figure 4-source data 2/Fig. 4C Flag.Tif]

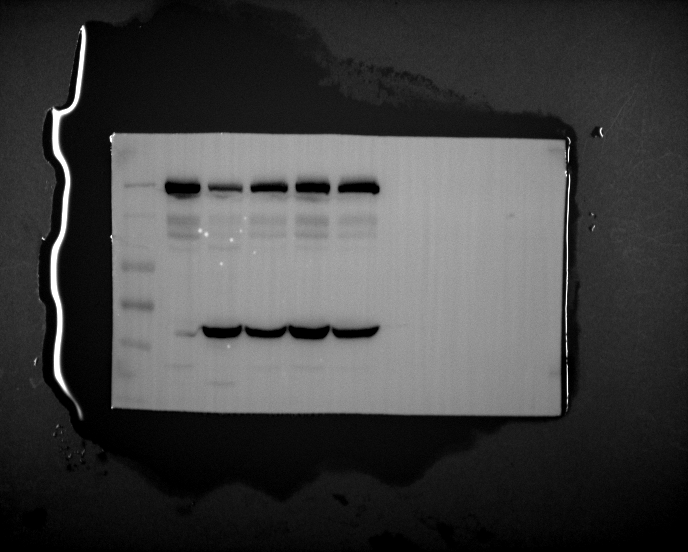

Supplement: Source data 1. [file elife-73220-data1.zip › source data (revision)/Figure 4-source data 2/Fig. 4C HA.Tif]

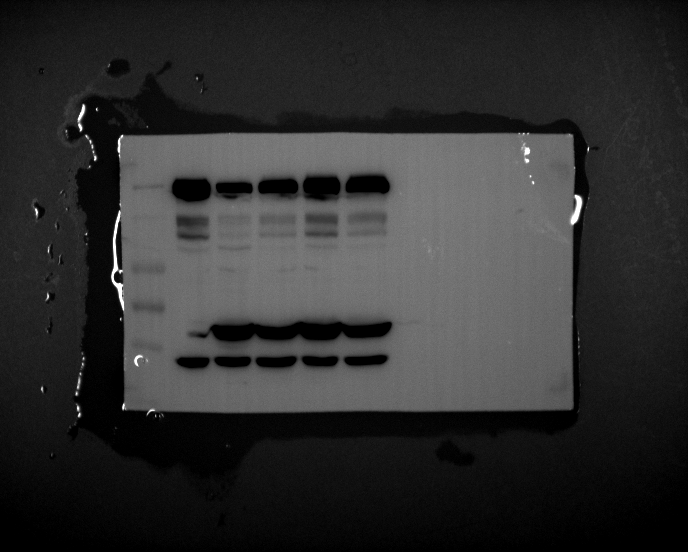

Supplement: Source data 1. [file elife-73220-data1.zip › source data (revision)/Figure 4-source data 2/Fig. 4C Tubulin.Tif]

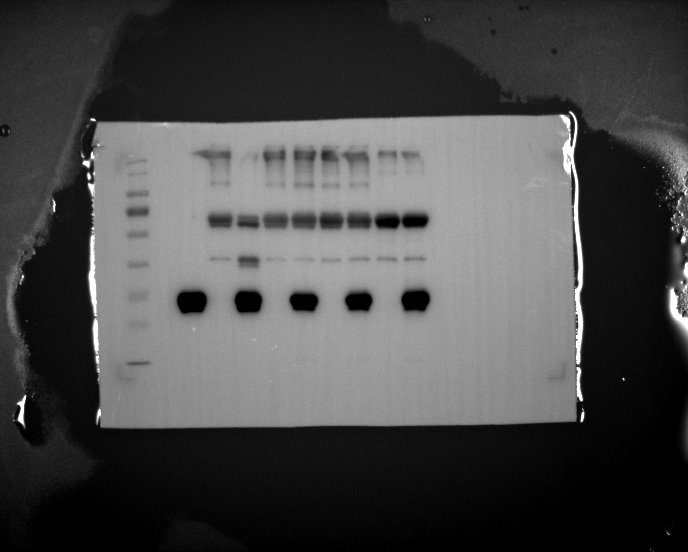

Supplement: Source data 1. [file elife-73220-data1.zip › source data (revision)/Figure 4-source data 4/Fig. 4E 14-3-3.Tif]

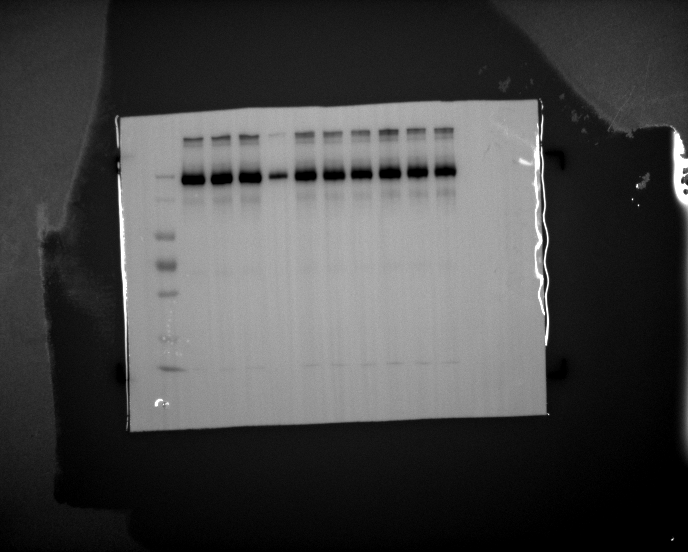

Supplement: Source data 1. [file elife-73220-data1.zip › source data (revision)/Figure 4-source data 4/Fig. 4E HA.Tif]

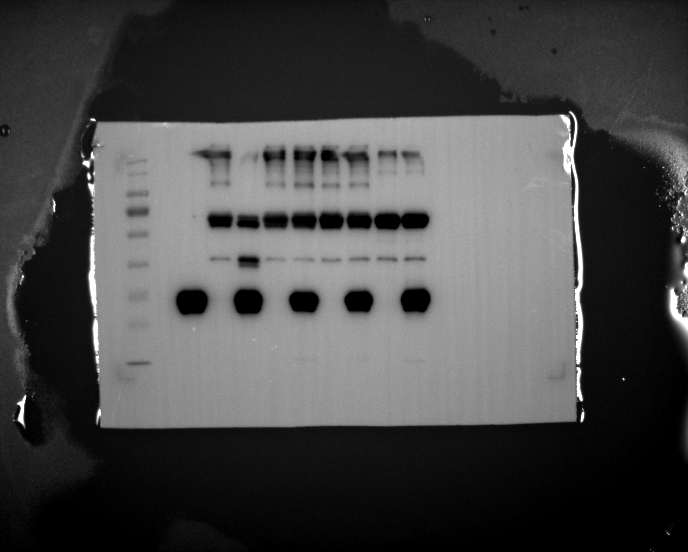

Supplement: Source data 1. [file elife-73220-data1.zip › source data (revision)/Figure 4-source data 4/Fig. 4E Lem8.Tif]

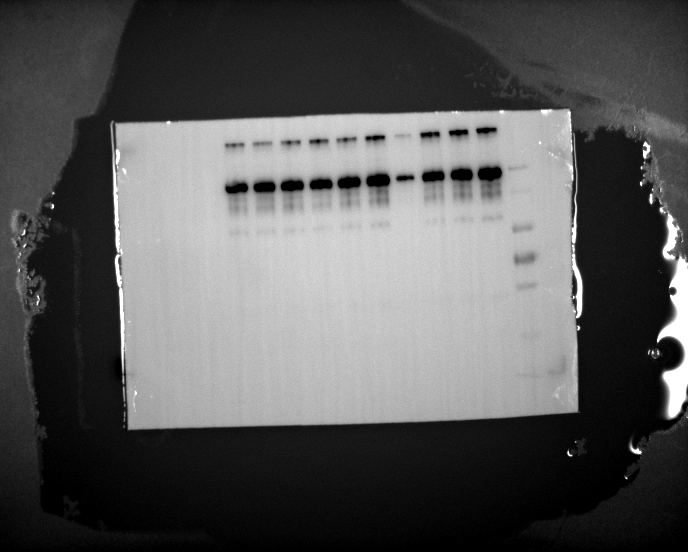

Supplement: Source data 1. [file elife-73220-data1.zip › source data (revision)/Figure 4-source data 4/Fig. 4E flag.Tif]

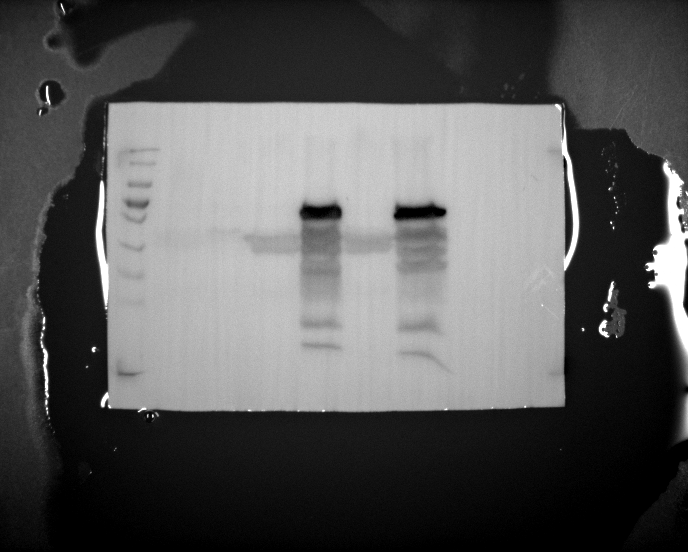

Supplement: Source data 1. [file elife-73220-data1.zip › source data (revision)/Figure 5-source data 1/Fig. 5B Lem8.Tif]

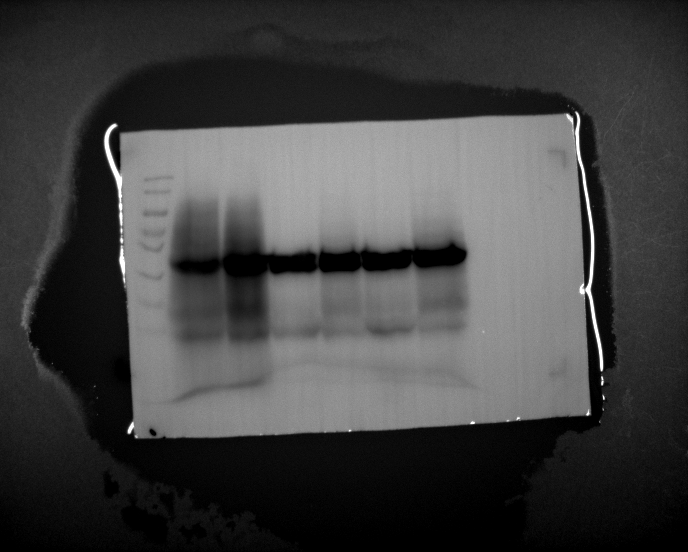

Supplement: Source data 1. [file elife-73220-data1.zip › source data (revision)/Figure 5-source data 1/Fig. 5B PGKalpha.Tif]

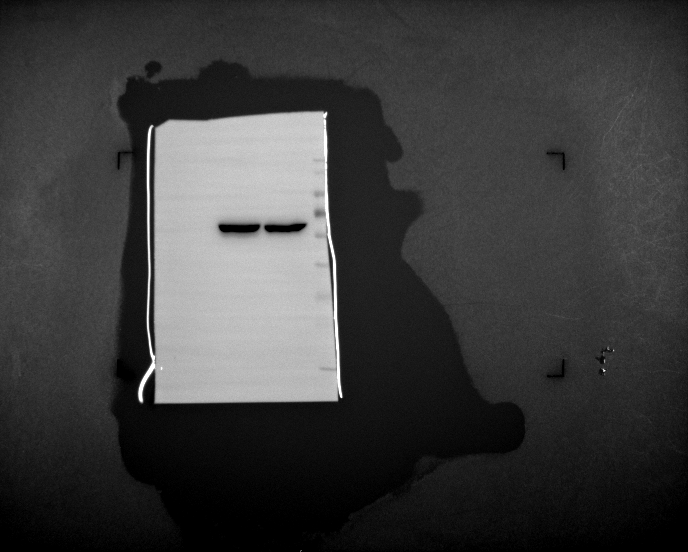

Supplement: Source data 1. [file elife-73220-data1.zip › source data (revision)/Figure 5-source data 2/Fig. 5C HA.Tif]

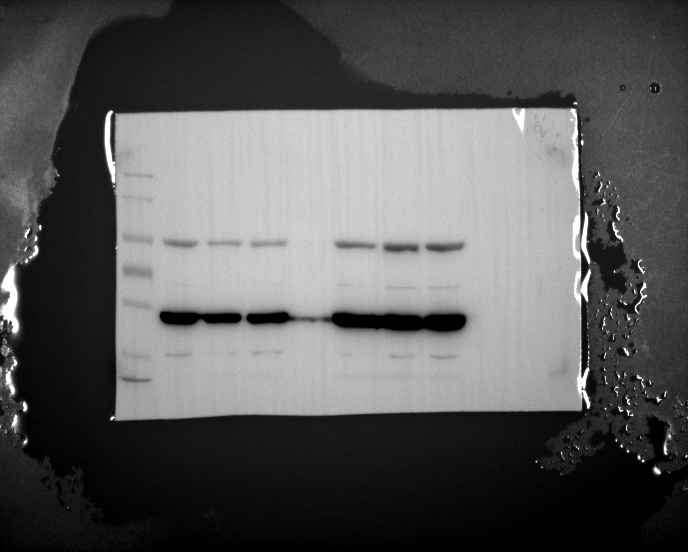

Supplement: Source data 1. [file elife-73220-data1.zip › source data (revision)/Figure 5-source data 2/Fig. 5C Tubulin.Tif]

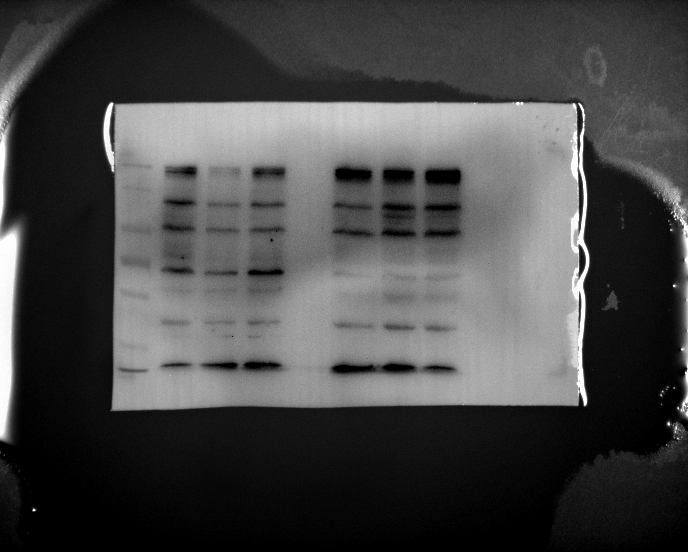

Supplement: Source data 1. [file elife-73220-data1.zip › source data (revision)/Figure 5-source data 2/Fig. 5C phldb2.Tif]

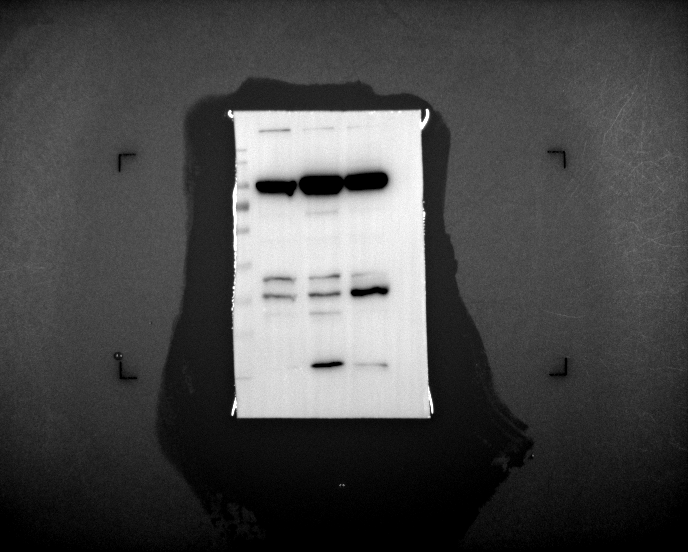

Supplement: Source data 1. [file elife-73220-data1.zip › source data (revision)/Figure 5-source data 3/Fig. 5D.Tif]

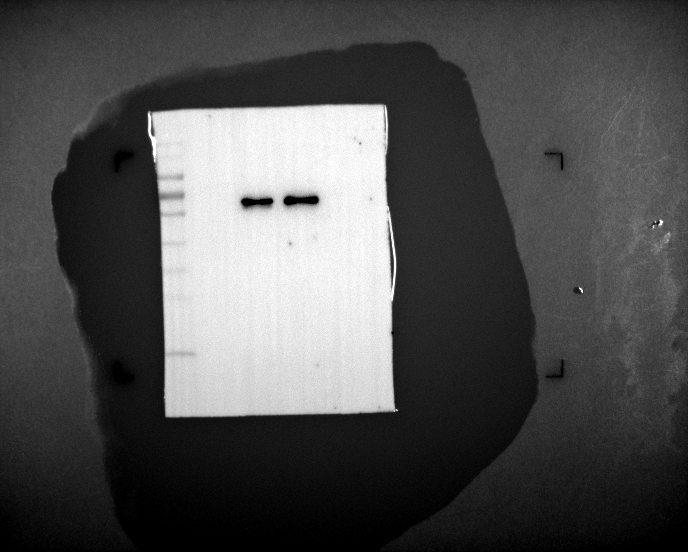

Supplement: Source data 1. [file elife-73220-data1.zip › source data (revision)/Figure 5-source data 4/Fig. 5E IP HA.Tif]

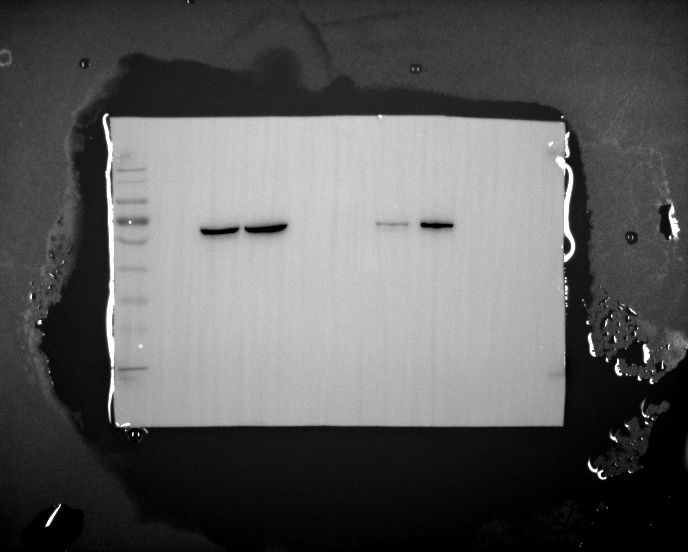

Supplement: Source data 1. [file elife-73220-data1.zip › source data (revision)/Figure 5-source data 4/Fig. 5E Input HA.Tif]

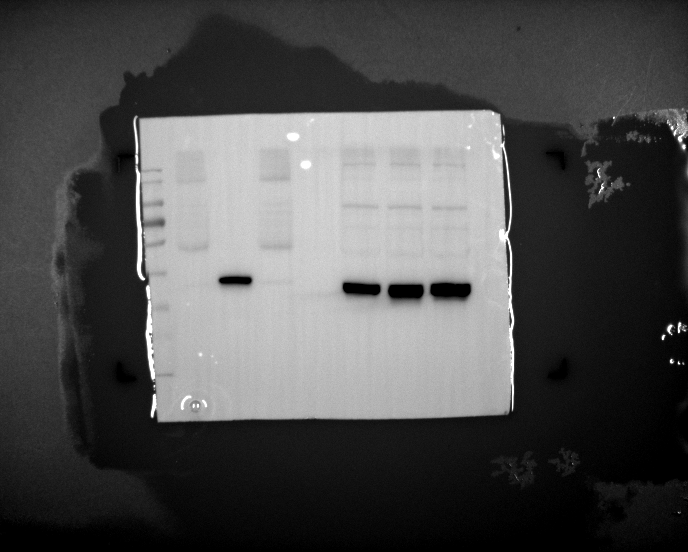

Supplement: Source data 1. [file elife-73220-data1.zip › source data (revision)/Figure 5-source data 4/Fig. 5E flag.Tif]

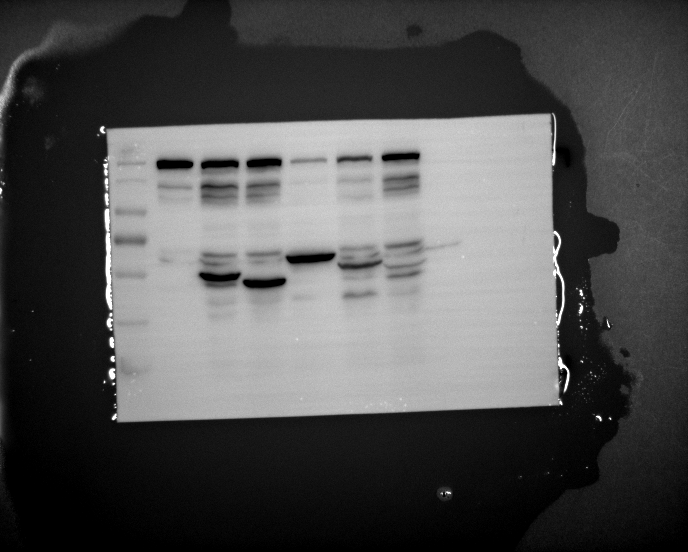

Supplement: Source data 1. [file elife-73220-data1.zip › source data (revision)/Figure 6-source data 1/Fig. 6A HA.Tif]

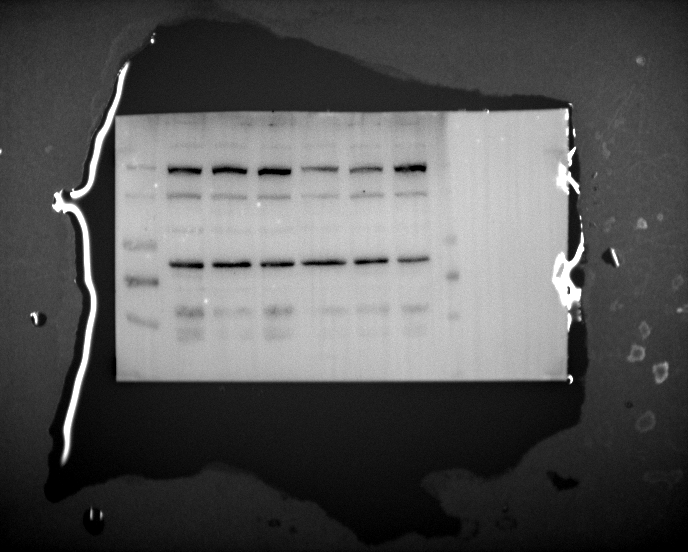

Supplement: Source data 1. [file elife-73220-data1.zip › source data (revision)/Figure 6-source data 1/Fig. 6A flag.Tif]

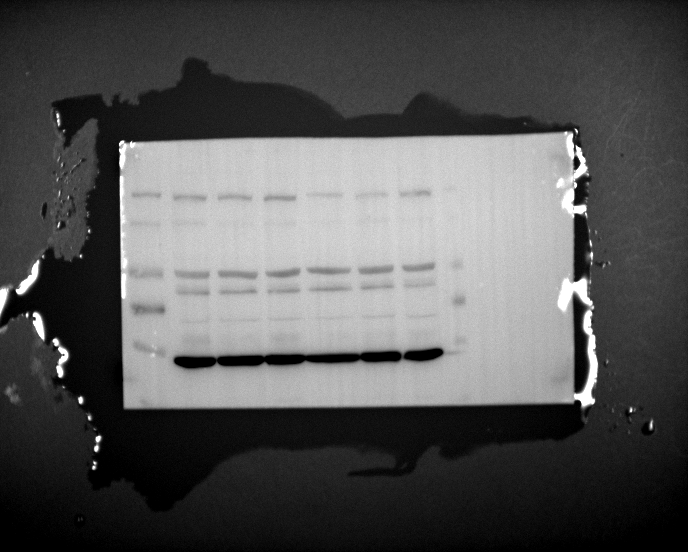

Supplement: Source data 1. [file elife-73220-data1.zip › source data (revision)/Figure 6-source data 1/Fig. 6A tubulin.Tif]

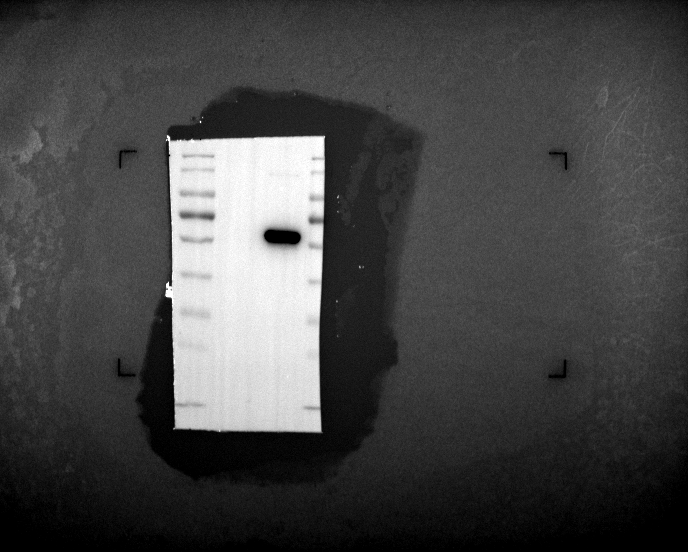

Supplement: Source data 1. [file elife-73220-data1.zip › source data (revision)/Figure 6-source data 3/Fig. 6C IP-HA IB-HA.Tif]

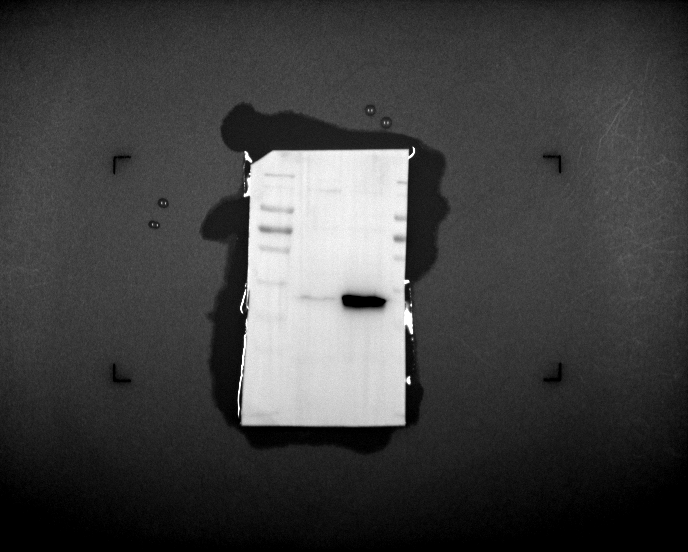

Supplement: Source data 1. [file elife-73220-data1.zip › source data (revision)/Figure 6-source data 3/Fig. 6C IP-HA IB-flag.Tif]

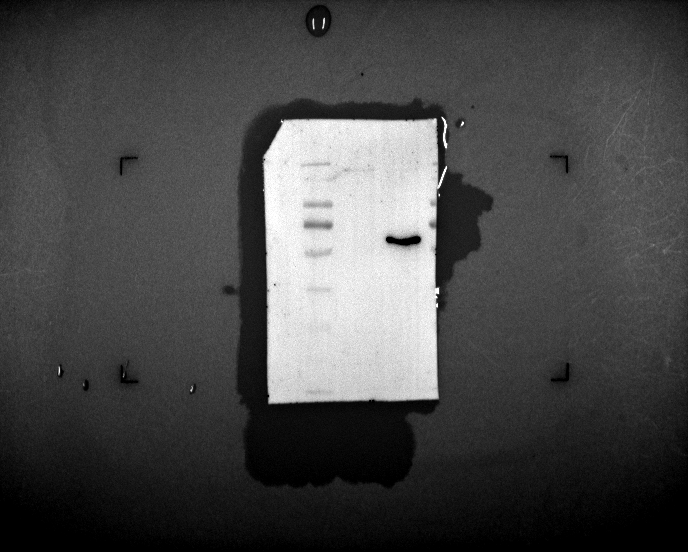

Supplement: Source data 1. [file elife-73220-data1.zip › source data (revision)/Figure 6-source data 3/Fig. 6C IP-flag IB-HA.Tif]

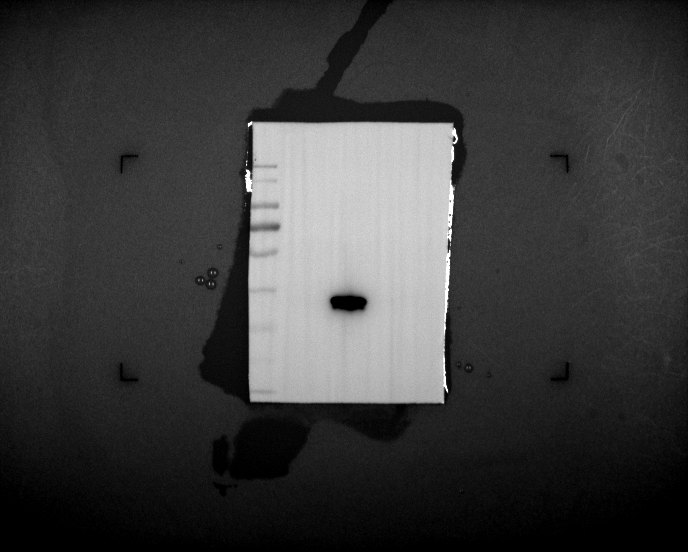

Supplement: Source data 1. [file elife-73220-data1.zip › source data (revision)/Figure 6-source data 3/Fig. 6C IP-flag IB-flag.Tif]

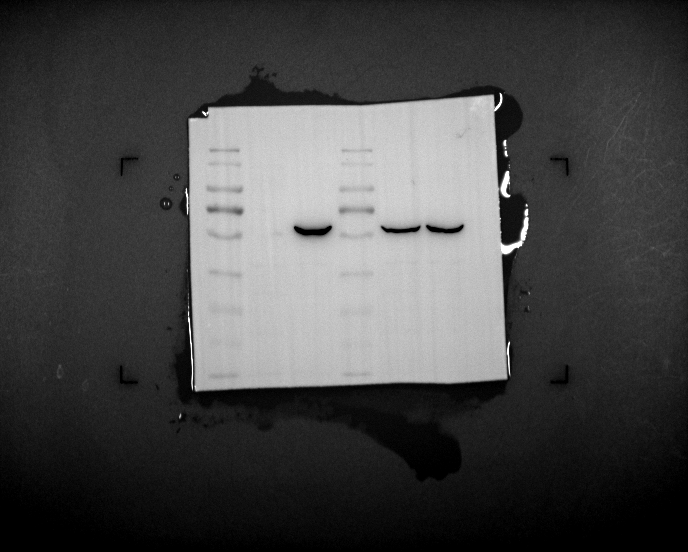

Supplement: Source data 1. [file elife-73220-data1.zip › source data (revision)/Figure 6-source data 3/Fig. 6C input HA.Tif]

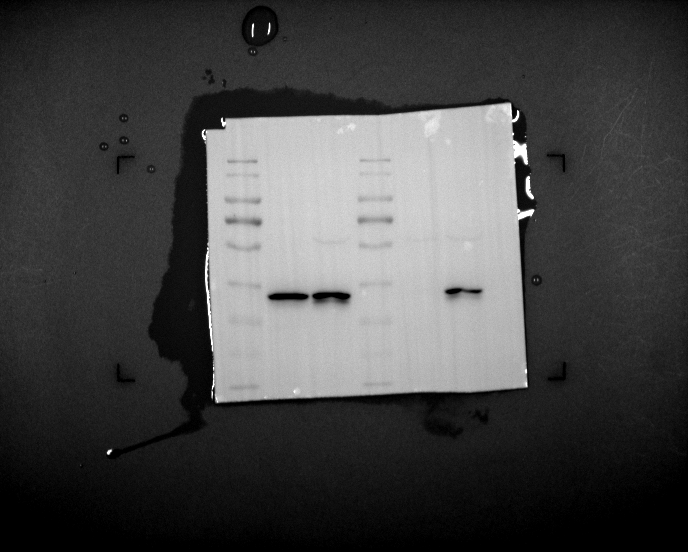

Supplement: Source data 1. [file elife-73220-data1.zip › source data (revision)/Figure 6-source data 3/Fig. 6C input flag.Tif]

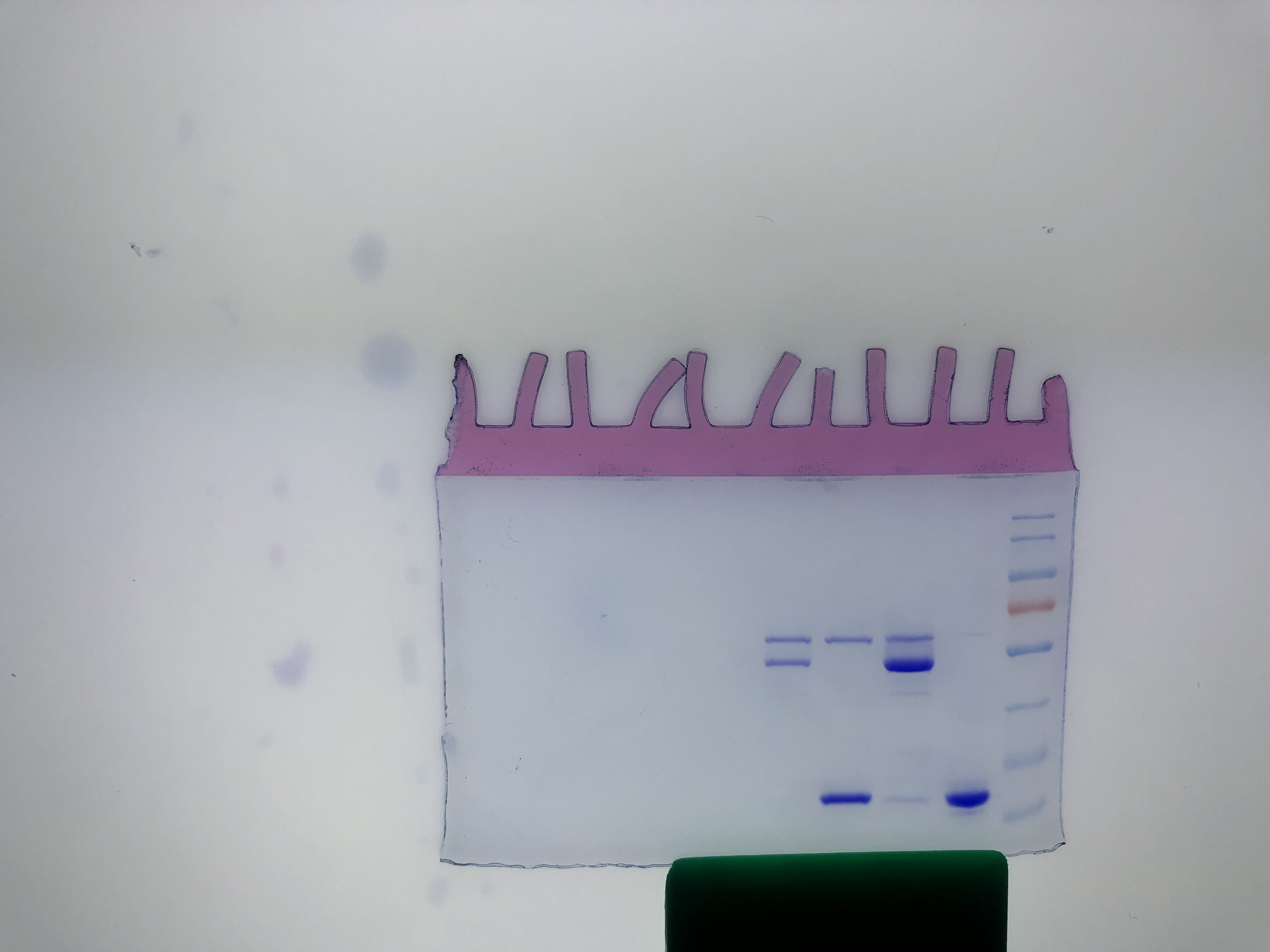

Supplement: Source data 1. [file elife-73220-data1.zip › source data (revision)/Figure 6-source data 4/Fig. 6D.jpg]

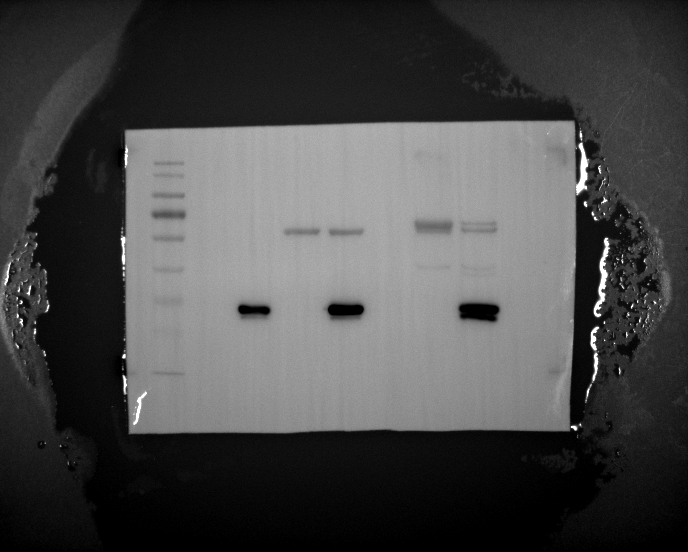

Supplement: Source data 1. [file elife-73220-data1.zip › source data (revision)/Figure 6-source data 5/Fig. 6E 14-3-3.Tif]

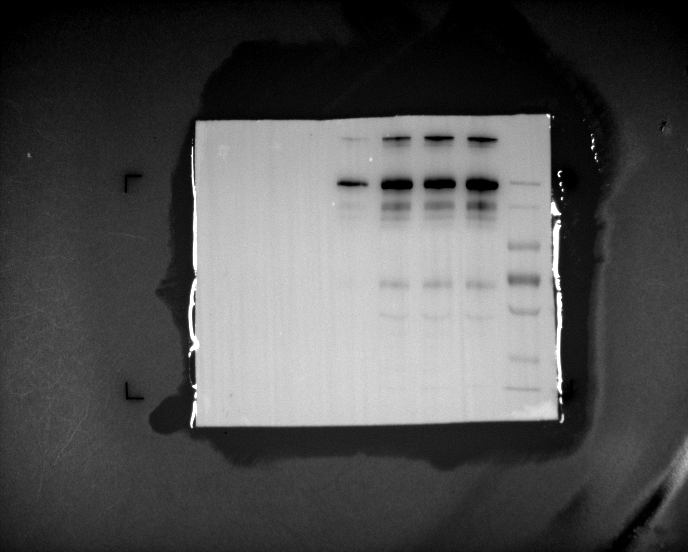

Supplement: Source data 1. [file elife-73220-data1.zip › source data (revision)/Figure 6-source data 5/Fig. 6E HA.Tif]

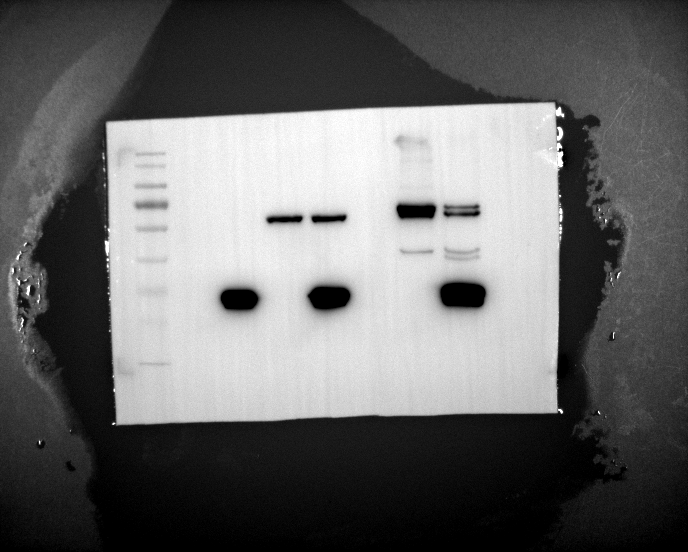

Supplement: Source data 1. [file elife-73220-data1.zip › source data (revision)/Figure 6-source data 5/Fig. 6E Lem8.Tif]

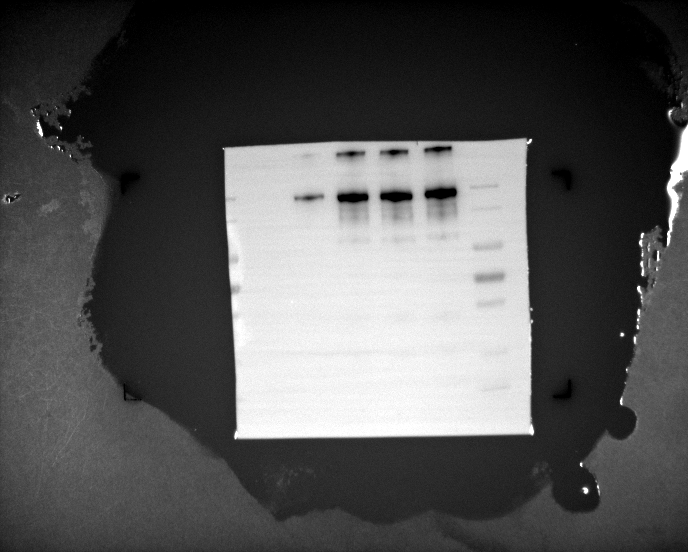

Supplement: Source data 1. [file elife-73220-data1.zip › source data (revision)/Figure 6-source data 5/Fig. 6E flag.Tif]

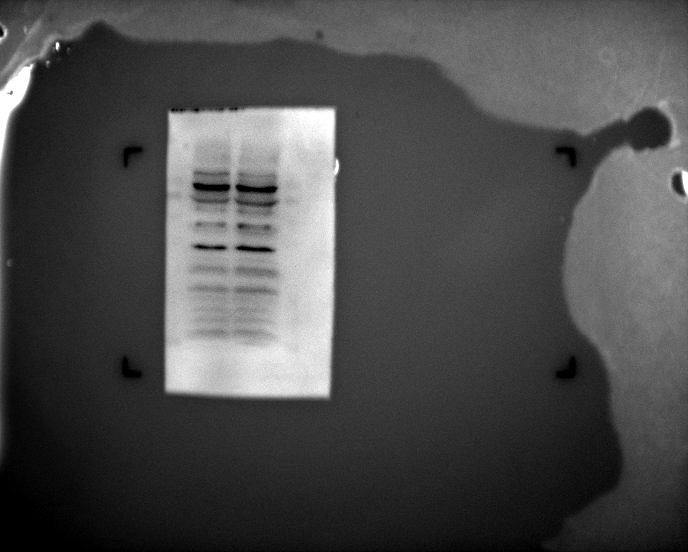

Supplement: Source data 1. [file elife-73220-data1.zip › source data (revision)/Figure 7-figure supplement 1-source data 1/Figure 7-figure supplement 1-A Lem8.Tif]

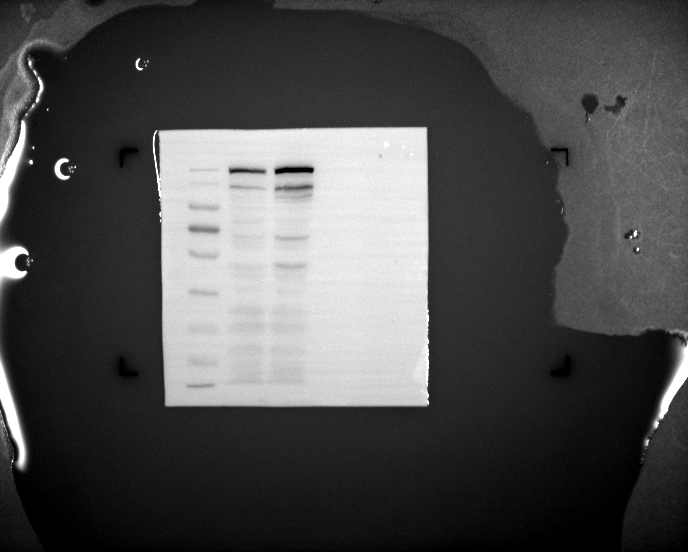

Supplement: Source data 1. [file elife-73220-data1.zip › source data (revision)/Figure 7-figure supplement 1-source data 1/Figure 7-figure supplement 1-A Phldb2.Tif]

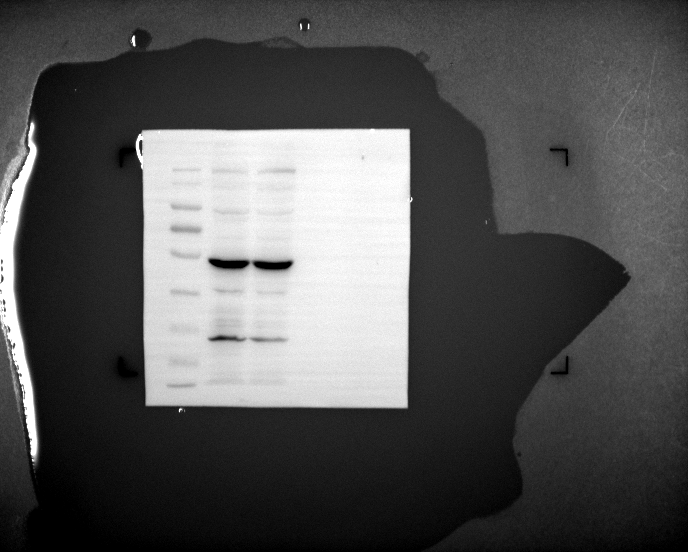

Supplement: Source data 1. [file elife-73220-data1.zip › source data (revision)/Figure 7-figure supplement 1-source data 1/Figure 7-figure supplement 1-A Tubulin.Tif]

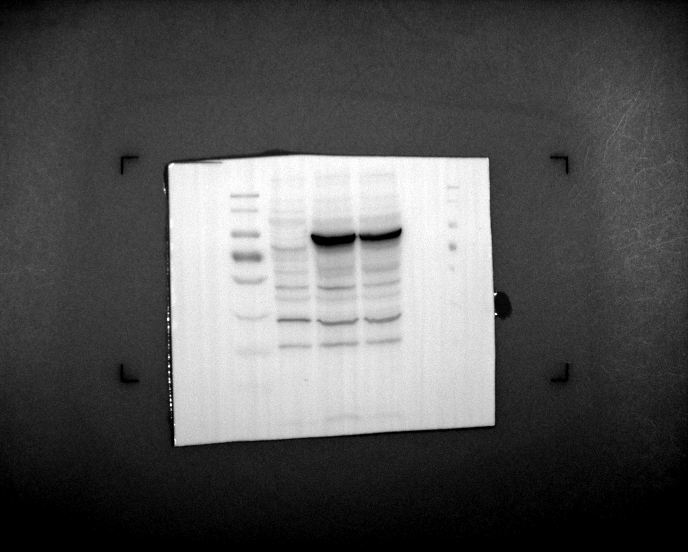

Supplement: Source data 1. [file elife-73220-data1.zip › source data (revision)/Figure 7-source data 1/Fig. 7A Lem8.Tif]

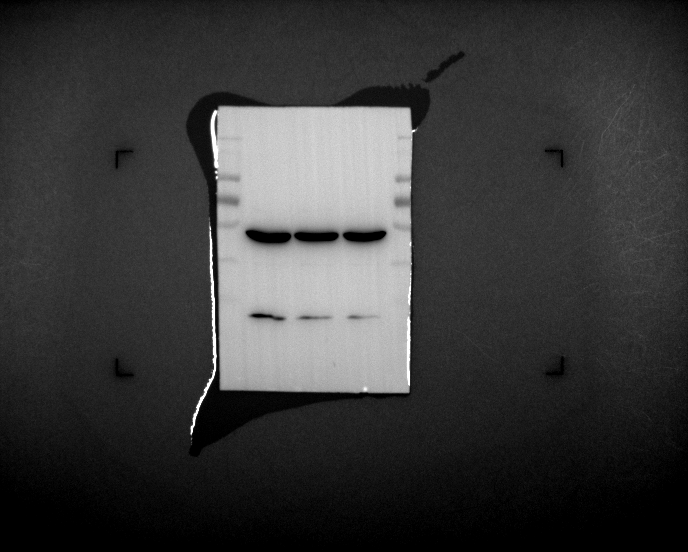

Supplement: Source data 1. [file elife-73220-data1.zip › source data (revision)/Figure 7-source data 1/Fig. 7A Tubulin.Tif]

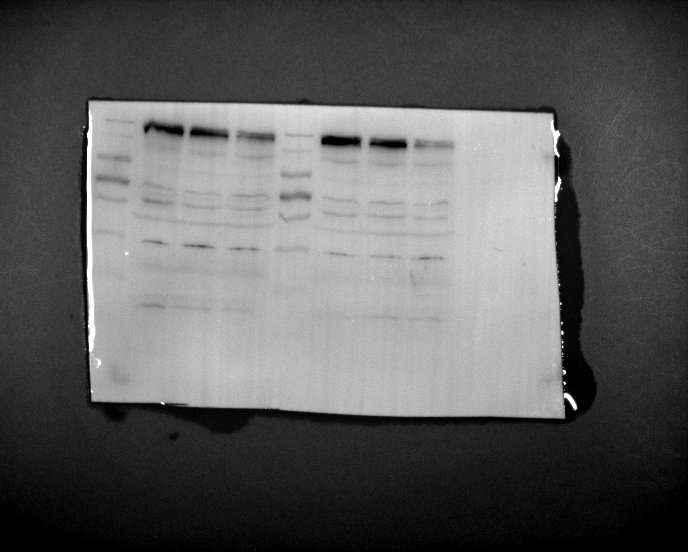

Supplement: Source data 1. [file elife-73220-data1.zip › source data (revision)/Figure 7-source data 1/Fig. 7A phldb2.Tif]
